# Safety and immunogenicity of the *Na*-GST-1 hookworm vaccine in Brazilian and American adults

**DOI:** 10.1371/journal.pntd.0005574

**Published:** 2017-05-02

**Authors:** David J. Diemert, Janaína Freire, Vanderson Valente, Carlos Geraldo Fraga, Frederico Talles, Shannon Grahek, Doreen Campbell, Amar Jariwala, Maria Victoria Periago, Martin Enk, Maria Flávia Gazzinelli, Maria Elena Bottazzi, Robert Hamilton, Jill Brelsford, Anna Yakovleva, Guangzhao Li, Jin Peng, Rodrigo Correa-Oliveira, Peter Hotez, Jeffrey Bethony

**Affiliations:** 1 Department of Microbiology, Immunology and Tropical Medicine, School of Medicine and Health Sciences, The George Washington University, Washington DC, United States of America; 2 Centro de Pesquisas René Rachou, Fundação Oswaldo Cruz, Belo Horizonte, Minas Gerais, Brazil; 3 School of Nursing, Federal University of Minas Gerais, Belo Horizonte, Minas Gerais, Brazil; 4 Department of Pediatrics, Section of Pediatric Tropical Medicine, Sabin Vaccine Institute and Texas Children's Hospital Center for Vaccine Development, National School of Tropical Medicine, Baylor College of Medicine, Houston, TX, United States of America; 5 Johns Hopkins University School of Medicine, Baltimore, MD, United States of America; QIMR Berghofer Medical Research Institute, AUSTRALIA

## Abstract

*Necator americanus* Glutathione-S-Transferase-1 (*Na*-GST-1) plays a role in the digestion of host hemoglobin by adult *N*. *americanus* hookworms. Vaccination of laboratory animals with recombinant *Na*-GST-1 is associated with significant protection from challenge infection. Recombinant *Na*-GST-1 was expressed in *Pichia pastoris* and adsorbed to aluminum hydroxide adjuvant (Alhydrogel) according to current Good Manufacturing Practice. Two Phase 1 trials were conducted in 142 healthy adult volunteers in the United States and Brazil, first in hookworm-naïve individuals and then in residents of a *N*. *americanus* endemic area in Brazil. Volunteers received one of three doses of recombinant *Na*-GST-1 (10, 30, or 100 μg) adjuvanted with Alhydrogel, adjuvanted with Alhydrogel and co-administered with an aqueous formulation of Glucopyranosyl Lipid A (GLA-AF), or the hepatitis B vaccine. Vaccinations were administered via intramuscular injection on days 0, 56, and 112. *Na*-GST-1/Alhydrogel was well tolerated in both hookworm-naïve and hookworm-exposed adults, with the most common adverse events being mild to moderate injection site pain and tenderness, and mild headache and nausea; no vaccine-related severe or serious adverse events were observed. Antigen-specific IgG antibodies were induced in a dose-dependent fashion, with increasing levels observed after each vaccination in both trials. The addition of GLA-AF to *Na*-GST-1/Alhydrogel did not result in significant increases in specific IgG responses. In both the US and Brazil studies, the predominant IgG subclass induced against *Na*-GST-1 was IgG1, with lesser amounts of IgG3. Vaccination of both hookworm-naïve and hookworm-exposed adults with recombinant *Na*-GST-1 was safe, well tolerated, and resulted in significant antigen-specific IgG responses. Based on these results, this vaccine will be advanced into clinical trials in children and eventual efficacy studies.

**Trial registration:**

ClinicalTrials.gov (NCT01261130 for the Brazil trial and NCT01385189 for the US trial)

## Author summary

Hookworm infection caused by *Necator americanus* is a major neglected tropical disease with significant associated morbidity. New tools, such as vaccines, are needed due to the inadequacy of current control strategies. Glutathione-S-Transferase-1 of *N*. *americanus* (*Na*-GST-1) is one of the lead hookworm vaccine candidates; antibodies induced by this vaccine are postulated to interfere with the digestion of host hemoglobin by adult *N*. *americanus* hookworms, thereby impairing their development and survival. We conducted two Phase 1 trials of recombinant *Na*-GST-1 adjuvanted with Alhydrogel in 142 healthy adults living in the United States and Brazil. Each participant received three vaccinations every 2 months by intramuscular injection of the vaccine administered with or without an aqueous solution of the Toll-like receptor-4 agonist, Glucopyranosyl Lipid A (GLA-AF). *Na*-GST-1/Alhydrogel was well tolerated in both hookworm-exposed and hookworm-naïve adults; no vaccine-related severe or serious adverse events were observed. Antigen-specific IgG antibodies were induced in a dose-dependent fashion with increasing levels observed after each vaccination. The addition of GLA-AF to the vaccine did not result in significantly higher antibody responses. Based on these results, the vaccine will be advanced into clinical trials in children and eventual efficacy studies.

## Introduction

Over 400 million people are infected with hookworm, predominantly in resource-limited tropical regions of the world [1]. Hookworm is a soil-transmitted nematode helminth that is primarily acquired after skin contact with infective larvae found in soil contaminated with human feces. Following penetration of the skin, larvae migrate through tissues before entering the gastrointestinal tract where they develop into adult worms that attach to the intestinal mucosa and feed on host blood [2]. Chronic infection, which often lasts for years, can result in pathology due to intestinal blood loss, with morbidity being proportional to the number of worms present in the host [3]. Heavier infections are more likely to result in iron-deficiency anemia, which impairs physical and intellectual development in children, negatively impacts birth outcomes, and is thought to significantly reduce future economic productivity [4]. New estimates of the global burden of disease caused by hookworm indicate that over 4 million disability-adjusted life years are lost annually and economic costs may exceed $100 billion [5].

Current hookworm control measures consist of regular mass drug administration with a benzimidazole anthelminthic such as albendazole or mebendazole [2]. However, this strategy has several limitations, including rapid re-infection following treatment due to continued exposure to larvae in the environment and the potential development of drug resistance [5]. A vaccine that would prevent acquisition of moderate or heavy intensity infections would therefore be a major advance in reducing morbidity associated with hookworm infection [6, 7]. Modeling the economic and epidemiologic impact of an effective hookworm vaccine predicts that when used in settings of high transmission, it would be both a highly cost-effective–and even cost-saving–biotechnology [5].

The survival of adult hookworms residing in the human intestine is dependent upon the degradation and utilization of host hemoglobin that the worms ingest during blood meals [8]. Following hemolysis of ingested erythrocytes, adult *N*. *americanus* hookworms use a series of hemoglobinases to cleave hemoglobin into smaller molecules that are used by the parasite to satisfy nutritional and biochemical requirements [8–13]. As a result of hemoglobin digestion, free heme and related products such as hematin are produced. Because both heme and hematin contain oxidative iron, they are potent generators of toxic reactive oxygen species that can potentially damage parasite macromolecules unless they are bound and detoxified by molecules such as glutathione S-transferase-1 (GST-1) [14–16]. GST-1 of *N*. *americanus* (*Na*-GST-1) is therefore a critical component of the blood-feeding pathway of this hookworm. When this antigen is produced as a recombinant protein and used as a vaccine, we hypothesize that it will induce neutralizing antibodies that will interfere with heme detoxification following blood digestion and thereby induce parasite death or reduce worm fecundity, thereby interrupting transmission.

*Na*-GST-1 is a 24-kDa protein with peroxidase enzymatic activity that catalyzes the conjugation of reduced glutathione to a variety of electrophiles [14–16]. This hookworm protein belongs to the Nu class of nematode GSTs, which is characterized by reduced peroxidase activity relative to other GST classes, but elevated binding capacity for heme and related molecules [14–18]. X-ray crystallography of *Na*-GST-1 demonstrates that the protein forms homodimers in solution, creating large binding grooves that are accessible to a variety of ligands, including free heme at high affinity *in vitro* [14, 15]. *In vivo*, hookworm GSTs may therefore bind and detoxify the heme and hematin byproducts generated during the blood degradation process.

Vaccination of hamsters and dogs and with recombinant *Na*-GST-1 or the canine homologue (*Ancylostoma caninum* GST-1 or *Ac*-GST-1), respectively, resulted in reduced worm burdens and fecal egg counts following challenge infection compared to control animals [14, 15, 19]. Based on the results of these preclinical studies, recombinant *Na*-GST-1 was chosen as a lead hookworm vaccine candidate. Two Phase 1 trials of the vaccine were first conducted in healthy adults living in the United States (US) and Brazil in volunteers with no prior exposure to hookworm and then in Brazilian volunteers living in an area endemic for *N*. *americanus*. At all three sites, recombinant *Na*-GST-1 formulated on Alhydrogel was tested with or without the point-of-injection addition of an aqueous formulation of the Toll-like receptor (TLR)-4 agonist, Glucopyranosyl Lipid A (GLA-AF). This TLR agonist was chosen based on preclinical studies in laboratory animals that showed it results in significant improvement in antigen-specific antibody responses.

## Methods

### Study vaccines

Recombinant *Na*-GST-1 was manufactured and adsorbed to Alhydrogel (Biosector, Denmark) as described previously [20]. Recombinant *Na*-GST-1 has been shown to assemble into homodimers, similar to the native form of the protein [16]. Each vial of *Na*-GST-1/Alhydrogel contained 1.35 mL consisting of recombinant protein (0.1 mg/mL) adsorbed to Alhydrogel (0.8 mg/mL) in glucose imidazole buffer (10% glucose, 10 mM imidazole, pH 7.4). Studies of vaccine stored at 2 to 8°C were conducted in mice every 6 months to confirm stability and potency.

GLA-AF (Infectious Diseases Research Institute, Seattle, WA) was supplied as a 50 μg/mL aqueous solution in multi-dose vials containing 25 μg of GLA without preservative, or as a 100 μg/mL aqueous solution in multi-dose vials containing 40 μg of GLA without preservative.

Hepatitis B vaccine (Instituto Butantan, Brazil) was supplied in multi-dose vials containing 10 ml of recombinant hepatitis B surface antigen adsorbed to amorphous aluminum hydroxyphosphate sulphate; each 1.0 ml dose of vaccine contained 10 μg of antigen. Vaccines were transported to the study site using temperature-monitoring devices to ensure maintenance of the cold chain.

### Study sites and populations

The studies reported herein were conducted in Washington, DC, and at two sites in the state of Minas Gerais, Brazil: Belo Horizonte and Americaninhas. The latter is situated in a mostly rural, hookworm-endemic region with approximately 1200 inhabitants in which the epidemiology of *N*. *americanus* and other helminth infections has previously been extensively reported [21–24]. Washington, DC, and Belo Horizonte are large urban areas that are non-endemic for hookworm.

### Phase 1 trials of *Na*-GST-1

Two Phase 1 trials of the *Na*-GST-1 Hookworm Vaccine were conducted between 2012 and 2015, one in Brazil and one in the US. The primary objective of both studies was to estimate the safety and reactogenicity of the vaccine in healthy adults between the ages of 18 and 45 years; immunological endpoints were assessed as secondary objectives. Volunteers were excluded if they had evidence of clinically significant systemic disease; were pregnant or breast feeding; had serological evidence of HIV, chronic hepatitis B or C infection; were receiving corticosteroids or immunosuppressive drugs; or had been immunized with a live vaccine within the previous month. For the study in Brazil, volunteers were also excluded if they tested positive for IgE antibodies to *Na*-GST-1 (see below).

The Phase 1 study in Brazil enrolled 102 participants. The trial was conducted in two parts: In Part I, 36 healthy, hookworm-naïve volunteers living in the urban center of Belo Horizonte, Brazil, received vaccinations of 10, 30, or 100 μg *Na*-GST-1/Alhydrogel administered with (n = 18) or without (n = 18) the point-of-injection addition of 2.5 μg GLA-AF. In Part II, which was conducted in a double-blind fashion, 66 healthy, hookworm-exposed volunteers living in the area in and around Americaninhas were enrolled into one of 6 cohorts and randomized to receive *Na*-GST-1/Alhydrogel, *Na*-GST-1/Alhydrogel plus GLA-AF, or the hepatitis B vaccine, as follows. In the first cohort, 10 volunteers received either 10 μg *Na*-GST-1/Alhydrogel (n = 8) or the hepatitis B vaccine (n = 2); in the second, 12 volunteers received either 10 μg *Na*-GST-1/Alhydrogel (n = 2) or 10 μg *Na*-GST-1/Alhydrogel plus 2.5 μg GLA-AF (n = 10); in the third cohort, 10 volunteers received either 30 μg *Na*-GST-1/Alhydrogel (n = 8) or the hepatitis B vaccine (n = 2); in the fourth cohort, 12 volunteers received either 30 μg *Na*-GST-1/Alhydrogel (n = 2) or 30 μg *Na*-GST-1/Alhydrogel plus 2.5 μg GLA-AF (n = 10); in the fifth cohort, 10 volunteers received either 100 μg *Na*-GST-1/Alhydrogel (n = 8) or the hepatitis B vaccine (n = 2); and, in the sixth cohort, 12 volunteers received either 100 μg *Na*-GST-1/Alhydrogel (n = 2) or 100 μg *Na*-GST-1/Alhydrogel plus 2.5 μg GLA-AF (n = 10). As part of the study design, four individuals who were either diagnosed with hookworm infection during screening or had a documented infection within 3 months of the first vaccination were enrolled into each cohort of Part II, providing their infections had been treated.

The Phase 1 study in the US enrolled 40 participants into 1 of 5 cohorts. Hookworm-naïve adults living in the Washington, DC metropolitan area were vaccinated with *Na*-GST-1/Alhydrogel administered with or without up to 5 μg GLA-AF: in the first cohort, 12 volunteers were vaccinated with either 10 μg *Na*-GST-1/Alhydrogel (n = 4) or 10 μg *Na*-GST-1/Alhydrogel plus 1 μg GLA-AF (n = 8); in the second cohort, 12 volunteers were vaccinated with either 30 μg *Na*-GST-1/Alhydrogel (n = 4) or 30 μg *Na*-GST-1/Alhydrogel plus 1 μg GLA-AF (n = 8); in the third cohort, 4 volunteers were vaccinated with 30 μg *Na*-GST-1/Alhydrogel plus 5 μg GLA-AF; in the fourth cohort, 4 volunteers were vaccinated with 100 μg *Na*-GST-1/Alhydrogel; and in the fifth cohort, 10 volunteers were vaccinated with 100 μg *Na*-GST-1/Alhydrogel plus 5 μg GLA-AF. The first two cohorts were enrolled, randomized and vaccinated in a double-blind fashion, whereas the last three were enrolled in an open-label fashion.

All vaccinations in both studies were administered via intramuscular injection in the deltoid muscle on study days 0, 56, and 112. For cohorts in which vaccines were administered in a double-blind fashion, syringe barrels were masked with opaque tape to disguise the contents and injections were administered by individuals who were not involved in post-vaccination safety assessments or study analysis. All volunteers were followed for 12 months after the final injection. In both studies, randomization was conducted using computer-generated sequences provided to the study pharmacist in sealed envelopes by a third party.

For both studies, the safety of participants was monitored by an independent physician and a safety monitoring committee that reviewed interim safety reports at regular intervals prior to dose escalation. In both parts of the study in Brazil and in the US trial, cohorts were enrolled and vaccinated in a staggered fashion in which the safety of lower doses of *Na*-GST-1/Alhydrogel was assessed prior to vaccinating with higher doses.

### Ethics statement

The study in Brazil was approved by the institutional review boards (IRBs) of the Centro de Pesquisas René Rachou, the George Washington University (GWU), and the Brazilian Ministry of Health, whereas the trial in the US was approved by the IRBs of GWU and Children’s National Medical Center. Both trials were conducted under an investigational new drug application (BB-14616) to the US Food and Drug Administration; for the study in Brazil, regulatory oversight was also provided by the Agencia Nacional de Vigilância Sanitária of the Brazilian federal government (approval #934290/11-7). The trials were registered at Clinicaltrials.gov, NCT01261130 (Brazil) and NCT01385189 (US). For both studies, written informed consent was obtained from all volunteers.

### Assessment of safety and tolerability

Following vaccinations in both Phase 1 trials, volunteers were observed for at least 120 minutes and then evaluated 1 (in Brazil only), 3, 7, 14, and 28 days post-vaccination for evidence of local and systemic reactogenicity, and then at regular intervals until 12 months after the final vaccination. At each visit, injection sites were examined for erythema, swelling, and tenderness. Solicited systemic adverse events included fever, headache, nausea, vomiting, myalgia, arthralgia, and urticaria. Adverse events were graded as mild (easily tolerated), moderate (interfered with activities of daily living), or severe (prevented activities of daily living), and assigned causality relative to the study vaccine. Injection site erythema and swelling were graded as mild (>0 to ≤25 mm in diameter), moderate (>25 to ≤50 mm), or severe (>50 mm); and, oral temperature as mild (≥38.0°C to <38.5°C), moderate (≥38.5°C to <39.0°C), or severe (≥39.0°C). A complete blood count, serum creatinine, and alanine aminotransferase were performed immediately prior to vaccination as well as on the fourteenth day following each vaccination. Abnormal clinical laboratory findings were graded as mild, moderate or severe using standardized toxicity tables.

Due to the use of the GLA-AF immunostimulant as a component added to the experimental *Na*-GST-1/Alhydrogel vaccine, the incidence of the following adverse events of special interest were actively monitored throughout both clinical trials: neuroinflammatory disorders (e.g., optic neuritis, multiple sclerosis, Guillain-Barré syndrome), autoimmune musculoskeletal disorders (e.g., systemic lupus erythematosus, Sjögren’s syndrome, rheumatoid arthritis), gastrointestinal disorders (e.g., inflammatory bowel disease), metabolic diseases (e.g., autoimmune thyroiditis), vasculitides, and other autoimmune or inflammatory diseases.

### Antibody measurements

#### Measurement of anti-*Na*-GST-1 IgG and IgG1 antibodies

*Na*-GST-1 specific IgG and IgG1 antibodies were measured in serum samples collected from participants of the two Phase 1 trials by qualified indirect ELISA. A homologous standard reference serum (SRS) of IgG and IgG1 against *Na*-GST-1 was made by pooling sera collected from high IgG and IgG1 responders (OD_492nm_ ≥ 1.000) from Study Day 126 (two weeks after the third vaccination) from hookworm-naïve study participants in Belo Horizonte who received 100 μg *Na*-GST-1/Alhydrogel (with or without GLA-AF). The SRS was serially diluted in duplicate along 11 columns of the first two rows of each ELISA plate for IgG (Nunc Polysorb) and IgG1 (Nunc Maxisorb) to generate a dilution-response curve that was modeled into a standard calibration curve (SCC) by a four-parameter logistic-log function as described by us previously [25, 26]. The parallelism of the SCCs was assessed by an Analysis of Variance (ANOVA) test as part of the qualification of the SRS, after the SCCs were linearized through transformation [27]. Arbitrary Units (AU) of anti-*Na*-GST-1 IgG and IgG1 were obtained as described in Jariwala et al [25, 26] and [28]. Briefly, test serum samples were added in duplicate to 96-well microtiter plates (Nunc Maxisorb) at a 1:1000 dilution for both IgG and IgG1. Horseradish peroxidase conjugate secondary antibodies were added at 1:1,000 for both IgG (Southern Biotech) and IgG1 (Life technologies). All indirect ELISA plates were developed using o-phenylenediamine dihydrochloride (OPD) (Sigma Aldrich) for 30 minutes in the dark at room temperature and read at OD_492nm_ on a SpectraMax Plus 384 Microplate Reader (Molecular Devices), with data collected using SOFTmax GXP PRO version 4 (Molecular Devices). The mean OD_492nm_ of test sera duplicates were then interpolated onto the SCC to derive the AU of anti-*Na*-GST-1 IgG or anti-*Na*-GST-1 IgG1. The values of the Limit of Quantitation (LOQ) for the anti-*Na*-GST-1 IgG and anti-*Na*-GST-1 IgG1 assays were obtained from the SCCs as discussed in detail in [25].

#### Measurement of anti-*Na*-GST-1 IgG3 and IgE antibodies

Heterologous interpolation, as described in [28], was used to derive AU values of anti*-Na*-GST-1 IgG3 and anti-*Na*-GST-1 IgE antibodies. Heterologous SCCs of human IgG3 and IgE against *Na*-GST-1 were made by serial dilution, in duplicate, of human myeloma derived IgG3 (Athens Research) and human myeloma derived IgE (Abbiotec) directly onto 11 columns of the first two rows of each ELISA plate (Nunc Maxisorb) to generate a dilution–response curve that was modeled by a four-parameter logistic-log function [25]. Test serum samples were then added in duplicate at a 1:50 dilution for IgG3 and a 1:25 dilution for IgE and incubated overnight in the dark at 4°C. Horseradish peroxidase conjugate secondary antibodies were added at the following dilutions: 1:1000 for IgG3 (Life technologies) and 1:1000 for IgE (Hybridoma Reagent Laboratory). Microtiter plates were then removed, washed and developed using OPD (Sigma Aldrich) for 30 minutes in the dark at room temperature and read at OD_492nm_ on a SpectraMax Plus 384 Microplate Reader (Molecular Devices), with data collected using SOFTmax GXP PRO version 4 (Molecular Devices). The mean OD_492nm_ of test sera duplicates were then interpolated onto the SCC to derive AU values of anti-*Na*-GST-1 IgG3 or anti-*Na*-GST-1 IgE. The values of the LOQ for the anti-*Na*-GST-1 IgG3 or anti-*Na*-GST-1 IgE assays were obtained from each SCC as discussed in [25].

### Seroepidemiology survey for IgE against *Na*-GST-1 in Brazilian volunteers

Prior to initiation of the Phase 1 trials of *Na*-GST-1/Alhydrogel, stored serum samples collected from children and adults living in Americaninhas were tested for IgE antibodies against *Na*-GST-1. These sera had been collected from individuals enrolled in previously reported clinical studies of helminth epidemiology, treatment and transmission [24]. IgE antibody levels against *Na*-GST-1 were measured by using the fluoroenzyme immunoassay (ImmunoCAP 250; Phadia, Uppsala Sweden) at the Johns Hopkins Dermatology, Allergy and Clinical Immunology Reference Laboratory. IgE levels greater than 0.1 kU/L (0.24 ng/dL) were considered clinically significant.

### Statistical analysis

The proportions of participants experiencing each adverse event (of any severity) were tabulated by vaccine allocation (*Na*-GST-1 *vs*. hepatitis B vaccine) as well as by dose and formulation of *Na*-GST-1. Testing for differences in incidence of adverse events among groups was performed using Fisher’s Exact Test due to the low frequency of most events.

Analyses of antibody levels to *Na*-GST-1 were conducted using non-parametric Mann-Whitney-Wilcoxon U tests to determine if there were differences in antibody levels between dose groups by study day. When the test produced a statistically significant result (p < 0.05), individual pair-wise tests were performed and significance was assessed using the Holm-Bonferroni adjustment. To compare antibody levels between participants in these two studies, changes in individual antibody levels were computed for each participant by subtracting the baseline antibody level (i.e., on Study Day 0) from each subsequent measurement. Changes in antibody levels were then compared between participants of the two studies on each study day using the Kruskal-Wallis test.

Another approach taken was to evaluate changes in IgG antibody levels elicited by the three different dose concentrations of *Na*-GST-1 over time, in comparison to the hepatitis B vaccine, by pooling results from both studies. This was done after verifying that there were no significant differences in changes in antibody levels between individuals vaccinated with comparable doses of *Na*-GST-1 in the two different studies, as outlined above. Data were analyzed using SAS software (version 9.3; SAS Institute).

## Results

### Participant flow and baseline data

**Fig 1:**
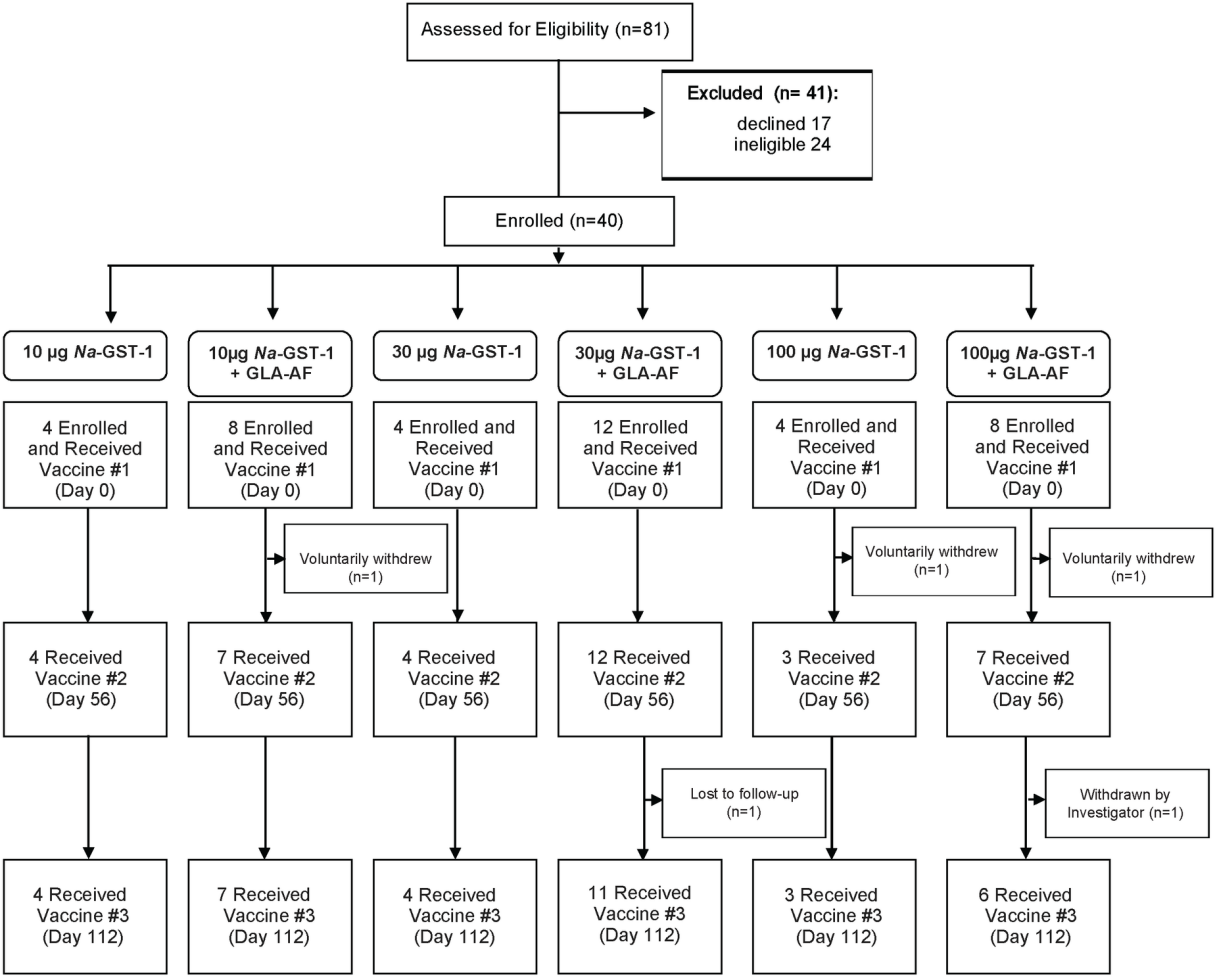
Study flow diagram for US clinical trial.

Of 81 adults screened for inclusion in the Phase 1 trial of the *Na*-GST-1/Alhydrogel hookworm vaccine in the US, 17 declined or were lost to follow-up before enrollment, 24 were ineligible, and 40 (24 males and 16 females) met eligibility criteria and were enrolled in the study (**Fig 1**). Reasons for exclusion were: concomitant medical diagnoses such as uncontrolled hypertension, asthma, major psychiatric illness, and diabetes mellitus (n = 7); and, abnormal screening laboratory exams (n = 17). The median age of enrolled participants was 34.5 years (range, 18–45). Of the 40 who were enrolled, 3 withdrew for personal reasons or were lost to follow-up after receiving the first vaccination, while 1 withdrew and 1 was withdrawn due to non-compliance after receiving the first two vaccinations; no participants withdrew due to the occurrence of an adverse event.

**Fig 2:**
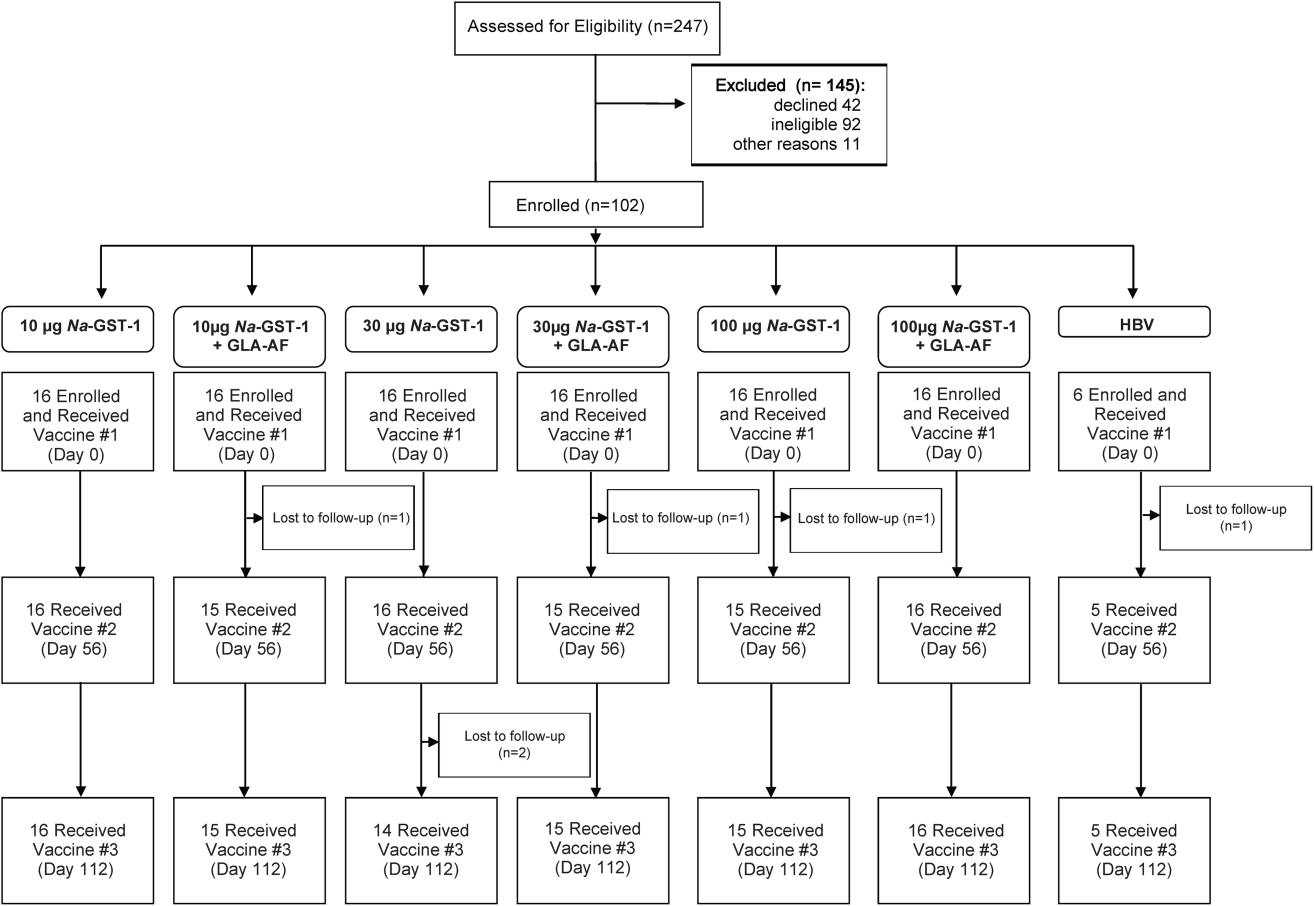
Study flow diagram for Brazilian clinical trial.

Of 247 adults screened for inclusion in the Phase 1 trial of *Na*-GST-1 in Brazil, 42 declined or were lost to follow-up before enrollment, 92 were ineligible, and 102 (49 males and 53 females) were deemed eligible and enrolled; another 11 were eligible but were not enrolled as they had not recently been treated for hookworm infection and therefore did not meet the protocol-mandated requirement of four recently treated volunteers per cohort (**Fig 2**). Reasons for exclusion were prior receipt of hepatitis B vaccine (n = 6), intent to move from the study area during the study period (n = 11), concomitant medical diagnoses (n = 49), and abnormal screening laboratory examinations (n = 16). The median age of enrolled participants was 26 years (range, 18–45). As planned according to the study protocol, twelve participants were enrolled who had been treated for hookworm within 120 days of receiving their first vaccination, to ensure safety would be evaluated in this sub-population. Of the 102 who were enrolled, 4 withdrew for personal reasons after receiving the first vaccination and 1 withdrew after receiving the first two vaccinations; no participants withdrew due to the occurrence of an adverse event.

Baseline age, gender and body mass index distributions for the two clinical trials are shown as Supplementary Information (**S1 Table** and **S2 Table**).

### Safety

Overall, the *Na*-GST-1/Alhydrogel vaccine, whether or not co-administered with GLA-AF, was well tolerated by study participants in both the US and Brazil. No participants were withdrawn or had vaccinations suspended due to significant reactogenicity or unsolicited adverse events. Vaccine-related serious adverse events were not reported, and none of the vaccine recipients developed adverse events of special interest. In both Brazilian and American healthy adults, the most common injection site reactions were mild to moderate pain and tenderness (**Table 1**). Severe injection site pain and tenderness were observed in 1 participant in Belo Horizonte starting on the same day as the second vaccination with 10 μg *Na*-GST-1/Alhydrogel co-administered with 2.5 μg GLA-AF; these lasted for 2 days and recurred following the third vaccination although only at mild intensity. The same participant experienced severe arthralgias and myalgias for 1 day following the second vaccination. Severe injection site pain of 3 days’ duration was reported in 1 volunteer in Belo Horizonte after the first vaccination with 100 μg *Na*-GST-1/Alhydrogel co-administered with 2.5 μg GLA-AF, which did not recur at this intensity with subsequent vaccinations. Finally, 1 volunteer in Washington, DC, experienced 3 days of severe injection site tenderness after the second vaccination with 10 μg *Na*-GST-1/Alhydrogel administered with 1 μg GLA-AF, without recurrence after the third vaccination.

**Table 1:** Solicited local injection site and systemic adverse events after vaccination with *Na*-GST-1/Alhydrogel (with or without GLA-AF) or the hepatitis B vaccine in US and Brazilian adults. Data are number (%) of participants experiencing an event after any vaccination. Results for the different doses of *Na*-GST-1 and GLA-AF are combined. Participants with more than one occurrence of the same adverse event are recorded only once, at the maximum severity experienced.

	*Na*-GST-1/Alhydrogel (n = 60)			*Na*-GST-1/Alhydrogel + GLA-AF (n = 76)			HBV Vaccine (n = 6)		
	Mild	Moderate	Severe	Mild	Moderate	Severe	Mild	Moderate	Severe
	N (%)	N (%)	N (%)	N (%)	N (%)	N (%)	N (%)	N (%)	N (%)
**Local**									
Pain	17 (28)	5 (8)	–	48 (63)	8 (11)	2 (3)	–	–	–
Tenderness	16 (27)	8 (13)	–	36 (47)	15 (20)	2 (3)	1 (17)	–	–
Swelling	4 (7)	1 (2)	–	4 (5)	1 (1)	–	–	–	–
Erythema	1 (2)	1 (2)	–	1 (1)	2 (3)	–	–	–	–
**Systemic**								–	–
Headache	14 (23)	5 (8)	4 (7)	23 (30)	8 (11)	–	–	2 (33)	–
Nausea	9 (15)	1 (2)	–	12 (16)	4 (5)	–	–	–	–
Vomiting	5 (8)	–	–	3 (4)	3 (4)	–	–	–	–
Myalgia	6 (10)	1 (2)	–	4 (5)	1 (1)	1 (1)	–	–	–
Arthralgia	2 (3)	–	–	1 (1)	–	1 (1)	–	–	–
Fever	3 (5)	–	2 (3)	1 (1)	–	1 (1)	–	–	–
Urticaria	–	–	–	–	–	–	–	–	–

The most frequently reported solicited systemic events in both trials were mild headache and nausea (**Table 1**). Severe headache occurred in 4 participants who received *Na*-GST-1/Alhydrogel without GLA-AF (1 who received 10 μg *Na*-GST-1, 2 who received 30 μg and 1 who received 100 μg), although only one of these events was considered possibly related to study product: this participant had 1 day of severe headache 3 days after being vaccinated with the first dose of 30 μg *Na*-GST-1/Alhydrogel that resolved without intervention. Three participants had severe fever following vaccination, 1 of which was judged possibly related to vaccination and lasted less than 24 hours.

When comparing injection site reactions, no differences were seen between participants at the three study sites or between those who were hookworm-naïve and those who were hookworm-exposed. However, those who received *Na*-GST-1/Alhydrogel in combination with GLA-AF had significantly more occurrences of pain, swelling, and tenderness than those who were vaccinated with *Na*-GST-1/Alhydrogel without GLA-AF (p<0.0001), although the severity of these reactions was not significantly different between the two groups. In contrast, no significant differences were seen in the incidence of headache or nausea between those who received *Na*-GST-1/Alhydrogel in combination with GLA-AF and those who received it without GLA-AF. Due to low numbers of the other solicited systemic adverse events, no statistical comparisons could be made; however, there did not appear to be any obvious trends by dose of vaccine or co-administration with GLA-AF.

The most common clinical laboratory abnormalities observed at all 3 study sites were decreases in hemoglobin from baseline (i.e., the day of first vaccination). In Washington, DC, 6 of 40 volunteers had at least 1 reduction from baseline during the 16-month follow-up, 5 of which were mild whereas 1 was severe (a decrease of 4.4 g/dl). Of these, only 1 had a hemoglobin concentration (8.3 g/dl) that was below the lower reference limit for the testing laboratory (10.7 g/dl), which met the definition of a severe adverse event; however, this was judged unlikely to be related to vaccination given concomitant menorrhagia secondary to uterine fibroids that eventually required embolization. In the Brazilian study, 61 of 102 participants had a total of 87 episodes of decreased hemoglobin from baseline of more than 0.6 mg/dl during the 16-month follow-up. Of these, 61 were mild, 22 moderate and 4 severe. Of the severe events, 2 were deemed possibly related to vaccination whereas 2 were graded as being unlikely related due to concomitant diagnoses. Furthermore, there were only 19 instances of hemoglobin concentrations below the lower reference limit, all of which were mild in severity and only 6 of which were considered possibly related to vaccination. Of note, there were no significant differences in incidence or severity of decreases in hemoglobin concentration between doses of *Na*-GST-1, coadministration status with GLA-AF, or, most importantly, compared to the hepatitis B vaccine group. Other clinical laboratory abnormalities were uncommon and only 3 were graded as moderate, 1 of which (decrease in absolute neutrophil count in a volunteer in the US trial who received 100 μg *Na*-GST-1/Alhydrogel without GLA-AF) was considered possibly related to vaccination (Table 2).

**Table 2:** Clinical laboratory adverse events after vaccination with *Na*-GST-1/Alhydrogel (with or without GLA-AF) or the hepatitis B vaccine in US and Brazilian adults. Data are number (%) of participants experiencing an event after any vaccination. Results for the different doses of *Na*-GST-1 and GLA-AF are combined. Participants with more than one occurrence of the same adverse event are recorded only once, at the maximum severity experienced.

	*Na*-GST-1/Alhydrogel (n = 60)			*Na*-GST-1/Alhydrogel + GLA-AF (n = 76)			HBV Vaccine (n = 6)	
	Mild	Moderate	Severe	Mild	Moderate	Severe	Mild	Moderate/Severe
	N (%)	N (%)	N (%)	N (%)	N (%)	N (%)	N (%)	N (%)
Low Hemoglobin Concentration	10 (17)	–	–	7 (9)	–	1 (1)	–	–
Reduction in Hemoglobin from Baseline*	24 (40)	9 (15)	1 (2)	21 (28)	8 (11)	4 (5)	1 (17)	1 (17)
Increased WBC	6 (10)	–	–	10 (13)	–	–	1 (17)	–
Decreased WBC	1 (2)	–	–	4 (5)	–	–	–	–
Decreased ANC	3 (5)	1 (2)	–	1 (1)	1 (1)	–	–	–
Decreased Platelets	–	–	–	–	–	–	–	–
Increased ALT	2 (3)	–	–	4 (5)	–	–	–	–
Increased creatinine	2 (3)	–	–	1 (1)	–	–	–	–

HBV, hepatitis B virus; WBC, white blood cell count; ANC, absolute neutrophil count; ALT, alanine aminotransferase.

*From Study Day 0

In the Brazilian study, a total of 610 unsolicited adverse events were reported; 47 (44 mild; 3 moderate) were considered definitely, probably, or possibly related to the study product. The 3 moderate, related unsolicited adverse events were arm swelling and rash distal to the injection site in 1 participant beginning 1 day following the third vaccination with 100 μg *Na*-GST-1/Alhydrogel, and fatigue; all of these were transient, lasted 4 days or less, and resolved spontaneously without sequelae. In the US study, 171 unsolicited adverse events were reported of which 22 were considered possibly related to vaccination; 4 of these were moderate in intensity: 2 transient and asymptomatic increases in systolic blood pressure, 1 case of rhinorrhea, and 1 episode of lateral chest wall discomfort starting the day after receiving the second dose of 30 μg *Na*-GST-1/Alhydrogel without GLA-AF. Significant differences were not observed in either study in the incidence of unsolicited adverse events between the different doses of *Na*-GST-1 or between formulations (i.e., no GLA-AF *vs*. co-administered with GLA-AF).

### Serological survey of adults and children living in a *N*. *americanus* endemic area

No individuals in the serological survey (n = 179) conducted prior to the initiation of the Phase 1 trial in the hookworm endemic area of Americaninhas had clinically significant levels of IgE against *Na*-GST-1 as determined by the ImmunoCAP method (**S1 Fig**). None of the individuals tested had a level of IgE to *Na*-GST-1 above the clinical threshold of 0.1 kU/L (0.24 ng/dL).

### Antibody responses to *Na*-GST-1 prior to vaccination

**Fig 3:**
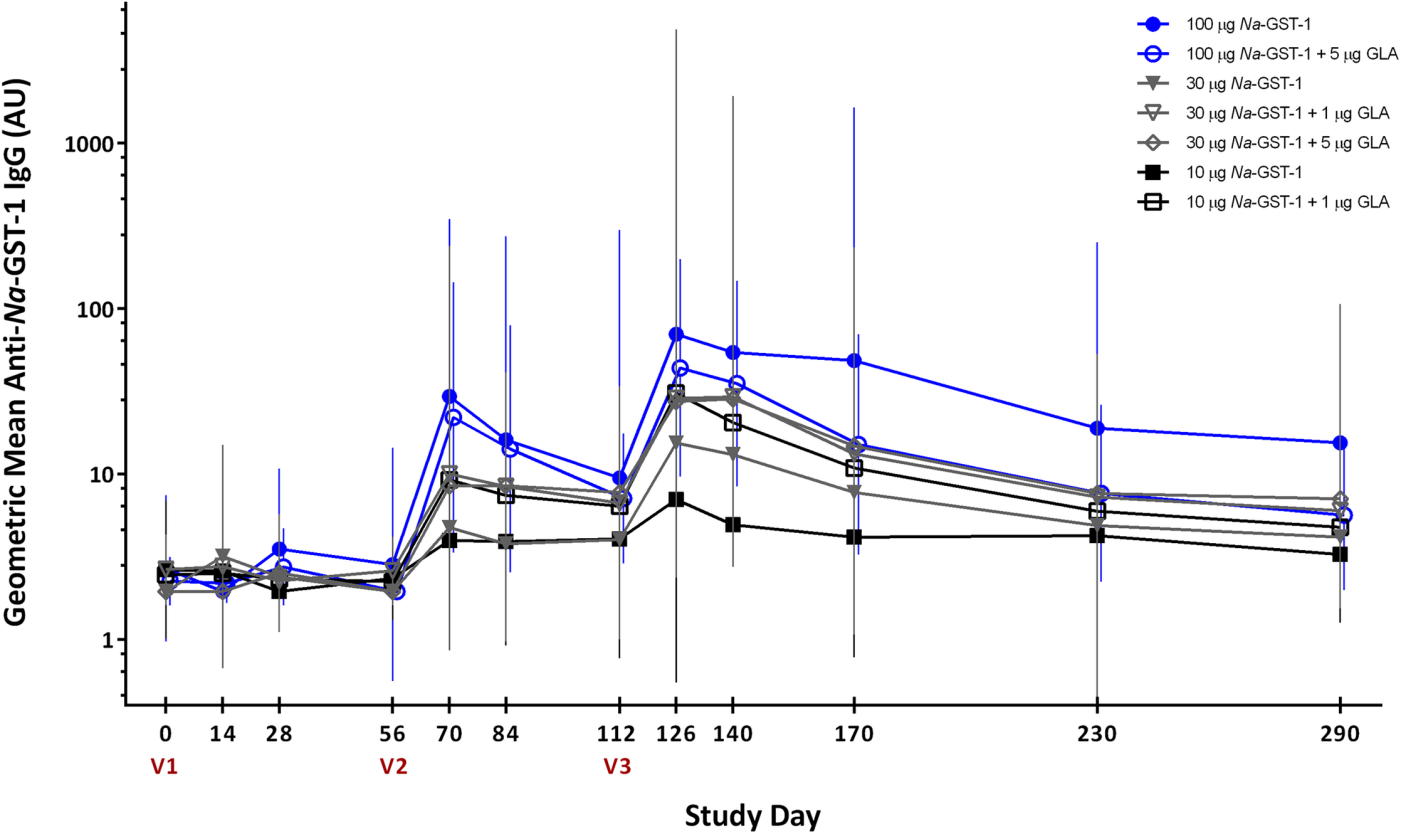
Geometric mean levels of IgG against recombinant *Na*-GST-1 in US participants immunized with *Na*-GST-1/Alhydrogel (administered with or without GLA-AF) as measured by ELISA. Bars represent 95% confidence intervals. V1 = first vaccination; V2 = second vaccination; V3 = third vaccination; AU = arbitrary units.

**Fig 4:**
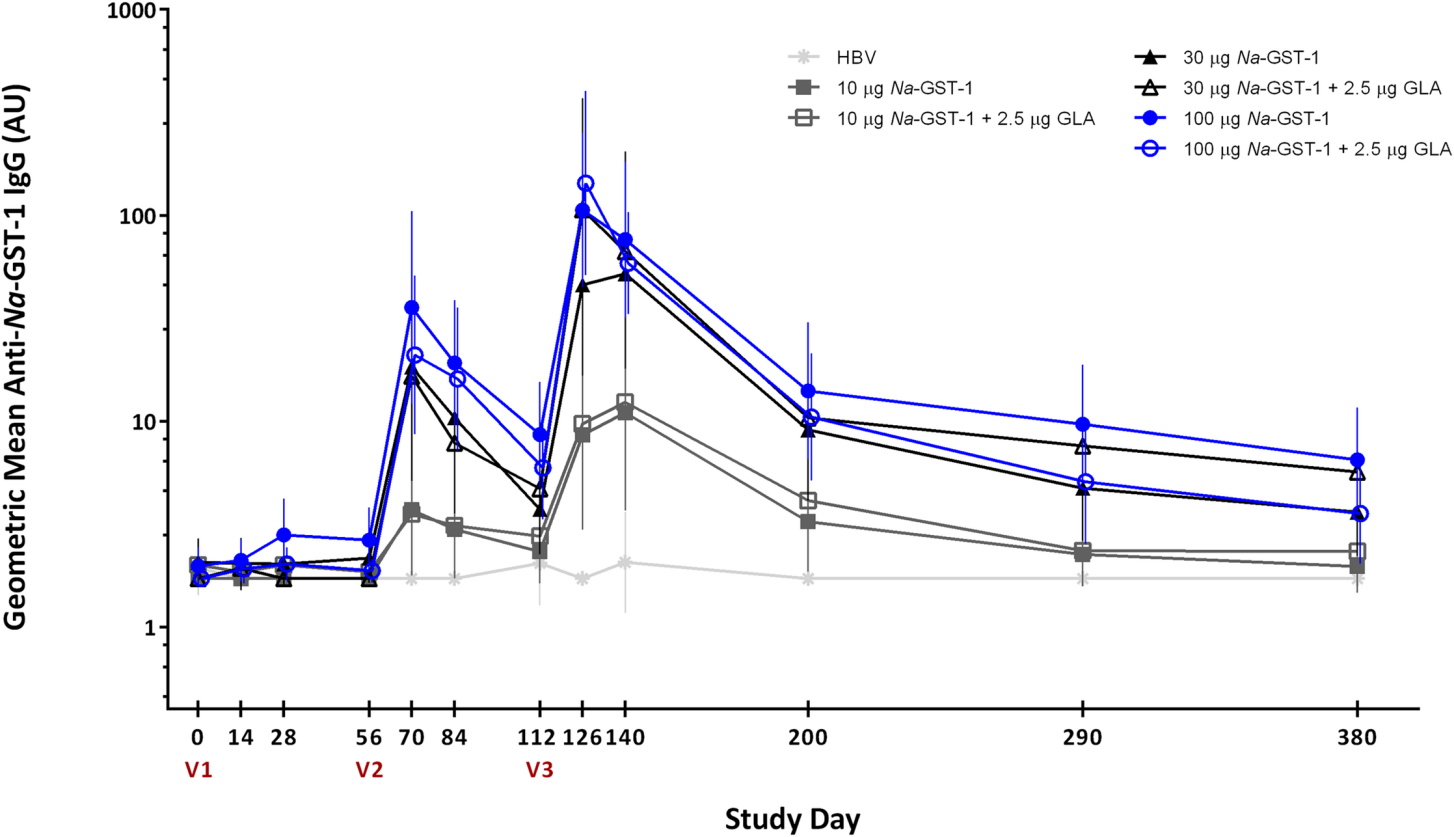
Geometric mean levels of IgG against recombinant *Na*-GST-1 in Brazilian participants immunized with *Na*-GST-1/Alhydrogel (administered with or without GLA-AF) or the hepatitis B vaccine, as measured by ELISA. Bars represent 95% confidence intervals. V1 = first vaccination; V2 = second vaccination; V3 = third vaccination; HBV = hepatitis B vaccine; AU = arbitrary units.

IgE antibodies to *Na*-GST-1 were not detected in any of the serum samples collected from individuals who were screened for the study in Brazil, in either Belo Horizonte or Americaninhas, and therefore no individuals were excluded from participation based on this eligibility criterion. In addition, anti-*Na*-GST-1 specific IgG antibody levels were below the LOQ in all but 14 participants in Brazil (n = 7) and the US (n = 7) prior to vaccination. Median IgG levels were below the LOQ at baseline in both studies (**Fig 3**and **Fig 4**).

### IgG responses to *Na*-GST-1 after vaccination

In both studies, anti-*Na*-GST-1 IgG levels in individuals vaccinated with recombinant *Na*-GST-1 increased with successive vaccinations and peaked on Study Day 126, 2 weeks following the third vaccination (**Fig 3**and **Fig 4**for the US and Brazilian studies, respectively). Geometric mean levels of *Na*-GST-1-specific IgG subsequently decreased, although they remained elevated above baseline for the duration of the trials. There was no significant change in IgG levels to *Na*-GST-1 in the volunteers vaccinated with the hepatitis B vaccine in Brazil.

In the study conducted in Brazil, vaccination with *Na*-GST-1/Alhydrogel administered with or without GLA-AF induced antigen-specific IgG antibodies in a dose-dependent fashion, with increasing antibody responses observed after successive vaccinations (**Fig 4**). Statistically significant differences in IgG levels were seen between the 100 μg *Na*-GST-1 dose and the hepatitis B vaccine controls after the second (median 28.74 *vs*. 1.72 AU, p<0.0001) and third (median 125.03 *vs*. 1.72 AU, p<0.0001) vaccinations; whereas, antigen-specific IgG levels were significantly higher than the hepatitis B vaccine group for the 30 μg *Na*-GST-1 dose after the third injection (53.25 *vs*. 1.72 AU, p<0.0001). Statistically significant differences in IgG levels were also seen at several time points between those vaccinated with the 30 μg or 100 μg doses of *Na*-GST-1 compared to those vaccinated with 10 μg *Na*-GST-1. However, no significant differences were seen at any time point between the levels of IgG in those who received 30 *vs*. 100 μg *Na*-GST-1. Furthermore, the addition of GLA-AF to the *Na*-GST-1/Alhydrogel formulations increased the median IgG response for each dose of *Na*-GST-1, when measured 2 weeks after the third vaccination (e.g., 199.79 *vs*. 125.03 AU for the 100 μg *Na*-GST-1 dose), although none of these differences were statistically significant. No differences were seen in the antibody responses observed in hookworm-naïve Brazilian study volunteers living in Belo Horizonte compared to hookworm-exposed study volunteers in Americaninhas for any of the doses or formulations of *Na*-GST-1.

In the US study, median anti-*Na*-GST-1 IgG antibody levels showed an increasing trend among dose groups although these differences were not statistically significant, most likely due to the small numbers of study participants in each group (**Fig 3**). On Study Day 126 (two weeks following the third vaccination), the median antibody level in the group that was vaccinated with 10 μg *Na*-GST-1 (regardless of co-administered dose of GLA-AF) was 12.0 AU, which was less than those vaccinated with 30 μg *Na*-GST-1 (18.9 AU); both of these doses resulted in IgG levels that were less than the median of the group vaccinated with the highest dose of 100 μg *Na*-GST-1 (88.4 AU). Comparing IgG responses based on GLA-AF co-administration status within *Na*-GST-1 dose groups also revealed no statistically significant differences at any time point between any of the groups. As shown in **Fig 3**, IgG levels increased progressively in study participants over the duration of the study. Similar to the Brazilian study, geometric mean antibody levels attained the highest detectable concentration 2 weeks following the third vaccination in all groups who received *Na*-GST-1.

### Comparison of anti-*Na*-GST-1 IgG responses between studies conducted in the US and Brazil

**Fig 5:**
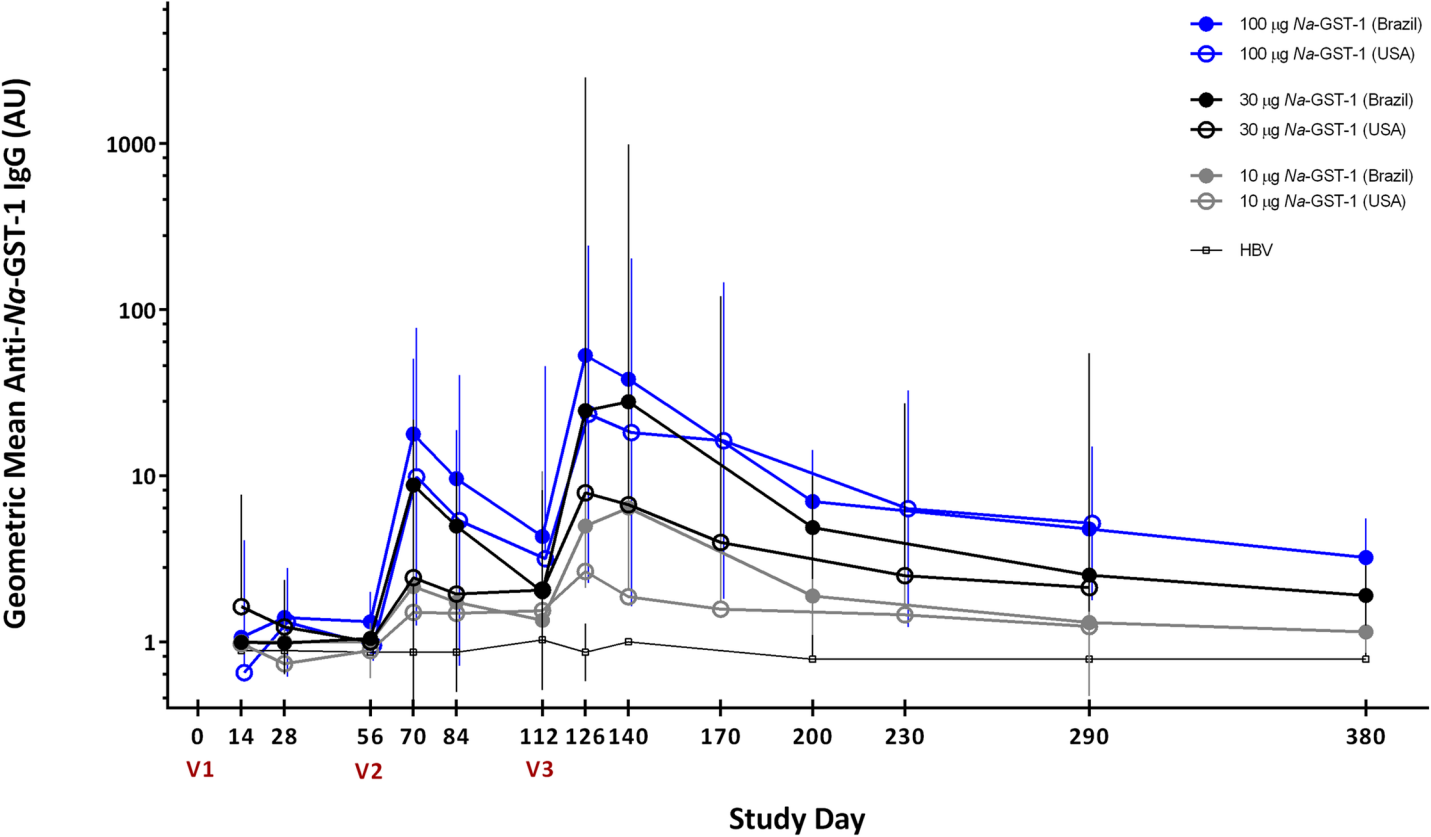
Comparison between US and Brazilian participants in levels of IgG against recombinant *Na*-GST-1 as measured by ELISA. Bars represent 95% confidence intervals. V1 = first vaccination; V2 = second vaccination; V3 = third vaccination; HBV = hepatitis B vaccine; AU = arbitrary units.

Overall, responses to vaccination with *Na*-GST-1/Alhydrogel were similar in the studies conducted in Brazil and in the US (**Fig 5**). Differences between anti-*Na*-GST-1 IgG antibody levels over time in the US and Brazilian volunteers were assessed by comparing changes in IgG levels for each individual to their baseline (i.e., Study Day 0) values. Although significant differences in changes in antibody level between all groups began to emerge 2 weeks after the second vaccination (Study Day 70) in both studies (**Table 3**), pair-wise comparisons indicated that these were largely due to increases in the Brazilian study volunteers vaccinated with *Na*-GST-1 compared to those who received the hepatitis B vaccine. Importantly, no significant changes in IgG levels from baseline were observed between Brazilian and US volunteers who received the same doses of *Na*-GST-1 on any of the study days compared. However, volunteers from both studies who were vaccinated with 100 μg of *Na*-GST-1 showed consistently greater median IgG increases from baseline than volunteers vaccinated with 30 μg or 10 μg of *Na*-GST-1.

**Table 3:** Comparison of changes in anti-*Na*-GST-1 IgG antibody levels from baseline between US and Brazilian participants vaccinated with *Na*-GST-1/Alhydrogel or the hepatitis B vaccine. Results for each study day are presented as the median absolute difference in IgG level (expressed in AU) compared to baseline (Study Day 0).

	10 μg *Na-*GST-1		30 μg *Na-*GST-1		100 μg *Na-*GST-1		HBV Vaccine	p-value*
	Brazil	US	Brazil	US	Brazil	US		
**Day 14**								
Sample size (n)	31	12	32	15	32	10	6	
Median (AU)	0	0	0	0	0	0	0	**0.0400**
**Day 28**								
Sample size (n)	32	11	32	16	31	11	6	
Median (AU)	0	0	0	0	0	0	0	**0.0261**
**Day 56**								
Sample size (n)	32	11	32	16	31	10	5	
Median (AU)	0	0	0	0	0	0	0	**0.0023**
**Day 70**								
Sample size (n)	31	11	30	15	31	10	5	
Median (AU)	0	0	15.59	6.55	15.72	15.62	0	**< .0001**
**Day 84**								
Sample size (n)	31	11	30	16	31	10	5	
Median (AU)	0	0	9.29	6.38	13.87	12.54	0	**< .0001**
**Day 112**								
Sample size (n)	31	11	29	15	31	9	5	
Median (AU)	0	0	2.2	6	4.99	7.46	0	**0.0020**
**Day 126**								
Sample size (n)	31	11	29	14	31	9	5	
Median (AU)	4.03	10.05	60.43	16.99	123.31	86.45	0	**< .0001**
**Day 140**								
Sample size (n)	30	11	29	14	31	9	4	
Median (AU)	6.63	3.42	73.74	19.47	58.55	41.4	0	**< .0001**

*Kruskal-Wallis test comparing all groups.

Since no significant differences were seen between changes in the anti-IgG-*Na*-GST-1 responses of individuals at the US and the two Brazilian sites, changes in anti-IgG-*Na*-GST-1 levels from baseline were combined and analyzed together. No differences in anti-*Na*-GST-1 IgG changes from baseline were observed between *Na*-GST-1 dose groups on Study Days 14 or 56. However, significant differences emerged on Study Day 70 (2 weeks after the second vaccination) and persisted through Day 140 (the last day shared by participants in both studies). Changes in IgG antibody levels from baseline between individuals vaccinated with either 30 μg or 100 μg *Na*-GST-1 were significantly different compared to those vaccinated with the hepatitis B vaccine on Study Days 70, 84, 126, and 140. This was not observed between recipients of the hepatitis B vaccine and recipients of 10 μg *Na*-GST-1; the only significant difference observed between participants in these two groups was 2 weeks after the third vaccination, on Study Day 126 (median change of 5.06 AU *vs*. 0 AU in the 10 μg *Na*-GST-1 *vs*. hepatitis B vaccine groups, p = 0.015).

Comparing participants who were vaccinated with different doses of *Na*-GST-1 also revealed statistically significant differences in the change in anti-*Na*-GST-1 IgG levels from baseline. Participants who received either 30 μg or 100 μg of *Na*-GST-1/Alhydrogel had consistently greater increases in IgG from baseline compared to those who were vaccinated with the 10 μg dose from Study Day 28 onward (individual comparisons; Kruskal Wallis test). However, no significantly different changes in IgG level from baseline were observed between recipients of the 30 μg and the 100 μg *Na*-GST-1 doses, except on Study Day 28 (p = 0.011), when a greater increase was seen in those vaccinated with 100 μg *Na*-GST-1.

### IgG subclass responses to *Na*-GST-1

**Fig 6:**
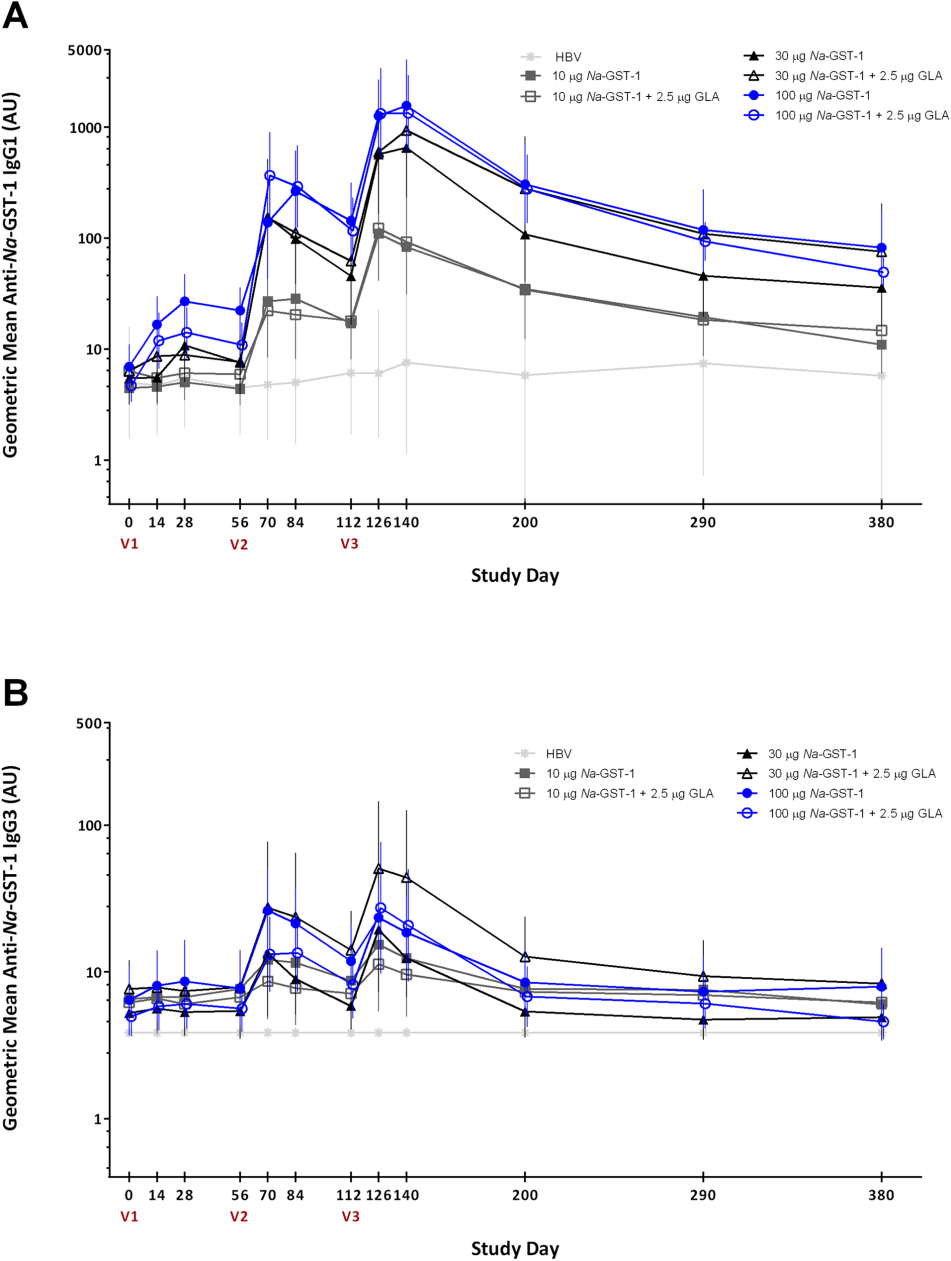
Levels of IgG1 (panel A) and IgG3 (panel B) against recombinant *Na*-GST-1 in Brazilian participants vaccinated with *Na*-GST-1/Alhydrogel (administered with or without GLA-AF) or the hepatitis B vaccine, as measured by ELISA. Bars represent 95% confidence intervals. V1 = first vaccination; V2 = second vaccination; V3 = third vaccination; HBV = hepatitis B vaccine; AU = arbitrary units.

In both the US and Brazil studies, the predominant IgG subclass induced against *Na*-GST-1 in participants receiving *Na*-GST-1/Alhydrogel was IgG1 (**Fig 6**). Lesser amounts of IgG3 were observed. Similar levels of IgG1 and IgG3 against *Na*-GST-1 were observed over time in participants in the Brazilian and the US studies. No differences in the distribution of the IgG1 and IgG3 subclasses against *Na*-GST-1 were seen when study participants received *Na*-GST-1/Alhydrogel with or without the co-administration of GLA-AF. No IgG2 or IgG4 antibodies against *Na*-GST-1 were detected in any participant, either in the US or Brazil.

## Discussion

This manuscript describes the results of two Phase 1 clinical trials of the first vaccine to target the adult blood feeding stage of a human nematode infection. The *Na*-GST-1/Alhydrogel vaccine for human hookworm disease caused by *N*. *americanus* was observed to be safe and well tolerated by both hookworm-naïve American and Brazilian adults, as well as hookworm-exposed Brazilians. Antigen-specific IgG responses, mostly of the IgG1 subclass, were induced in volunteers from these sites in a dose-dependent fashion with increasing levels of antibody observed after subsequent vaccinations.

Glutathione-S-transferases (GSTs) are ubiquitous proteins that serve a variety of functions. Vaccines–both human and veterinary–based on GST proteins have been developed to protect against several different organisms, including the trematode parasites *Schistosoma haematobium* and *Fasciola hepatica* [29, 30]. *Na*-GST-1 belongs to the Nu class of nematode GSTs that is characterized by reduced peroxidase activity relative to other classes of GSTs but an elevated capacity to bind free heme, an end-product of the parasite’s hemoglobin digestion pathway that is potentially toxic to the worm [14–18]. Recombinant *Na*-GST-1 has been selected as a promising human hookworm vaccine candidate after preclinical vaccination studies in animal models demonstrated reductions in worm burdens and fecal egg counts following challenge infection [14, 15, 19].

*Na*-GST-1 is the second hookworm vaccine to enter clinical development. The first hookworm vaccine to be tested in humans was the recombinant *Na*-ASP-2 (Ancylostoma Secreted Protein-2 of *N*. *americanus*) Hookworm Vaccine. *Na*-ASP-2 is an excretory/secretory product produced by infective *N*. *americanus* larvae upon penetration of human skin. A Phase 1 trial of *Na*-ASP-2 formulated on Alhydrogel conducted in healthy, hookworm-naïve adults living in the United States showed it to be safe, well tolerated and immunogenic [31]. However, upon testing the vaccine in adults who had previously been infected with hookworm in an endemic area of Brazil, several volunteers in the first (i.e., lowest dose) cohort developed generalized urticaria within 2 hours of administration of their first vaccination [26]. Subsequent investigations revealed that the volunteers who developed urticaria upon their first dose of *Na*-ASP-2 had elevated levels of pre-vaccination IgE to the vaccine antigen. Given that IgE antibodies to *Na*-ASP-2 were most likely induced by prior infection with *N*. *americanus*, we conducted a seroepidemiological survey in the same hookworm-endemic region of Brazil to investigate the prevalence and age distribution of anti-*Na*-ASP-2 IgE. This study revealed that even in young children, a significant proportion of individuals had detectable levels of IgE to *Na*-ASP-2 [26]. In addition, similar findings were demonstrated for other larval-stage *N*. *americanus* proteins that were being considered as vaccine candidates.

In contrast to the experience with *Na*-ASP-2, individuals living in a hookworm-endemic area who have been exposed to or infected with *N*. *americanus* were not expected to develop allergic responses upon vaccination with *Na*-GST-1. Extensive studies were conducted to test for sensitization to the *Na*-GST-1 protein induced by natural hookworm infection. Over 1000 individuals aged 1 to 85 years from the hookworm-endemic area surrounding Americaninhas were tested for IgE antibodies to *Na*-GST-1 using an indirect ELISA. In addition, a subset of these samples stratified by age and hookworm infection status (n = 179) underwent confirmatory testing using a custom ImmunoCAP assay, which demonstrated that none of the samples had *Na*-GST-1 IgE values above the clinical threshold of 0.35 kU_A_/L (**S1 Fig**). Furthermore, in the Phase 1 trial of *Na*-GST-1/Alhydrogel conducted in Brazil that is reported herein, none of the adult volunteers who were screened for the study had detectable IgE antibodies to *Na*-GST-1, further adding to the body of evidence that this antigen does not induce sensitization during natural hookworm infection. Therefore, continued clinical development of this product should be safe from the standpoint of allergenic potential.

In endemic areas, children become infected with hookworm early in life and may remain infected into adulthood [32, 33], with no evidence that infection results in protection against subsequent re-infection [34, 35]. Therefore, the nature of the protective immune response that an effective hookworm vaccine will have to induce is currently unknown, but will most likely be different than the immune response induced by natural hookworm infection. Accordingly, the proposed mechanism of action of the *Na*-GST-1/Alhydrogel vaccine is not to reproduce the immune response in individuals living in areas with hookworm transmission. Rather, it is to induce IgG antibodies, especially IgG1 antibodies, that could be ingested by developing hookworms after they reach the host intestinal tract to block the detoxifying action of parasite *Na*-GST-1, thereby leaving free heme to accumulate and damage the worm. Analogous mechanisms of action have been proposed for malaria transmission blocking vaccines [36] and the previously marketed Lymerix vaccine for Lyme disease [37], in which vaccine-induced antibodies are ingested by blood-feeding vectors and subsequently interfere with parasite development.

Given this proposed mechanism of action, the aim of formulating the *Na*-GST-1 antigen has been to maximize the levels IgG, especially IgG1 antibodies, against *Na*-GST-1 in vaccinated individuals. For this reason, in the clinical trials reported in this manuscript, the *Na*-GST-1/Alhydrogel vaccine was tested in combination with the GLA-AF immunostimulant, in the hopes that it might increase antibody responses to the antigen. GLA-AF contains a synthetic monophosphoryl lipid A (MPL) molecule that has TLR4 agonist activity. MPL is itself derived from the lipopolysaccharide of *Salmonella minnesota*, a natural TLR4 agonist that is pyrogenic and can induce toxic shock [38]. Lipopolysaccharide, and more specifically, its lipid A component, has long been known for its strong adjuvant effects; however, its high toxicity has precluded its use in a vaccine formulation. Ribi et al showed that the monophosphorylated form of lipid A retains its adjuvant function and almost completely loses its endotoxin effects [38]. Unfortunately, in the two trials reported herein, the addition of GLA-AF to the Alhydrogel formulation of *Na*-GST-1 did not result in any significant increase in IgG responses in either Brazilian or American healthy adults.

Reassuringly, no differences in the safety or the humoral immune responses to the recombinant protein were observed between volunteers vaccinated with *Na*-GST-1/Alhydrogel in hookworm-naïve volunteers in the US or Brazil compared to those living in a region of Brazil where hookworm transmission is high. This differs from other vaccines, where differences in safety, immunogenicity, and efficacy have been observed between those living in industrialized countries and those in less developed areas of the world [39, 40].

In conclusion, the *Na*-GST-1/Alhydrogel vaccine was found to be safe and immunogenic in both hookworm-naïve and hookworm-exposed adults. It induced antigen-specific IgG, especially of the IgG1 subclass, antibodies in a dose-dependent fashion. Although the addition of the GLA-AF immunostimulant did not significantly increase antibody responses to *Na*-GST-1, the level of antibody response needed for protection still needs to be determined in larger clinical trials. Given the results presented herein, further studies in children are planned to eventually test the efficacy of this vaccine against development of hookworm disease.

## Supporting information

S1 Fig Anti-*Na*-GST-1 IgE levels in a subset of adults and children (n = 179) living in a hookworm-endemic area of Brazil. IgE levels (kU_A_/L) were measured by custom ImmunoCAP.
(TIF)

S1 Table Baseline characteristics of study participants in Brazil clinical trial. (PDF)

S2 Table Baseline characteristics of study participants in US clinical trial. (PDF)

S1 Supporting Information CONSORT checklist. (PDF)

S2 Supporting Information Brazil clinical trial protocol. (PDF)

S3 Supporting Information US clinical trial protocol. (PDF)
