# Correction: Safety and immunogenicity of the *Na*-GST-1 hookworm vaccine in Brazilian and American adults

**DOI:** 10.1371/journal.pntd.0008670

**Published:** 2020-08-17

**Authors:** David J. Diemert, Janaína Freire, Vanderson Valente, Carlo Geraldo Fraga, Frederico Talles, Shannon Grahek, Doreen Campbell, Amar Jariwala, Maria Victoria Periago, Martin Enk, Maria Flávia Gazzinelli, Maria Elena Bottazzi, Robert Hamilton, Jill Brelsford, Anna Yakovleva, Guangzhao Li, Jin Peng, Rodrigo Correa-Oliveira, Peter Hotez, Jeffrey Bethony

The fourth author’s name is spelled incorrectly. The correct name is Carlo Geraldo Fraga.

## References

[1] Diemert DJ, Freire J, Valente V, Fraga CG, Talles F, Grahek S, et al (2017) Safety and immunogenicity of the *Na*-GST-1 hookworm vaccine in Brazilian and American adults. PLoS Negl Trop Dis 11(5): e0005574 10.1371/journal.pntd.0005574 28464026 PMC5441635

