# Supplementary figures and images for: Safety and immunogenicity of the Na-GST-1 hookworm vaccine in Brazilian and American adults
Source: PLoS Negl Trop Dis. 2017 May 2;11(5):e0005574. doi: 10.1371/journal.pntd.0005574 (PMC5441635; doi:10.1371/journal.pntd.0005574)

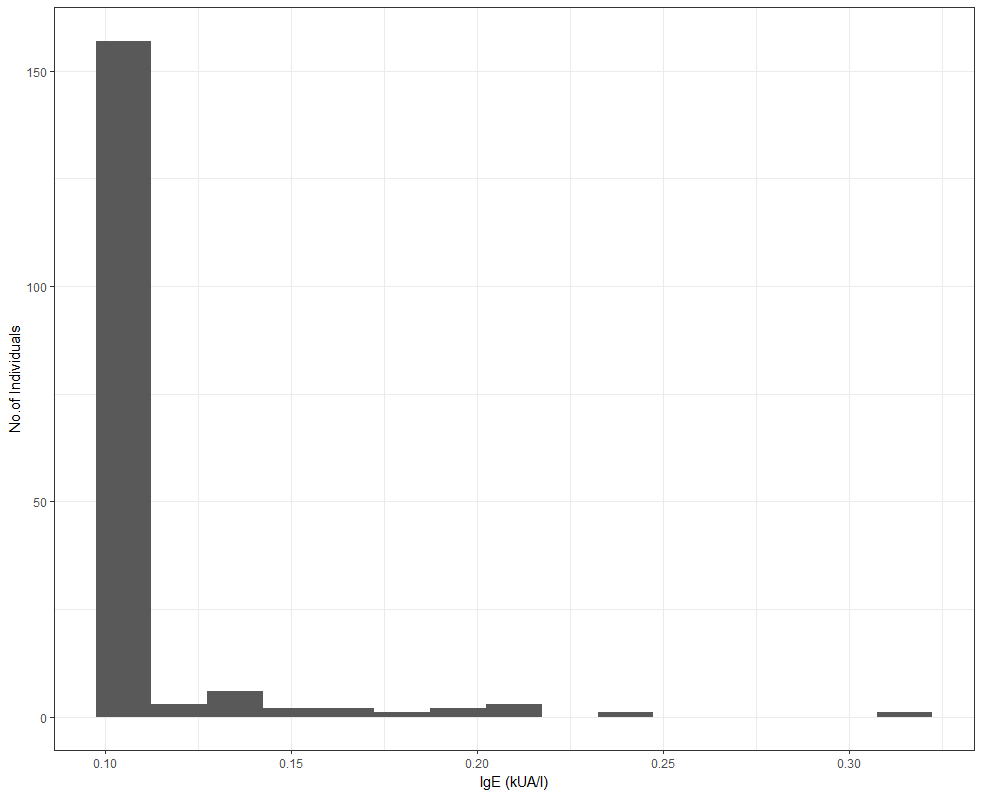

Supplement: S1 Fig — IgE levels (kUA/L) were measured by custom ImmunoCAP. (TIF) [file pntd.0005574.s001.tif]
