# Supplementary material for: Safety and immunogenicity of the Na-GST-1 hookworm vaccine in Brazilian and American adults
Source: PLoS Negl Trop Dis. 2017 May 2;11(5):e0005574. doi: 10.1371/journal.pntd.0005574 (PMC5441635; doi:10.1371/journal.pntd.0005574)
Supplement: S1 Table — (PDF) [file pntd.0005574.s002.pdf]

	**10 µg *Na*-GST-1**				**30 µg *Na*-GST-1**				**100 µg *Na*-GST-1**				**HBV vaccine**		**Overall**	
	**+ 0 µg GLA-AF**		**+ 2.5 µg GLA-AF**		**+ 0 µg GLA-AF**		**+ 2.5 µg GLA-AF**		**+ 0 µg GLA-AF**		**+ 2.5 µg GLA-AF**					
**Sample size (n)**	n=16		n=16		n=16		n=16		n=16		n=16		n=6		N=102	
	**Mean**	**SD**	**Mean**	**SD**	**Mean**	**SD**	**Mean**	**SD**	**Mean**	**SD**	**Mean**	**SD**	**Mean**	**SD**	**Mean**	**SD**
Age* (years)	30.4	8.2	27.5	9.1	28.3	7.7	25.1	5.4	25.6	7.4	29.4	7.3	29.2	8.6	27.8	7.7
Gender** (% male)	50.0		68.8		43.8		37.5		43.8		50.0		33.3		48.0	
BMI* (kg/m^2)	24.8	3.2	24.2	3.1	25.0	3.1	22.3	3.5	22.0	2.6	23.1	4.2	24.6	3.1	23.6	3.4

* Presented as median (interquartile range).
** Presented as number (percent) of male participants.
