# Supplementary material for: Safety and immunogenicity of the Na-GST-1 hookworm vaccine in Brazilian and American adults
Source: PLoS Negl Trop Dis. 2017 May 2;11(5):e0005574. doi: 10.1371/journal.pntd.0005574 (PMC5441635; doi:10.1371/journal.pntd.0005574)
Supplement: S2 Table — (PDF) [file pntd.0005574.s003.pdf]

	10 µg *Na*-GST-1				30 µg *Na*-GST-1						100 µg *Na*-GST-1				Overall	
	**+ 0 µg GLA-AF**		**+ 1 µg GLA-AF**		**+ 0 µg GLA-AF**		**+ 1 µg GLA-AF**		**+ 5 µg GLA-AF**		**+ 0 µg GLA-AF**		**+ 5 µg GLA-AF**			
**Sample size (n)**	n=4		n=8		n=4		n=8		n=4		n=4		n=8		N=40	
	**Mean**	**SD**	**Mean**	**SD**	**Mean**	**SD**	**Mean**	**SD**	**Mean**	**SD**	**Mean**	**SD**	**Mean**	**SD**	**Mean**	**SD**
Age* (years)	32.5	9.0	37.0	12.5	41.5	6.5	35.5	11.0	27.0	9.5	18.0	10.5	34.5	9.0	34.5	11.5
Gender** (% male)	75.0		62.5		75.0		50.0		75.0		75.0		37.5		60.0	
BMI* (kg/m^2)	31.4	3.5	30.9	7.8	29.7	2.9	31.8	8.0	31.9	6.3	27.0	5.1	33.7	9.0	31.3	6.8

* Presented as median (interquartile range).
** Presented as number (percent) of male participants.
