# Supplementary material for: Safety and immunogenicity of the Na-GST-1 hookworm vaccine in Brazilian and American adults
Source: PLoS Negl Trop Dis. 2017 May 2;11(5):e0005574. doi: 10.1371/journal.pntd.0005574 (PMC5441635; doi:10.1371/journal.pntd.0005574)
Supplement: S2 Supporting Information — (PDF) [file pntd.0005574.s005.pdf]

PHASE 1 STUDY OF THE SAFETY AND IMMUNOGENICITY OF *NA*-GST-1/ALHYDROGEL® WITH OR WITHOUT GLA-AF IN BRAZILIAN ADULTS

Version:	#8 (13 June 2013)
Protocol Number:	SVI-10-01
FDA (US) IND Number:	14616
GWUMC IRB Number:	011139
CPqRR IRB Number:	30/2010
CONEP (Brazil) Number:	16345

Sponsored by:
Albert B. Sabin Vaccine Institute
2000 Pennsylvania Avenue, Suite 7100
Washington, DC 20006
Tel: 202-842-5025 Fax: 202-842-7689

TEAM ROSTER

Principal Investigator:

David Diemert, MD
Department of Microbiology, Immunology and Tropical Medicine (MITM)
George Washington University
Washington, DC, USA
Tel: 202-994-2909
Email: ddiemert@gwu.edu

Clinical Investigators:

Vanderson Valente, MD
Frederico Talles, MD
Janaina Freire, MD
Carlo Geraldo Fraga de Castro, MD
Centro de Pesquisas René Rachou (CPqRR)
FIOCRUZ em Minas Gerais
Belo Horizonte, Brazil

Scientific Investigators:

Jeffrey Bethony, PhD
MITM
George Washington University
Washington, DC, USA

Rodrigo Correa de Oliveira, PhD
CPqRR, FIOCRUZ em Minas Gerais
Belo Horizonte, Brazil

Regulatory Affairs Contact:

Marva Loblack, MS, MBA
Sabin Vaccine Institute
Washington, DC, USA

Medical Monitor – Part 1:

Lilian Martins Oliveira Diniz, MD
Universidade Federal de Minas Gerais
Santa Efigênia, Belo Horizonte, MG, Brasil

Medical Monitor – Part 2:

Daniel Moreira Pinto, MD
Coordenação de Atenão Básica
Téofilo Otoni, MG, Brasil

Participating Sites

**Clinical Trial Sites:**	**Centro de Pesquisas René Rachou - FIOCRUZ**<br>Av. Augusto de Lima 1715, Belo Horizonte,<br>Minas Gerais, Brazil<br>Tel: +55 (31) 3349 7715<br>Fax: +55 (31) 3295 3115
	**Americaninhas Vaccine Center**<br>Americaninhas, Municipality of Novo Oriente<br>Minas Gerais, Brazil<br>Tel: +55 (33) 3532-7066

Participating Laboratories

**Clinical Laboratories:**	**Laboratório Humberto Abrão**<br>Rua Marília de Dirceu, 219<br>CEP 30170-090<br>Lourdes<br>Belo Horizonte, Minas Gerais<br>Brazil
	**Laboratório Carlos Chagas**<br>Rua Sete de Setembro, 2000 – Centro<br>CEP 35010 - 172<br>Governador Valadares<br>Minas Gerais, Brazil<br>Tel: +55 (33) 3271 2525
**Immunology Laboratory:**	**Laboratório de Imunologia Celular e Molecular**<br>Centro de Pesquisas René Rachou - FIOCRUZ<br>Av. Augusto de Lima 1715, Belo Horizonte,<br>Minas Gerais, Brazil<br>Tel: +55 (31) 3349 7715<br>Fax: +55 (31) 3295 3115

This document is a confidential communication of the Sabin Vaccine Institute. Acceptance of this document constitutes agreement by the recipient that no unpublished information contained herein will be published or disclosed without Sabin Vaccine Institute's prior written approval, except that this document may be disclosed to appropriate Institutional Review Boards under the condition that they are required to keep it confidential.

Table of Abbreviations

AE	Adverse Event
AESI	Adverse Event of Special Interest
CPqRR	Centro de Pesquisas René Rachou
CFR	Code of Federal Regulations (USA)
cGCP	Current Good Clinical Practices
cGMP	Current Good Manufacturing Practices
CRF	Case Report Form
ELISA	Enzyme linked immunosorbent assay
FDA	Food and Drug Administration (USA)
FIOCRUZ	Foundation Oswaldo Cruz
GLA-AF	Gluco-Pyranosylphospho-Lipid A Aqueous Formulation
GST-1	Glutathione S-Transferase
HBsAg	Hepatitis B surface antigen
HBV	Hepatitis B virus
hCG	Human choriogonadotropin
HCV	Hepatitis C virus
HHVI	Human Hookworm Vaccine Initiative
HIV	Human Immunodeficiency Virus
ICH	International Conference on Harmonization
IM	Intramuscular
INR	International Normalized Ratio
IRB	Institutional Review Board
MedDRA®	Medical Dictionary of Regulatory Activities
Na	*Necator americanus*
PBMC	Peripheral Blood Mononuclear Cell
PT	Prothrombin time
PTT	Partial thromboplastin time
SAE	Serious Adverse Event
SVI	Sabin Vaccine Institute
US	United States
WBC	White blood cell
WHO	World Health Organization

Table of Contents

TEAM ROSTER 2
Table of Abbreviations 4
Protocol Summary 9
1 Introduction 12
1.1 Background 12
1.2 Rationale for Developing a Hookworm Vaccine 13
1.5 *Na*-GST-1 Vaccines 20
1.6 Preclinical Toxicity Study of *Na*-GST-1 Hookworm Vaccines 20
1.7 Immunogenicity Studies with *Na*-GST-1 20
1.8 *In vitro* Tissue Cross-Reactivity Studies 22
1.9 Clinical Experience with *Na*-GST-1 23
1.10 Clinical Experience with Aluminum-Based Adjuvants 23
1.11 Clinical Experience with GLA-AF 23
1.12 Clinical Development Plan 24
1.13 Comparator Vaccine 24
1.13.1 Rationale for use of a comparator vaccine 24
1.13.2 Rationale for use of the Hepatitis B vaccine as a comparator 25
2 Objectives 25
2.1 Primary Objective 25
2.2 Secondary Objectives 25
3 Study Sites 25
4 Study Design 27
4.1 Overall Design 27
4.2 Sample Size and Estimated Duration of Study 31
4.3 Group Allocation 32
4.4 Blinding 32
5 Selection of Participants 34
5.1 Inclusion Criteria 34
5.2 Exclusion Criteria 34
6 Vaccine Preparation 35
6.1 Supplies 35
6.3 Preparation of Vaccine Doses 36
6.4 Vaccine Storage 36
6.5 Vaccine Accountability 36
6.6 Disposition of Used/Unused Supplies 37
7 Study Procedures 37
7.1 Community Consent for Part II of the Study 37
7.2 Individual Recruitment and Informed Consent 37
7.3 Randomization Process for Part II of the Study 39
7.4 Enrollment 39
7.5 Immunization Procedure 39
7.6 Clinical Monitoring and Evaluation 39
7.7 Laboratory Testing 43
7.8 Immunologic Testing 43
7.8.1 Anti-Na-GST-1 Antibody Assays 43
7.8.2 Western Blots 44
7.8.3 Total IgE 44
7.8.4 Cellular Immunology Assays 44
7.8.5 Storage of Participant Specimens 44

7.9 Stool Examination 44
8 Adverse Events Monitoring and Reporting 45
8.1 Definitions 45
8.1.1 Adverse Event 45
8.1.2 Serious Adverse Event (SAE) 45
8.1.3 Adverse Events of Special Interest (AESIs) 45
8.2 Assessment of Adverse Events 46
8.2.1 Identification of AEs 46
8.2.2 Determination of Severity 46
8.2.3 Association with Receipt of the Study Vaccine 49
8.3 Adverse Event Reporting 49
8.4 Adverse Event Monitoring 50
8.4.1 Medical Monitor 50
8.4.2 Safety Monitoring Committee (SMC) 50
8.5 Criteria for Suspension of Further Injections 51
8.6 Treatments That Could Potentially Interfere with Vaccine-Induced Immunity .. 52
8.7 Criteria for Suspension of Injections 52
8.8 Criteria for Deferral of Injections 52
8.9 Criteria for Withdrawal of an Individual Participant from the Study 53
8.10 Breaking the Study Blind 53
9 Data Collection and Monitoring 54
9.1 Source Documentation and Case Report Forms 54
9.2 Study Documentation 54
9.2.1 Study Reports 54
9.3 Retention of Records 54
9.4 Protocol Revisions 55
9.5 Clinical Investigator's Brochure 55
9.6 Study Monitoring 55
10 Statistical Considerations 55
10.1 General Design 55
10.1.1 Description of the Statistical Methods to Be Employed 55
10.2 Sample Size 58
11 Protection of Human Subjects 58
11.1 Institutional Review Boards 58
11.2 Informed Consent 58
11.3 Risks 59
11.3.1 Venipuncture 59
11.3.2 Immunization with *Na*-GST-1/Alhydrogel® 59
11.3.3 Immunization with *Na*-GST-1/Alhydrogel® plus GLA-AF 60
11.3.4 Immunization with Butang® 60
11.4 Precautions taken to Minimize Risks 60
11.5 Benefits 60
11.6 Confidentiality 61
11.7 Compensation 61
11.8 Publication of Study Results 61
12 References 62

Appendix A – Informed Consent Comprehension Questionnaire 65
Appendix B – Schedule of Visits 66
Appendix C – Toxicity Table for Grading Laboratory Adverse Events 67
Appendix D – Toxicity Table for Grading Unsolicited Systemic Adverse Events 69

List of Figures and Tables

Figure 1: Vaccination of Brazilian Adults with Previous Hookworm Infection with 10 μg *Na*-ASP-2/Alhydrogel® induced immediate-type urticarial reactions. 14
Figure 2: Anti-*Na*-ASP-2 IgE antibody levels in residents of a hookworm-endemic region of northeastern Minas Gerais state, Brazil. 15
Figure 3: Comparison of GST dimers from parasitic nematodes, *N. americanus* (*Na*) and *Heligomosoides polygyrus* (*Hpol*) of the Nu class and comparison with a GST of the Sigma class. 16
Figure 4: Geometric mean titers of the IgG1 and IgG2 antibody responses of vaccinated dogs against r*Ac*-GST-1 formulated with GlaxoSmithKline's AS03 adjvant. 17
Figure 5: Anti-*Na*-GST-1 IgE levels in (A) adults and children and (B) young children aged 1-10 years (n=128) living in a hookworm-endemic area of Brazil. 18
Figure 6. Anti-*Na*-GST-1 IgE levels in a subset of adults and children (n=179) living in a hookworm-endemic area of Brazil. 19
Figure 7. Anti-*Na*-GST-1 IgG levels (arbitrary units) in Sprague-Dawley rats vaccinated with recombinant *Na*-GST-1. 21
Figure 8. Geometric mean anti-*Na*-GST-1 IgG antibody units 2 weeks after the 2nd Immunization of BALB/c mice with *Na*-GST-1/Alhydrogel® with or without co-administration of CPG 10104 and CPG 10105. 22

Table 1: Study Design 29
Table 2: Part I Vaccination Schedule 30
Table 3: Part II Dose Escalation Schedule 31
Table 4: Assessment of Adverse Event Severity 48

PRINCIPAL INVESTIGATOR'S STATEMENT:

I, the undersigned, have reviewed this protocol, including Appendices, and will conduct the clinical study as described and will adhere to the principles of the ICH/cGCP as well as all applicable regulatory requirements. I have read and understood the contents of the Investigator's Brochure provided by the Human Hookworm Vaccine Initiative (HHVI) of the Albert B. Sabin Vaccine Institute.

David Diemert, MD

____
Signature

____
Date

Protocol Summary

**Title**	**Phase 1 Study of the Safety and Immunogenicity of *Na*-GST-1/Alhydrogel® with or without GLA-AF in Brazilian Adults**
**Study Population**	Healthy male and non-pregnant female volunteers aged 18-45 years, inclusive.
**Rationale**	*Na*-GST-1 is a protein expressed during the adult stage of the *Necator americanus* hookworm life cycle that is thought to play a role in the parasite's degradation of host hemoglobin for use as an energy source. Vaccination with recombinant GST-1 has protected dogs and hamsters from infection in challenge studies. This study will evaluate the safety and immunogenicity of two formulations of *Na*-GST-1 first in hookworm-naïve individuals and then in adults living in an area of endemic hookworm infection. The two formulations consist of (a) *Na*-GST-1 adsorbed to Alhydrogel® and (b) *Na*-GST-1 adsorbed to Alhydrogel® and co-administered with the adjuvant GLA-AF.
**Study Design**	**Two part study:**

Part I: Open-label phase 1 trial in hookworm-naïve adults.

- Study site: Centro de Pesquisas René Rachou, Belo Horizonte, Brazil
- Number of participants: 36 in 6 groups of 6. The first, third, and fifth groups will receive *Na*-GST-1/Alhydrogel® and the second, fourth, and sixth will receive *Na*-GST-1/Alhydrogel® **plus** GLA-AF. Cohorts will be enrolled consecutively, with the first and second receiving 10 μg *Na*-GST-1, the third and fourth 30 μg *Na*-GST-1, and the fifth and sixth 100 μg *Na*-GST-1.

Part II: Double-blind, randomized, controlled dose-escalation phase 1 clinical trial in hookworm exposed adults.

- Study site: Americaninhas, Minas Gerais, Brazil.
- Number of participants: 66 in 6 groups, randomized to receive either *Na*-GST-1/Alhydrogel®, *Na*-GST-1/Alhydrogel®/GLA-AF, or Butang® hepatitis B vaccine.

- Immunization schedule: Study days 0, 56 and 112.
- Route: IM in the deltoid muscle.
- Doses of *Na*-GST-1 to be tested: 10, 30 and 100 μg
- Doses of Alhydrogel®: 80, 240 and 800 μg for the 10, 30 and 100 μg doses of *Na*-GST-1.
- Dose of GLA-AF: 2.5 μg.

- Study duration: 24 months; each participant will be followed for a total of 16 months.

Immunization Schedule

Cohort	Total Participants	Immunization Schedule		
		Day 0	Day 56	Day 112
**PART I**				
1	6	A(n=6)	A(n=6)	A(n=6)
2	6	D(n=6)	D(n=6)	D(n=6)
3	6	B(n=6)	B(n=6)	B(n=6)
4	6	E(n=6)	E(n=6)	E(n=6)
5	6	C(n=6)	C(n=6)	C(n=6)
6	6	F(n=6)	F(n=6)	F(n=6)
**PART II**				
7	10	A(n=8) + G(n=2)	A(n=8) + G(n=2)	A(n=8) + G(n=2)
8	12	A(n=2) + D(n=10)	A(n=2) + D(n=10)	A(n=2) + D(n=10)
9	10	B(n=8) + G(n=2)	B(n=8) + G(n=2)	B(n=8) + G(n=2)
10	12	B(n=2) + E(n=10)	B(n=2) + E(n=10)	B(n=2) + E(n=10)
11	10	C(n=8) + G(n=2)	C(n=8) + G(n=2)	C(n=8) + G(n=2)
12	12	C(n=2) + F(n=10)	C(n=2) + F(n=10)	C(n=2) + F(n=10)
Total	102	A: 10 µg *Na*-GST-1/Alhydrogel<br>B: 30 µg *Na*-GST-1/Alhydrogel<br>C: 100 µg *Na*-GST-1/Alhydrogel<br>D: 10 µg *Na*-GST-1/Alhydrogel/GLA-AF<br>E: 30 µg *Na*-GST-1/Alhydrogel/GLA-AF<br>F: 100 µg *Na*-GST-1/Alhydrogel/GLA-AF<br>G: Butang® hepatitis B vaccine		

Objectives

Primary

1. To estimate the frequency of vaccine-related adverse events, graded by severity, for each dose and formulation of *Na*-GST-1.

Secondary

1. To determine the dose and formulation of *Na*-GST-1 that generates the highest IgG antibody response at Day 126, as determined by an indirect enzyme-linked immunosorbent assay (ELISA).
2. To assess and compare the duration of antibody response to *Na*-GST-1.
3. To perform exploratory studies of the cellular immune responses to the *Na*-GST-1 antigen both before and after immunization.

Product Descriptions

The *Na*-GST-1 formulations to be studied contain the recombinant *Na*-GST-1 protein expressed by *Pichia pastoris*. Purified *Na*-GST-1 was subsequently adsorbed onto aluminum hydroxide gel (Alhydrogel®) and suspended in a solution containing 10% glucose and 10 mM imidazole. The final concentration of *Na*-GST-1 in the drug product is 0.1 mg/ml whereas that of Alhydrogel® is 0.8 mg/ml. Different doses of *Na*-GST-1 will be delivered by injecting different volumes of the 0.1 mg/ml *Na*-GST-1 preparation.

The *Na*-GST-1/Alhydrogel®/GLA-AF formulation will be prepared immediately prior to vaccination by adding an appropriate volume of GLA-AF (Gluco-Pyranosylphospho-Lipid A Aqueous Formulation) to *Na*-GST-1/Alhydrogel® and withdrawing an appropriate volume to administer the desired amount of *Na*-GST-1.

The comparator vaccine is the Butang® hepatitis B vaccine (Butantan, Brazil), containing recombinant hepatitis B surface protein expressed by *Pichia angusta*, adsorbed to aluminum hydroxide.

1 INTRODUCTION

1.1 Background

There is an urgent need for new tools to control human hookworm infection and to reduce its burden of disease in developing countries. This is especially true for children and women of reproductive age who represent populations that are highly vulnerable to the effects of hookworm disease. Up to 65,000 deaths annually have been attributed to human hookworm infection.[1] However, the mortality figures pale in comparison to global disease burden estimates that suggest that hookworm may account for the loss of up to 22 million Disability Adjusted Life Years annually.[2] With the exception of malaria, hookworm is the most important parasitic disease of humans.

Human hookworm infection is a soil-transmitted helminth infection caused by the nematode parasites *Necator americanus* and *Ancylostoma duodenale*. It is one of the most common chronic infections of humans, afflicting up to 740 million people in the developing nations of the tropics.[2] The largest number of cases occurs in impoverished rural areas of sub-Saharan Africa, Southeast Asia, China, and the tropical regions of the Americas. Approximately 3.2 billion people are at risk for hookworm infection in these areas. *N. americanus* is the most common hookworm worldwide, whereas *A. duodenale* is more geographically restricted.[3]

Hookworm transmission occurs when skin comes into contact with infective third-stage larvae (L3) in fecally-contaminated soil. The L3 have the ability to penetrate the skin, usually of the hands, feet, arms, buttocks and legs. The L3 invade human tissues and enter the gastrointestinal tract where they molt to the adult stage approximately 5-9 weeks following initial host entry. Adult hookworms are approximately 1 cm long parasites that cause host injury by attaching to the mucosa and submucosa of the small intestine to produce intestinal blood loss. There is a direct relationship between hookworm intensity (as determined by fecal egg counts) and host blood loss; typically the presence of between 40 and 160 adult hookworms in the intestine results in blood loss sufficient to cause anemia and malnutrition. The term "hookworm disease" refers primarily to the iron deficiency anemia and protein losses that occur in moderate and heavy infections.[4] When host iron stores become depleted, there is a direct correlation between hookworm intensity and reduced host hemoglobin, serum ferritin, and protoporphyrin. Because of their low iron stores, children and women of reproductive age are the populations considered the most vulnerable to hookworm-associated blood loss.[4-11]

In children, chronic hookworm infection and the resultant iron deficiency anemia have been shown to impair physical and intellectual development.[3, 12, 13] Preschool children are particularly vulnerable to the effects of hookworm anemia and disease.[8] In addition to its health impact on children, hookworm infection also affects adults. Unlike other soil-transmitted helminth infections, such as ascariasis and trichuriasis, in which the highest intensity infections occur almost exclusively in school-aged children, it has been shown that high-intensity hookworm infections may also occur in adult populations.[14-16]

The primary approach to hookworm control worldwide has been the frequent and periodic mass administration of benzimidazole anthelminthics to school-aged children living in high-prevalence areas. In 2001, the World Health Assembly adopted Resolution 54.19 which urges

member states to provide regular anthelminthic treatment to high-risk groups with the target of regular treatment of at least 75% of all at-risk school-aged children. However, the cure rates for a single dose of a benzimidazole varies with rates as low as 61% (400 mg) and 67% (800 mg) for albendazole and 19% (single dose) and 45% (repeated dose) for mebendazole being reported.[17-20] These concerns have prompted interest in developing alternative tools for hookworm control.[3, 21, 22] Vaccination to prevent the anemia associated with moderate and heavy intensity hookworm infection would alleviate the public health deficiencies of drug treatment alone.

1.2 Rationale for Developing a Hookworm Vaccine

The feasibility of developing a hookworm vaccine is based on the previous success of using live, irradiated hookworm larvae (L3 stage) as a vaccine for canine hookworm infection. This provided the experimental basis for the commercial development of a canine hookworm vaccine, which was marketed in the United States during the early 1970s. However, it is not realistic to develop a live L3 vaccine for humans due to multiple reasons including high production costs, challenging storage requirements (8-10°C), a short shelf-life, and a lack of sterilizing immunity.

Alternatively, the strategy being pursued is to identify key hookworm proteins to which protective immune responses are directed in the animal models for this infection (namely the canine model) and to produce these as recombinant proteins that could then be used as vaccine antigens. This effort has focused primarily on identifying antigens expressed by the invading larvae (L3). In addition, a separate strategy has been to identify targets of the adult stage of the hookworm lifecycle; since hookworms attach onto the intestinal lumen and ingest host blood, antibodies could also be ingested that if directed against key hookworm proteins, would interfere with their function, ultimately resulting in the death or reduced fecundity of the worm.

As originally conceived, an eventual hookworm vaccine would ideally consist of a combination of one or more proteins targeting the larval stage of the hookworm life cycle together with one or more antigens targeting the adult stage. Such a vaccine could therefore potentially interfere with both distinct stages of the hookworm life cycle.

1.3 Prior Clinical Experience with Hookworm Vaccines

The first hookworm vaccine to be tested in humans was the *Na*-ASP-2 (Ancylostoma Secreted Protein-2 of *N. americanus*) Hookworm Vaccine, consisting of recombinant *Na*-ASP-2 expressed in *Pichia pastoris* and adsorbed to aluminum hydroxide gel (Alhydrogel®). *Na*-ASP-2 is an excretory/secretory product produced by infective *N. americanus* larvae upon penetration of human skin. In animal models, vaccination with this recombinant antigen was shown to result in reduced worm burdens after challenge infection. Accordingly, a phase 1 clinical trial of several different dose concentrations of the vaccine was conducted in healthy, hookworm-naïve adults living in the United States which showed the formulation to be safe, well-tolerated and immunogenic.[23]

However, upon testing the vaccine in adults who had previously been infected with hookworm in an endemic area of Brazil (protocol #SVI-06-02), several volunteers in the first (i.e., lowest dose) cohort to be vaccinated developed generalized urticaria within 1-2 hours of immunization. Due to these immediate-type hypersensitivity reactions, vaccinations in this study were halted and

have not been resumed. Subsequent investigations revealed that the volunteers who developed urticaria upon their first dose of *Na*-ASP-2 had elevated levels of baseline (i.e., pre-vaccination) IgE to the vaccine antigen (**Figure 1**). In order to investigate the prevalence and age distribution of IgE to *Na*-ASP-2 in a population living in a typical hookworm-endemic area, a seroepidemiological survey was conducted in an endemic region of Brazil; this study revealed that even in young children, a significant proportion of individuals have detectable levels of IgE to this protein, likely due to previous infection with *N. americanus* (**Figure 2**). In addition, similar findings were demonstrated for other larval proteins that were being considered as vaccine candidates.

Figure 1: Vaccination of Brazilian Adults with Previous Hookworm Infection with 10 µg *Na*-ASP-2/Alhydrogel® induced immediate-type urticarial reactions. Anti-*Na*-ASP-2 IgE antibody levels were measured in sera collected immediately prior to the first vaccination by ImmunoCAP in individuals vaccinated with the Butang hepatitis B vaccine (n=2, black squares) or 10 µg *Na*-ASP-2/Alhydrogel. For those receiving *Na*-ASP-2, IgE antibody levels are shown for individuals who developed urticaria (n=3, red circles) and those who did not (n=4, grey circles).

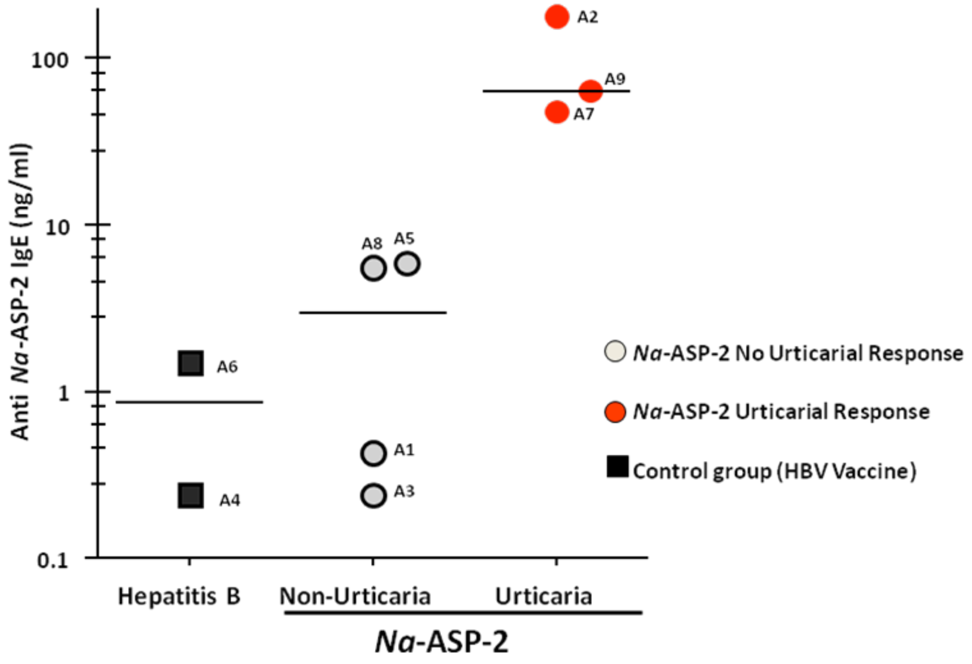

Figure 2: Anti-*Na*-ASP-2 IgE antibody levels in residents of a hookworm-endemic region of northeastern Minas Gerais state, Brazil. Antibody levels were measured by ELISA; each dot represents an individual. Levels are shown for individuals of various age groups, in addition to the 9 participants of the Phase 1 trial of Na-ASP-2 in Brazil (protocol #SVI-06-02) and the participants of the first Phase 1 trial of *Na*-ASP-2 that was conducted in hookworm-naïve adults in the United States (protocol #SVI-04-01).[23]

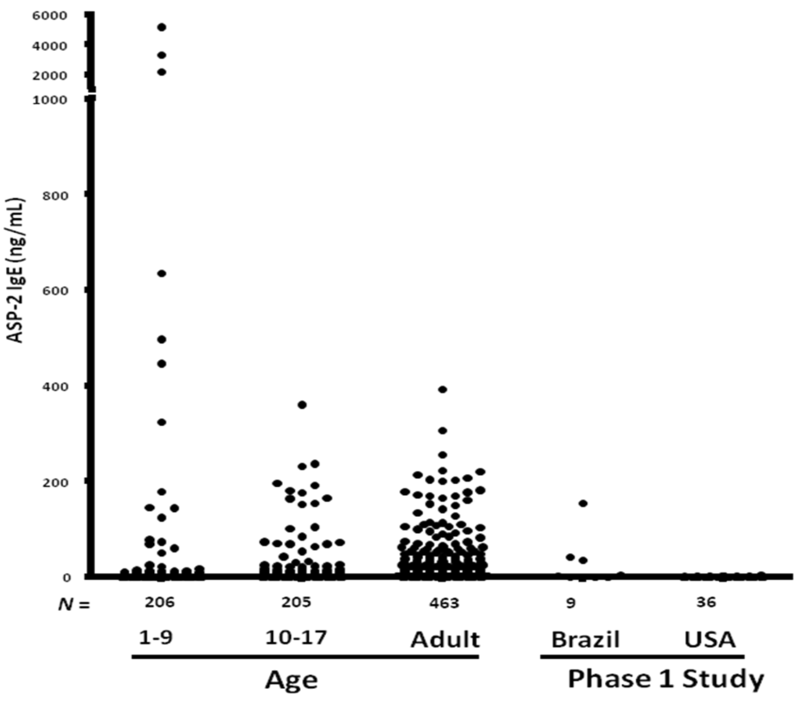

Due to these cumulative data, clinical development of the *Na*-ASP-2 and other larval-stage antigens as candidate vaccines has been halted. Instead, the current strategy is to develop antigens expressed during the adult stage of the hookworm life cycle that play a role in digesting the host hemoglobin that is used by the worm as an energy source. These antigens are relatively hidden from the human immune system during natural infection, and hence have a lower likelihood of inducing antigen-specific IgE in exposed/infected individuals. This should make the potential for inducing allergic reactions upon vaccination much less likely.

1.4 Rationale for Developing the *Na*-GST-1 Hookworm Vaccine

The nutritional and metabolic requirements of the adult hookworm living in the human intestine are dependent upon degradation of host hemoglobin that has been ingested by the worm. *N. americanus* hookworms depend on host hemoglobin for survival;[24] following hemolysis, adult hookworms use an ordered cascade of hemoglobinases to cleave hemoglobin into smaller molecules.[24-29] Following hemoglobin digestion, the freed heme generates toxic oxygen radicals that can be bound and detoxified by molecules such as glutathione S-transferase-1 (GST-1).[30-32]

GST-1 of *N. americanus* (*Na*-GST-1) is a critical enzyme that plays a role in parasite blood feeding; when used as a vaccine, we hypothesize that the antigen will induce anti-enzyme neutralizing antibodies that will interfere with parasite blood-feeding and cause parasite death or reduce worm fecundity.

Na-GST-1 is a 24 kDa protein with peroxidase enzymatic activity that catalyzes the conjugation of reduced glutathione to a variety of electrophiles.[30-32] This hookworm protein belongs to a recently named Nu class of nematode GSTs, which also includes GSTs from the blood-feeding parasite of ruminants, *Haemonchus contortus*, and the rodent nematode *Heligomosoides polygyrus*. This new class is characterized by diminished peroxidase activity relative to other classes of GSTs, but elevated binding capacity for heme and related products.[30, 32-35] X-ray crystallography of *Na*-GST-1 demonstrates that the protein can form homodimers in solution, which create atypically large binding cavities accessible to a diversity of ligands, including heme (**Figure 3**).[32] *Na*-GST-1 binds heme at high affinity *in vitro*.[30, 35] Because both heme and hematin contain oxidative iron, these molecules are potent generators of toxic reactive oxygen species that could potentially damage parasite macromolecules. *In vivo*, hookworm GSTs may therefore bind and detoxify the heme and hematin byproducts generated during the blood degradation process.

Figure 3: Comparison of GST dimers from parasitic nematodes, *N. americanus* (*Na*) and *Heligomosoides polygyrus* (*Hpol*) of the Nu class and comparison with a GST of the Sigma class. A) Superposition of GST dimers reveals that they are very similar, however, Nu class (*Na*-GST-1, magenta; *Na*-GST-2, gold; *Hpol*GST, green) have a more accessible binding cavity than Sigma class (*Hs*GST, cyan). The path to the binding cavity is indicated by the red arrow. The surface plots of Nu class GSTs b) *Hpol*GST, c) *Na*-GST-1, d) *Na*-GST-2 reveal larger access way to binding cavity than e) Sigma class GST (*Hs*GST).[32]

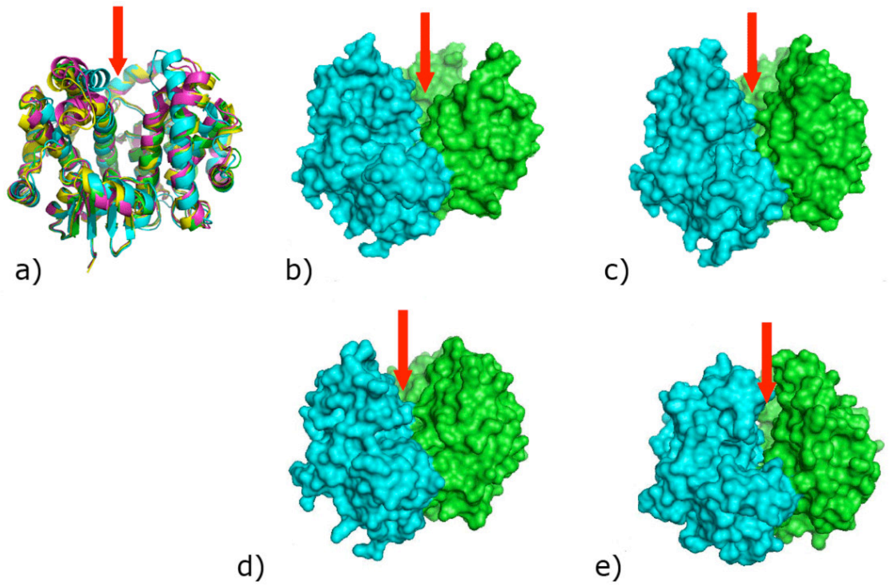

Based on their putative role in hookworm blood feeding, both *Na*-GST-1 and its orthologue from the canine hookworm *Ancylostoma caninum* (*Ac*-GST-1) were tested as experimental vaccines. In dogs, vaccination with yeast-expressed recombinant *Ac*-GST-1 resulted in high levels of antigen-specific antibody (**Figure 4**); following challenge with *A. caninum* infective larvae, significantly lower host worm burdens and fecal egg counts were observed compared to control animals vaccinated only with adjuvant.[30] In hamsters, vaccination with recombinant *Ac*-GST-1 also resulted in substantially lower worm burdens (51-54%) following heterologous challenge with *N. americanus* infective larvae compared to controls, as did vaccination with recombinant *Na*-GST-1 followed by homologous larval challenge.[30, 31, 36] Because of these encouraging preclinical results, *Na*-GST-1 was manufactured according to current good manufacturing practices (cGMP) and formulated on Alhydrogel® in preparation for clinical trials.

Figure 4: Geometric mean titers of the IgG1 and IgG2 antibody responses of vaccinated dogs against r*Ac*-GST-1 formulated with GlaxoSmithKline's AS03 adjuvant. Vaccination time points (1, 2, 3, and 4) and challenge day (C) are marked with arrows.[30]

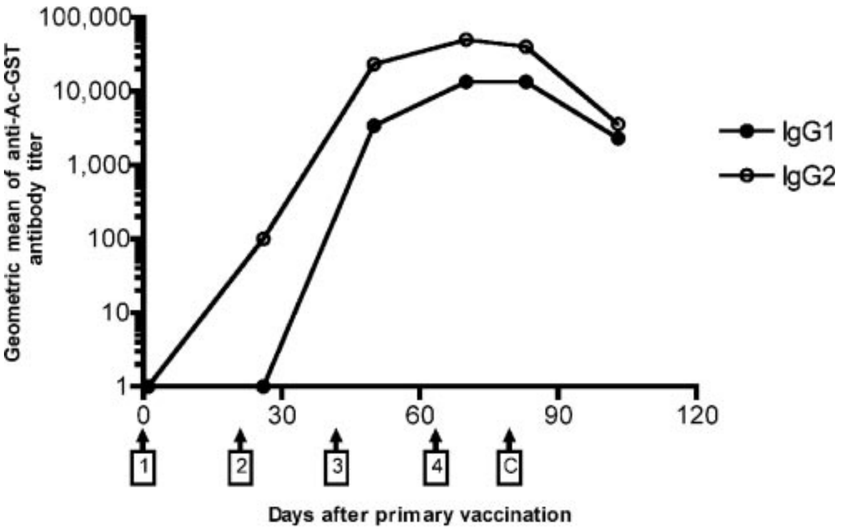

Most importantly, extensive studies have been conducted to test for sensitization to the *Na*-GST-1 protein in individuals living in a hookworm endemic area who have been repeatedly exposed and infected with *N. americanus*. As shown in **Figure 5**, over 1000 individuals of all ages from a hookworm endemic area of Brazil have been tested for serum IgE antibodies to *Na*-GST-1 using an indirect ELISA. In addition, a subset of these serum samples stratified by age and infection status (n = 179) underwent confirmatory testing at the Johns Hopkins Dermatology, Allergy and Clinical Immunology Reference Laboratory (Baltimore, Maryland) using a custom ImmunoCAP assay. The ImmunoCAP method is considered the standard for measuring specific IgE to antigens in serum. This confirmatory testing demonstrated that none of the samples had *Na*-GST-1 IgE values above the clinical cut-off of 0.35 kU_A/L (**Figure 6**).

An advisory meeting was held by the HHVI on March 19, 2010, at George Washington University with several external experts to review the cumulative immunoepidemiology data on *Na*-GST-1. Based on the results of the testing of a population living in a highly endemic area for IgE to *Na*-GST-1 (by both an indirect ELISA and the ImmunoCAP method), the opinion of the advisory group was that the likelihood of inducing immediate-type hypersensitivity reactions by vaccinating individuals living in hookworm-endemic areas with *Na*-GST-1 is low and likely not more than that associated with any new vaccine antigen entering clinical trials. The situation with *Na*-GST-1 is therefore very different from that of *Na*-ASP-2 in that repeated infection with hookworm does not seem to induce an IgE response to the antigen, most likely due to the fact that it is a protein found in the digestive tract of adult hookworms and is therefore relatively hidden from the human immune system. This lack of antigen-specific IgE in people living in an area of high transmission has served as a major justification for advancing development of *Na*-GST-1 as a candidate vaccine antigen.

Figure 5: Anti-*Na*-GST-1 IgE levels in (A) adults and children and (B) young children aged 1-10 years (n=128) living in a hookworm-endemic area of Brazil.

IgE levels (optical density at 492 nm) were measured by ELISA.

A.

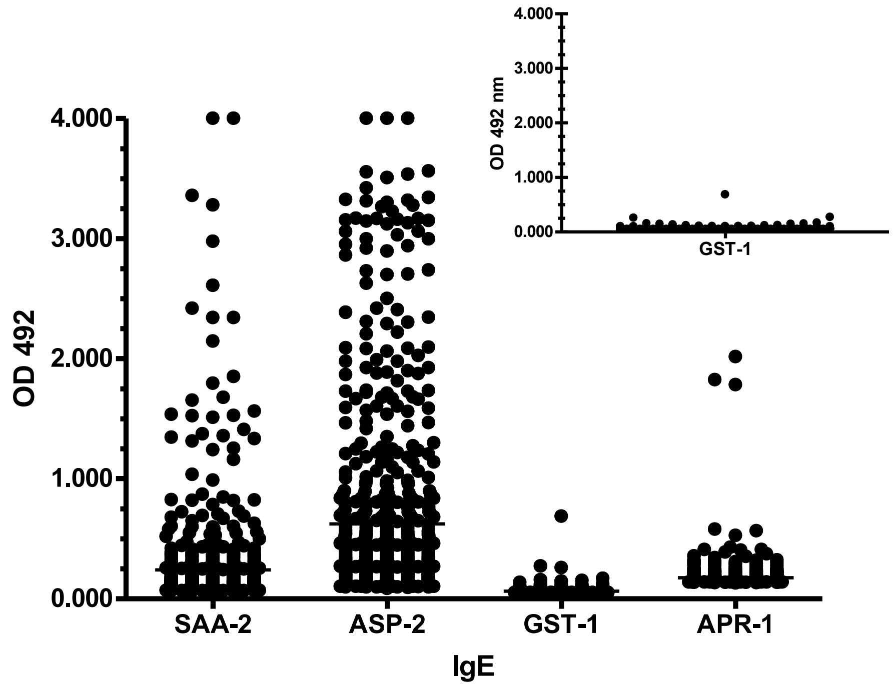

B.

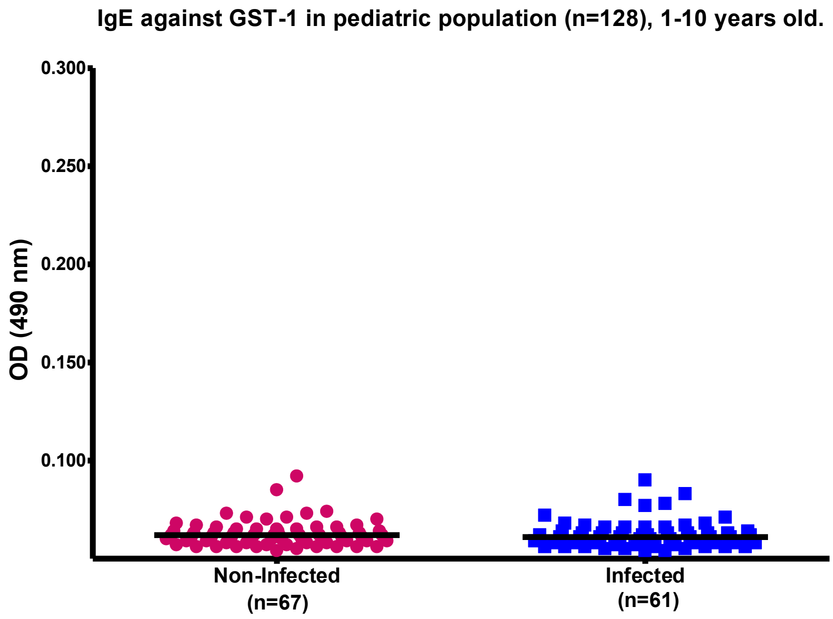

Figure 6: Anti-*Na*-GST-1 IgE levels in a subset of adults and children (n=179) living in a hookworm-endemic area of Brazil. IgE levels (kU$_A$/L) were measured by custom ImmunoCAP.

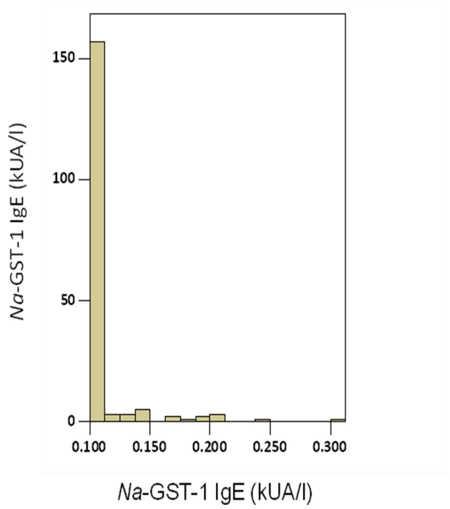

Na-GST-1 will be tested in humans for the first time in the study described in this protocol. *Na*-GST-1 has been successfully manufactured and tested in the laboratory and in animals with both Alhydrogel® and Alhydrogel® plus GLA-AF. *Na*-GST-1 has been shown to be pure, potent, and stable in both of these two formulations.

1.5 *Na*-GST-1 Vaccines

The *Na*-GST-1 vaccine formulations to be tested in this study consist of the 24 kDa recombinant protein *Na*-GST-1, adsorbed to an adjuvant, Alhydrogel® (aluminum hydroxide suspension) either with or without the addition of Gluco-Pyranosylphospho-Lipid A Aqueous Formulation (GLA-AF). For the *Na*-GST-1/Alhydrogel®/GLA-AF formulation, the GLA-AF will be added to the Alhydrogel® formulation immediately prior to immunization. The active ingredient in both vaccine formulations is the recombinant *Na*-GST-1 protein that is derived by fermentation of *Pichia pastoris* yeast cells genetically engineered to express *Na*-GST-1.

1.6 Preclinical Toxicity Study of *Na*-GST-1 Hookworm Vaccines

An 9-week toxicity study of GMP-grade *Na*-GST-1 (Aeras Lot# 09-69F-001) in rats was conducted at Frontier Biosciences, Inc./WestChina-Frontier Pharmatech, Co., China, according to current Good Laboratory Practices. The purpose of the study was to assess potential toxicity of vaccination by multiple IM injections of *Na*-GST-1 adjuvanted with Alhydrogel®, Alhydrogel® plus CPG 10104 and Alhydrogel® plus GLA-AF in Sprague-Dawley Rats. The toxicity study was conducted on an accelerated and extended dosing schedule of one injection on Days 0, 21, 42, and 63. In this rat study, 7 groups of animals (15 males and 15 females per group) were vaccinated by IM injection with 13.5 or 27 μg *Na*-GST-1 in diluent (10% glucose, 10 mM imidazole) or with the same dose levels of *Na*-GST-1 formulated with Alhydrogel®, Alhydrogel® plus CPG 10104 (100 or 300 μg) and Alhydrogel® plus GLA (2 or 20 μg). Four control groups, each consisting of 15 rats per sex, were also vaccinated with either the diluent vehicle, Alhydrogel® alone, Alhydrogel® plus CPG 10104, or Alhydrogel® plus GLA-AF.

Intramuscular injection with *Na*-GST-1/Alhydrogel® or *Na*-GST-1/Alhydrogel®/GLA-AF did not have any adverse effects on survival, body weights, or organ weights or produce any clinical signs of toxicity or abnormal gross necropsy findings in the toxicology study in Sprague-Dawley Rats. Thus, this study did not indicate any safety issues with *Na*-GST-1 adjuvanted with Alhydrogel® with or without the addition of GLA-AF.

There were no test article-related mortalities, clinical signs or effects on body weights or food consumption. Hematology, clinical chemistry, gross pathology and organ weights were unaffected at any dose. Histopathological evaluation of tissues revealed no unexpected effects associated with treatment with *Na*-GST-1 adsorbed to Alhydrogel® or *Na*-GST-1/Alhydrogel® co-administered with GLA-AF. Mild local granulomatous inflammation was observed in the muscle and occasionally in the skin of the injection site in groups treated with formulations containing Alhydrogel® with or without *Na*-GST-1 or the combination of *Na*-GST-1/Alhydrogel® plus GLA-AF. This finding was likely related to the Alhydrogel®, which is well-known to cause granulomatous inflammation.

1.7 Immunogenicity Studies with *Na*-GST-1

Several preclinical animal studies have been conducted in both mice and rats to assess the immunogenicity of *Na*-GST-1 in combination with different adjuvants. First, a study conducted in Sprague-Dawley Rats (HR-25-00) demonstrated that the addition of an adjuvant to recombinant *Na*-GST-1 was necessary, since administration of the recombinant protein without an adjuvant resulted in minimal specific antibody responses.

Figure 7: Anti-*Na*-GST-1 IgG levels (arbitrary units) in Sprague-Dawley Rats vaccinated with recombinant *Na*-GST-1. Rats were vaccinated twice on days 0 and 28 with 60 µg *Na*-GST-1 with or without Alhydrogel (480 µg), Alhydrogel (480 µg) + CPG 10104 (50 µg), or Alhydrogel (480 µg) + CPG 10105 (50 µg). Antibody measurements were performed on blood collected 2 weeks after the final vaccination.

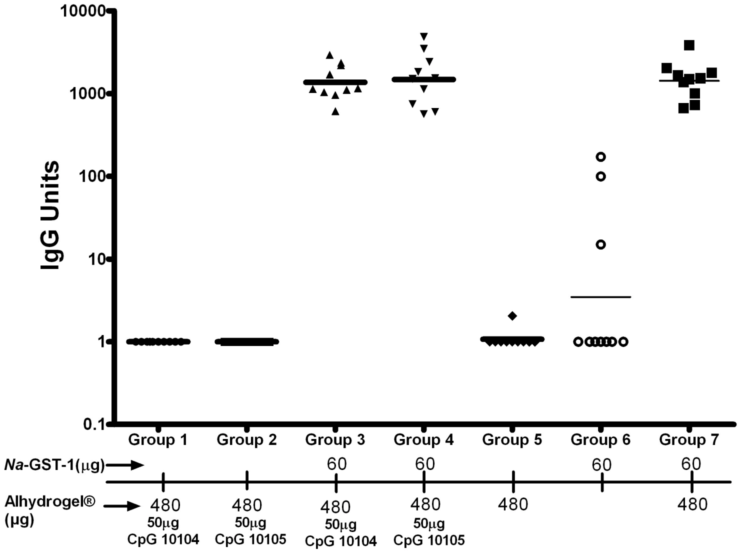

A second study was conducted in BALB/c mice to assess the effect of co-administering CPG 10104 with recombinant *Na*-GST-1/Alhydrogel® (**Figure 8**). In this study, mice were vaccinated with *Na*-GST-1/Alhydrogel® at antigen doses ranging from of 0.01 to 31.25 µg *Na*-GST-1 with or without CPG 10104 (5 µg) or CPG 10105 (5 µg). CPG 10105 is a CpG oligodeoxynucleotide sequence that is similar to CPG 10104 but that is not being proposed to be tested in the study described in this protocol. Mice were vaccinated twice intramuscularly at a 3-week interval, with blood collected for anti-*Na*-GST-1 IgG ELISA two weeks after the second immunization. This study demonstrated a large, highly significant increase in IgG specific for *Na*-GST-1 in the group administered *Na*-GST-1/Alhydrogel®/CPG 10104 compared to that administered only *Na*-GST-1/Alhydrogel® as shown in **Figure 8**.

Figure 8: Geometric mean anti-*Na*-GST-1 IgG antibody units 2 weeks after the 2nd Immunization of BALB/c mice with *Na*-GST-1/Alhydrogel® with or without co-administration of CPG 10104 and CPG 10105.

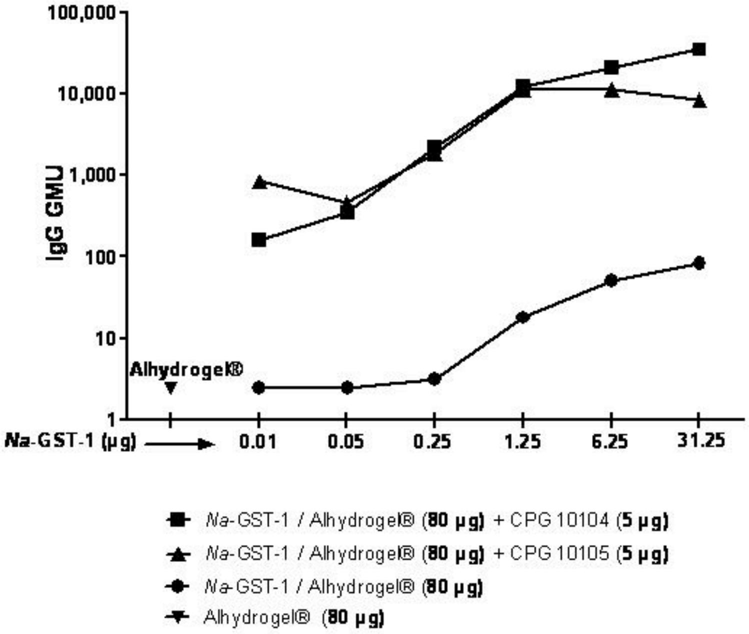

1.8 *In vitro* Tissue Cross-Reactivity Studies

GST-1 is an important detoxification enzyme that exists in different species of organisms. *Na*-GST-1 belongs to a nematode-specific Nu class GST family that is different from human GST classes of protein (e.g., alpha, kappa, mu, omega, pi, theta, zeta and microsomal GSTs), both structurally and functionally. Nu class GSTs have diminished peroxidase activity compared to other classes of GSTs, but elevated binding capacity for heme and related products. The sequence homology between *Na*-GST-1 and GSTs expressed in human tissues ranges from 10-36%. The human protein that shares the highest (36%) amino acid homology with *Na*-GST-1 – GSTS (prostaglandin D synthase) – is expressed abundantly in placenta, lung and liver and moderately in heart and lymph node tissues. Since recombinant *Na*-GST-1 is being developed as a vaccine for humans, there is a theoretical concern that anti-*Na*-GST-1 antibodies induced by immunizing with the recombinant protein might cross-react with human GSTs expressed in human tissues. Western-blot assays using anti-*Na*-GST-1 and commercially-available anti-human GSTK1 (a protein expressed mostly in human liver, lung, thymus and lymph node tissues) antibodies were used to evaluate this potential cross-reactivity.

In these experiments, pooled immune sera from mice vaccinated with recombinant *Na*-GST-1 only recognized recombinant *Na*-GST-1 or native *Na*-GST-1 in the extracts of *N. americanus* adult worms, but not human GST proteins (GSTK1, GSTT1 and GSTA1) or native GSTs in human tissue lysates of liver, brain, spleen, lung, heart, placenta, lymph node and thymus tissue. Likewise, monoclonal anti-human GSTK1 antibodies specifically recognized human GSTK1

protein or native GSTK1 expressed mainly in human liver, lung, heart, lymph node and thymus tissues, but not any recombinant *Na*-GST-1 or *N. americanus* adult extracts. Based upon the current data generated in these *in vitro* studies, it appears unlikely that antibodies induced by vaccination with recombinant *Na*-GST-1 will cross-react with GST proteins expressed in human tissue.

1.9 Clinical Experience with *Na*-GST-1

The recombinant *Na*-GST-1 protein has not yet been tested in humans. The phase 1 trial described in this protocol will be the first time that *Na*-GST-1 will be tested in a clinical trial.

1.10 Clinical Experience with Aluminum-Based Adjuvants

Several licensed vaccines contain aluminum-based adjuvants, including the recombinant Hepatitis B vaccine (e.g., Recombivax® and Butang®), the tetanus toxoid vaccine, and the diphtheria-tetanus toxoids vaccine.[37, 38] For these aluminum hydroxide-adsorbed vaccines, local reactions such as pain, tenderness, and swelling are experienced in between 7.6% and 16.7% of volunteers in studies that included over 1,200 healthy adults. Fever is seen in 3.2% to 9.3%, headache in 4.1%, and other systemic symptoms such as fatigue, malaise, nausea, and diarrhea at lower frequencies. Urticaria has been reported in 0.1% of individuals vaccinated with the hepatitis B vaccine.

1.11 Clinical Experience with GLA-AF

Gluco-Pyranosylphospho-Lipid A Aqueous Formulation (GLA-AF, Infectious Diseases Research Institute [IDRI], Seattle, WA) contains a synthetic monophosphoryl lipid A (MPL) molecule that has TLR4 agonist activity. MPL is itself derived from the lipopolysaccharide (LPS) of *Salmonella minnesota*, a natural TLR4 agonist that is pyrogenic and can induce toxic shock. LPS, and more specifically, its lipid A component, has long been known for its strong adjuvant effects; however, its high toxicity has precluded its use in a vaccine formulation. Ribi et al[22] showed that the monophosphorylated form of lipid A retains its adjuvant function and almost completely loses its endotoxin effects.

Although GLA-AF has not yet been administered to humans, an oil-in-water emulsion of GLA (GLA-SE) has been used in combination with the Fluzone® trivalent killed influenza vaccine in a Phase 1 trial. In this study, doses up to 2.5 µg of GLA-SE were safe and well-tolerated and significantly enhanced influenza-specific antibody responses (unpublished data provided by IDRI).

In addition, there have been many clinical trials involving thousands of participants in which MPL or a derivative have been administered as vaccine adjuvants to adults and children, including vaccines for human papillomavirus, malaria,[39-41] leishmaniasis,[42] and hepatitis B.[43] In general, these trials have demonstrated that administering MPL to humans is safe and well tolerated; when compared to formulations of vaccine that do not contain MPL, those adjuvanted with MPL may result in a minor increase in the incidence and/or severity of local injection site reactions. However, the addition of MPL also often results in a much improved specific antibody response to the vaccine antigen(s).

Of note, MPL is one of the components of the newly licensed Cervarix® vaccine (GlaxoSmithKline, Research Triangle Park, NC) for the prevention of cervical cancer due to human papillomavirus serotypes 16 and 18. The adjuvant for this vaccine consists of MPL adsorbed to aluminum hydroxide salt and is therefore similar to the combination of GLA-AF and Alhydrogel® that we propose testing in combination with *Na*-GST-1 in the study described in this protocol. The Cervarix® vaccine has been shown to have a very favorable safety profile after having been tested in tens of thousands of healthy females.[44, 45]

1.12 Clinical Development Plan

The phase 1 trial described in this protocol will first enroll healthy adults who have not been infected with or exposed to hookworm, in the urban center of Belo Horizonte, Brazil. Hookworm in Brazil is primarily a rural disease and is uncommon in developed urban areas such as Belo Horizonte. If no significant safety concerns are observed in these volunteers, the study will proceed to enroll and vaccinate healthy adults in a hookworm-endemic region of the state of Minas Gerais, Brazil. The vaccine formulations will be tested in both populations (i.e., non-endemic and endemic areas), given the possibility that the safety profile of the vaccine formulations may be different in a population that has been chronically exposed to the antigen contained in these vaccines.

Provided no safety concerns are observed during the adult phase 1 study described in this protocol, age de-escalation, phase 2, and eventually phase 3 clinical trials will be undertaken in endemic areas. A phase 1 trial of *Na*-GST-1 in Brazilian school-aged children is being planned for 2015, assuming no safety issues are identified in the adult study described in this protocol. The decision to proceed to phase 1 testing in hookworm-exposed children will be taken after an interim report of this study has been reviewed by the investigators, the Safety Monitoring Committee (SMC), and the Sponsor (see **Section 9.2.1**). This interim report will include the safety and immunogenicity data of all participants following three immunizations. It is anticipated that the interim report will be available for review in mid-2013.

1.13 Comparator Vaccine

1.13.1 Rationale for use of a comparator vaccine

Having a comparator vaccine is particularly useful in phase 1 trials conducted in endemic areas, since background immunity and natural exposure to hookworm infection may otherwise make it difficult to interpret immunogenicity data. The use of a control group will permit comparison of immune responses between those vaccinated with *Na*-GST-1 and those not vaccinated with this antigen, and will result in a clearer interpretation of immunogenicity results. Although a placebo control group would accomplish the same goal, using a comparator vaccine that is beneficial to the participants increases the benefit-risk ratio, which is always relatively low in phase 1 trials.

Furthermore, the use of a comparator vaccine in a blinded study reduces potential investigator bias in reporting adverse events or attributing their causality. It will also safeguard against the probability that an excess of adverse events will be reported in this trial in comparison to an equivalent trial conducted in the United States. From prior experience conducting other trials at this study site, there will be many reported adverse events that are unrelated to the study

interventions: this rural population has reduced access to medical care and performs excessive heavy labour compared to an urban population or one living in the developed world. Thus, this population has significantly more medical afflictions than would be expected in a study population in the United States. Having a comparator arm in the study enables a fairer assessment of whether or not the rate of adverse events is "normal" or not, since to a reviewer unfamiliar with the study site, viewing the number of adverse events might otherwise lead to an erroneous – and negative – assessment of the vaccine's safety.

1.13.2 Rationale for use of the Hepatitis B vaccine as a comparator

We have chosen the Butang® hepatitis B vaccine (Instituto Butantan, São Paulo, Brazil) as the comparator vaccine for five principal reasons: (1) it is likely to confer some benefit to the participants receiving it, (2) it has an established safety record, (3) it is a yeast-derived recombinant protein adsorbed to an aluminum-containing adjuvant, (4) it has the same physical appearance as *Na*-GST-1 adjuvanted with Alhydrogel, and (5) its dosing schedule permits easy incorporation into the study design. Furthermore, Butang® is the most widely-used hepatitis B vaccine in Brazil, with over 15 million doses distributed annually.

Available data suggest that most adults living in rural Brazil have not received a primary series of immunizations for hepatitis B. In Brazil as a whole, routine hepatitis B vaccination for infants began in 1996 when it was incorporated into the country's Expanded Program for Immunization. It is likely that many adults living in Americaninhas have not yet received this vaccine. Although data from the study area on the seroprevalence of markers of hepatitis B infection are scarce, one study of army conscripts from the northeastern part of the state of Minas Gerais (in which the study site is located) revealed a seroprevalence of 3.9% for hepatitis B surface antigen (HBsAg).[46] However, even though a proportion of potential study participants may have been exposed to hepatitis B and have developed antibodies to HBsAg, vaccination of immune individuals poses no safety hazards.

2 OBJECTIVES

2.1 Primary Objective

1. To determine the frequency of vaccine-related AEs, graded by severity, for each dose and formulation of *Na*-GST-1.

2.2 Secondary Objectives

1. To determine the dose and formulation of *Na*-GST-1 that generates the highest IgG antibody response at Day 126, as determined by an indirect enzyme-linked immunosorbent assay (ELISA).
2. To assess and compare the duration of antibody response to *Na*-GST-1.
3. To perform exploratory studies of the cellular immune responses to the *Na*-GST-1 antigen both before and after immunization.

3 STUDY SITES

The study will be conducted in two parts as outlined in **Section 4** below. The first part of the study will be conducted in healthy adult volunteers without history of hookworm infection at the

Centro de Pesquisas Rene Rachou (CPqRR) in Belo Horizonte, Minas Gerais, Brazil. Belo Horizonte is a large city of approximately 4-5 million inhabitants. Although there are parts of the city where residents live in crowded conditions without access to adequate clean water and sanitation and where transmission of hookworm and other soil-transmitted helminths may occur, the majority of people living in this urban center have not been exposed to or infected with these parasites. The CPqRR is a research institution that is part of the Fundação Oswaldo Cruz (FIOCRUZ) network that is the division of the Brazilian federal Ministry of Health dedicated to conducting research on issues of importance to the health of Brazilians.

The second part of the study will be conducted in Americaninhas, a town located in the Municipality of Novo Oriente de Minas, 500 km northwest of Belo Horizonte, the capital of the Brazilian state of Minas Gerais. The Brazilian Fundação National de Saude (National Health Foundation) estimates that there are 1000 people living in the urban municipal center of Americaninhas, with another 4000 living in the surrounding rural areas. The area is hilly and characterized by a tropical altitude climate, with an average temperature of 24°C, and experiences a long rainy season between November and March; annual rainfall is 1300-2000mm. The majority of inhabitants are involved in rural subsistence farming, growing mainly coffee, manioc and beans. Cattle ranching is another important source of income. Houses are predominantly made from concrete or from a combination of wood and mud and have either tiles or iron sheets for roofing. Only approximately 50% of these homes have a latrine and people commonly collect their water from local springs. There is only one health post in the area staffed by two auxiliary health workers who are paid by the municipality.

Americaninhas has been chosen as a site for testing hookworm vaccines based on prevalence surveys conducted over the past few years, and the good working relationship that has been established between the research staff, the local health authorities, and the community.

Starting in 2004, epidemiological studies have been conducted by the study team in the town of Americaninhas and surrounding areas in order to identify the populations at greatest risk for heavy hookworm infection and to conduct post-treatment hookworm re-infection studies in order to inform the sample size calculations for phase 1 and phase 2 studies in the region. These studies have been challenging because of a number of physical challenges including the isolation of the region, the hilly terrain, and the large distances between dwellings. Geographic Information System/Remote Sensing identified high levels of vegetation as a critical feature of the infection sites. Overall, the force of infection in Minas Gerais is high. It was estimated that post-treatment hookworm infection occurs in approximately 300 per 1,000 individuals per year, which represents a level of transmission that is comparable to other areas considered to have intense or high transmission, such as in East Africa. The overall prevalence of *N. americanus* hookworm infection is approximately 68 percent, with approximately one-half of the population also infected with *Ascaris lumbricoides* and with *Schistosoma mansoni*. As in other regions of the world, the prevalence of hookworm infection rises in the first 5 years of life when it reaches a plateau. In addition, the intensity of hookworm infection (as measured by fecal egg counts using the Kato-Katz technique) also rises until the age of 5 years and then remains high. A second increase in hookworm intensity occurs after the age of 50.

4 STUDY DESIGN

4.1 Overall Design

<u>The study will be conducted in two parts</u> (**Table 1**). **Part I** of the study will be an open-label dose-escalation phase 1 trial of *Na*-GST-1/Alhydrogel® administered with or without GLA-AF conducted in Belo Horizonte in healthy adult volunteers <u>without</u> history of hookworm infection. **Part II** will be a randomized, controlled, double-blind phase 1 dose-escalating clinical trial in healthy adult volunteers living in the hookworm-endemic area of Americaninhas. This part of the study is designed to evaluate the safety, reactogenicity, and immunogenicity of *Na*-GST-1/Alhydrogel® with or without GLA-AF, as compared to Butang® hepatitis B vaccine.

Safety parameters will be monitored throughout both parts of the study. The primary immunological endpoint for both parts will be the measurement of the antigen-specific antibody response.

Prior to beginning study-related activities in Americaninhas in Part II of the study, the consent and cooperation of representative community members will be sought, after which volunteers will be invited to participate in the study. In both Belo Horizonte and Americaninhas, after providing written informed consent and passing the informed consent comprehension questionnaire, volunteers will undergo eligibility screening, including a complete medical history, physical examination, hematology and coagulation testing, serum glucose testing, liver and renal function testing, HIV antibody testing, Hepatitis B and C serology, and urinalysis; urine pregnancy testing will be performed on all female volunteers. In addition, all volunteers will be tested for serum IgE antibodies to *Na*-GST-1, and a fecal examination will be performed for ova and parasites. All clinically significant abnormalities will be reviewed with volunteers and referral for follow-up care will be provided. After screening, those volunteers determined to be eligible, based on the inclusion and exclusion criteria described in **Section 5** in this protocol, will be invited to participate in the study.

Dose cohorts will be enrolled and vaccinated in a consecutive fashion as described in **Section 4.3**. In **Part I** of the study, 36 volunteers will be enrolled into one of six cohorts (see **Table 3**): in the first cohort, 6 individuals will receive the 10 µg dose of *Na*-GST-1/Alhydrogel®; in the second cohort, 6 individuals will receive the 10 µg dose of *Na*-GST-1/GLA-AF; in the third and fourth cohorts, 6 volunteers each will receive 30 µg *Na*-GST-1/Alhydrogel® and *Na*-GST-1/Alhydrogel®/GLA-AF, respectively; while in the fifth and sixth cohorts, 6 volunteers each will receive 100 µg *Na*-GST-1/Alhydrogel® and *Na*-GST-1/Alhydrogel®/GLA-AF, respectively. Enrollment of these cohorts will be staggered by (at least) one-week intervals: Cohorts 2 and 3 will be enrolled and begin vaccinations at least 1 week after Cohort 1, Cohorts 4 and 5 will be vaccinated at least 1 week after Cohorts 2 and 3, and Cohort 6 will be vaccinated at least 1 week after Cohorts 4 and 5.

In **Part II** of the study, 66 volunteers will be progressively enrolled into one of six dose cohorts. In the seventh, ninth and eleventh cohorts, volunteers will be randomized to receive either *Na*-GST-1/Alhydrogel® (n=8) or Butang® (n=2), while in the eighth, tenth and twelfth cohorts, volunteers will be randomized to receive either *Na*-GST-1/Alhydrogel® (n=2) or *Na*-GST-

1/Alhydrogel®/GLA-AF (n=10). Volunteers randomized to receive *Na*-GST-1 will receive either the 10 µg, 30 µg or 100 µg dose, as outlined in **Table 3**.

As with other aluminum hydroxide-adsorbed vaccines, hypersensitivity reactions would be expected to occur within the first 24 hours after receipt of either of the two vaccines, and other severe local or systemic reactions within 72 hours of vaccination. Participants will therefore be observed for immediate reactions following each vaccination for at least 2 hours, and will have a clinical assessment either in their home or at the study clinic on Days 1, 3, 7, and 14 following each vaccination. See **Tables 2** and **3** for tabular descriptions of the vaccination schedule for all dose cohorts, as well as **Section 7.6** and **Appendix B** for a detailed description of the scheduled clinical and laboratory evaluations.

Prior to enrolling and vaccinating volunteers in Part II of the study, safety data up to and including Day 70 post-vaccination from the sixth cohort (i.e., the cohort administered 100 µg *Na*-GST-1/Alhydrogel®/GLA-AF) will be reviewed by the Safety Monitoring Committee (SMC). The trial will not proceed to Part II of the study if any of the stopping criteria listed in **Section 8.5** are met, or if in the clinical judgment of the SMC, continued enrollment and vaccinations would pose an unacceptable safety risk to the participants.

In Part II of the study, prior to dose escalation (e.g., after enrolling Cohort 7 but before enrolling Cohort 9), safety data up to and including Day 7 post-vaccination will be compiled from the lower dose cohort for review by the Medical Monitor and/or SMC. The trial will not proceed to the next dose cohort if any of the stopping criteria listed in **Section 8.5** are met or, in the clinical judgment of the SMC and/or Medical Monitor, the next higher dose would pose an unacceptable safety risk to the participants.

Table 1: Study Design

	Cohort	Dose *Na*-GST-1	Cohort Characteristics	Total
Part 1	1	10 µg	6 *Na*-GST-1/Alhydrogel®	36
	2	10 µg	6 *Na*-GST-1/Alhydrogel®/GLA-AF	
	3	30 µg	6 *Na*-GST-1/Alhydrogel®	
	4	30 µg	6 *Na*-GST-1/Alhydrogel®/GLA-AF	
	5	100 µg	6 *Na*-GST-1/Alhydrogel®	
	6	100 µg	6 *Na*-GST-1/Alhydrogel®/GLA-AF	
Part 2	7	10 µg	8 *Na*-GST-1/Alhydrogel® + 2 hepatitis B	10
	8	10 µg	2 *Na*-GST-1/Alhydrogel® + 10 *Na*-GST-1/ Alhydrogel®/GLA-AF	12
	9	30 µg	8 *Na*-GST-1/Alhydrogel® + 2 hepatitis B	10
	10	30 µg	2 *Na*-GST-1/Alhydrogel® + 10 *Na*-GST-1/ Alhydrogel®/GLA-AF	12
	11	100 µg	8 *Na*-GST-1/Alhydrogel® + 2 hepatitis B	10
	12	100 µg	2 *Na*-GST-1/Alhydrogel® + 10 *Na*-GST-1/ Alhydrogel®/GLA-AF	12
**Total**			48 *Na*-GST-1/Alhydrogel® + 48 *Na*-GST-1/GLA-AF + 6 hepatitis B	**102**

Table 2: Part I Vaccination Schedule

Time[1] (Calendar Week)	**Cohort 1** (n = 6)<br><br>10 µg *Na*-GST-1 80 µg Alhydrogel®	**Cohort 2** (n = 6)<br><br>10 µg *Na*-GST-1 80 µg Alhydrogel®+ 2.5 µg GLA-AF	**Cohort 3** (n = 6)<br><br>30 µg *Na*-GST-1 240 µg Alhydrogel®	**Cohort 4** (n = 6)<br><br>30 µg *Na*-GST-1 240 µg Alhydrogel®+ 2.5 µg GLA-AF	**Cohort 5** (n = 6)<br><br>100 µg *Na*-GST-1 800 µg Alhydrogel®	**Cohort 6** (n = 6)<br><br>100 µg *Na*-GST-1 800 µg Alhydrogel® + 2.5 µg GLA-AF
0	Vaccination 1 (Day 0)					
1		Vaccination 1 (Day 0)	Vaccination 1 (Day 0)			
2				Vaccination 1 (Day 0)	Vaccination 1 (Day 0)	
3						Vaccination 1 (Day 0)
8	Vaccination 2 (Day 56)					
9		Vaccination 2 (Day 56)	Vaccination 2 (Day 56)			
10				Vaccination 2 (Day 56)	Vaccination 2 (Day 56)	
11						Vaccination 2 (Day 56)
16	Vaccination 3 (Day 112)					
17		Vaccination 3 (Day 112)	Vaccination 3 (Day 112)			
18				Vaccination 3 (Day 112)	Vaccination 3 (Day 112)	
19						Vaccination 3 (Day 112)

[1]Approximate (see **Section 7.6** for more detail). Weeks between enrollment of successive cohorts are the minimum required.

Table 3: Part II Vaccination Schedule

Time[1] (Calendar Week)	**Cohort 7** (n = 10)<br><br>10 µg *Na*-GST-1 80 µg Alhydrogel® (n=8) **OR** Butang® (n=2)	**Cohort 8** (n = 12)<br><br>10 µg *Na*-GST-1 80 µg Alhydrogel® (n=2) **OR** 10 µg *Na*-GST-1 80 µg Alhydrogel® + 2.5 µg GLA-AF (n=10)	**Cohort 9** (n = 10)<br><br>30 µg *Na*-GST-1 240 µg Alhydrogel® (n=8) **OR** Butang® (n=2)	**Cohort 10** (n = 12)<br><br>30 µg *Na*-GST-1 240 µg Alhydrogel® (n=2) **OR** 30 µg *Na*-GST-1 240 µg Alhydrogel® + 2.5 µg GLA-AF (n=10)	**Cohort 11** (n = 10)<br><br>100 µg *Na*-GST-1 800 µg Alhydrogel® (n=8) **OR** Butang® (n=2)	**Cohort 12** (n = 12)<br><br>100 µg *Na*-GST-1 800 µg Alhydrogel® (n=2) **OR** 100 µg *Na*-GST-1 800 µg Alhydrogel® + 2.5 µg GLA-AF (n=10)
0	Vaccination 1 (Day 0)					
2		Vaccination 1 (Day 0)	Vaccination 1 (Day 0)			
4				Vaccination 1 (Day 0)	Vaccination 1 (Day 0)	
5						Vaccination 1 (Day 0)
8	Vaccination 2 (Day 56)					
10		Vaccination 2 (Day 56)	Vaccination 2 (Day 56)			
12				Vaccination 2 (Day 56)	Vaccination 2 (Day 56)	
13						Vaccination 2 (Day 56)
16	Vaccination 3 (Day 112)					
18		Vaccination 3 (Day 112)	Vaccination 3 (Day 112)			
20				Vaccination 3 (Day 112)	Vaccination 3 (Day 112)	
21						Vaccination 3 (Day 112)

[1]Approximate (see **Section 7.6** for more detail). Weeks between enrollment of successive cohorts are the minimum required.

4.2 Sample Size and Estimated Duration of Study

A total of 102 participants will be enrolled (see **Section 10.2** for a justification of the sample size). Forty-eight will receive *Na*-GST-1/Alhydrogel® (one of three dose concentrations), 48 will *Na*-GST-1/Alhydrogel®/GLA-AF (one of three dose concentrations), and 6 will receive Butang® hepatitis B vaccine. The trial is expected to last for a total of 24 months. Each participant will be followed for 16 months from the time of the first injection.

In Part II of the study, in order to ensure that the vaccine formulations will be evaluated in individuals recently infected with hookworm, at least 4 individuals will be enrolled into each of Cohorts 7-12 who were infected with hookworm based on the fecal exam conducted during the

screening for the study, or who had a documented infection within 3 months of the date planned for the first vaccination for the participant.

4.3 Group Allocation

Dose cohorts will be enrolled <u>consecutively</u>. In **Part I** of the study, within Cohort 1 all participants will receive the 10 µg dose of *Na*-GST-1/Alhydrogel®; within Cohort 2, all will receive 10 µg of *Na*-GST-1/Alhydrogel®/GLA-AF, and so on. In **Part II** of the study, within Cohorts 7, 9 and 11 of 10 participants, 8 will be randomly assigned to receive *Na*-GST-1/Alhydrogel® and 2 to receive Butang® (see **Section 7.3**). Within Cohort 7, Cohort 9, and Cohort 11, those randomized to receive *Na*-GST-1/Alhydrogel® will receive a dose containing 10 µg, 30 µg and 100 µg of *Na*-GST-1, respectively. Also in Part II of the study, within Cohorts 8, 10, and 12 of 12 participants, 2 will be randomly assigned to receive *Na*-GST-1/Alhydrogel® and 10 to receive *Na*-GST-1/Alhydrogel®/GLA-AF. Within Cohorts 8, Cohort 10, and Cohort 12, participants will receive a dose containing 10 µg, 30 µg and 100 µg of *Na*-GST-1, respectively.

In both **Part I** and **Part II** of the study, 1 or 2 additional eligible volunteers above the number to be enrolled in a given cohort may be scheduled for a Day 0 visit, if possible. On the day of first vaccination, the first volunteers who arrive at the study clinic will be evaluated for continued eligibility and vaccinated in the order that they arrive; the additional eligible volunteers will be kept as alternates if some of the first arrivals cannot be vaccinated on the day of first vaccination (i.e., due to pregnancy, withdrawal of consent, etc.). If the alternates are not vaccinated, they will be invited to participate as members of the next dose cohort. Those alternates for the final cohorts in both Part I and Part II who are not vaccinated and therefore not enrolled in the study, will be offered vaccination with a non-study, licensed vaccine such as the tetanus toxoid or influenza vaccine.

4.4 Blinding

Part I of the study will be an <u>open label</u> phase 1 trial. In **Part II**, investigators and participants will be <u>blinded</u> as to an individual study participant's allocation to either *Na*-GST-1/Alhydrogel®, *Na*-GST-1/GLA-AF or Butang® vaccine until <u>all</u> participants have completed their Day 140 visit, the safety and primary immunogenicity data have been monitored and entered into the database, and the database has been locked. Only the study vaccine manager (and potentially an assistant) will be aware of the participants' vaccine allocation. The study vaccine manager will refer to the unique randomization code assigned to that participant to determine the assigned vaccine for each participant (see **Section 7.3**). Vaccine will be prepared by the study vaccine manager in a separate room and the vaccine-filled syringes will be handed to the vaccinator(s). Since the 10 and 30 µg doses of the *Na*-GST-1 formulations are of different volumes (0.1 ml and 0.3 ml, respectively) than that of Butang® (1.0 ml), the contents of all syringes will be disguised using opaque tape. As a further precaution, the vaccinator(s) will not be based in Americaninhas and will not be involved in assessments of reactogenicity or adverse events.

Due to the staggered, dose-escalation design of Part II of the trial, it will not be possible to blind to the <u>dose</u> of *Na*-GST-1 that an individual may have received. That is, those participants

assigned to Cohort 7 may receive either the 10 µg dose of *Na*-GST-1/Alhydrogel or the Butang® vaccine, whereas Cohort 8 will receive either 10 µg *Na*-GST-1/Alhydrogel or *Na*-GST-1/GLA-AF, Cohort 9 will receive either 30 µg *Na*-GST-1/Alhydrogel or Butang®, and Cohort 10 will receive either 30 µg *Na*-GST-1/Alhydrogel or *Na*-GST-1/GLA-AF, and so on. Since Cohorts 9 and 10 will receive their vaccinations after Cohorts 7 and 8 (see **Table 3**) and Cohorts 11 and 12 will receive their vaccinations after Cohorts 9 and 10, it will not be possible to blind to the dose of *Na*-GST-1 that may have been given, only to the allocation between *Na*-GST-1/Alhydrogel, *Na*-GST-1/GLA-AF, or Butang®.

The investigators will be unblinded to participants' vaccine allocation status after all have had their study Day 140 visit (see **Section 8.10).** After this point, the study will be single-blinded (i.e., study participants will remain blinded to what vaccine they have received). The principal justification for unblinding the investigators before the final study visit has been completed is that the safety and immunogenicity data acquired during the vaccination phase of the study will be crucial in guiding the clinical development plan for *Na*-GST-1, and we feel that, to make an informed decision as to whether or not to proceed to pediatric trials, we must first assess the unblinded data in a timely fashion.

5 SELECTION OF PARTICIPANTS

5.1 Inclusion Criteria

1. Males or females between 18 and 45 years, inclusive.
2. Good general health as determined by means of the screening procedure.
3. Available for the duration of the trial (16 months).
4. Willingness to participate in the study as evidenced by signing the informed consent document.
5. For Part II only: Negative for hookworm during screening, or if found to be infected with hookworm, has completed a course of three doses of albendazole.

5.2 Exclusion Criteria

1. Pregnancy as determined by a positive urine hCG (if female).
2. Participant unwilling to use reliable contraception methods up until one month following the third immunization (if female and not surgically sterile, abstinent or at least 2 years post-menopausal).
3. Currently lactating and breast-feeding (if female).
4. Inability to correctly answer all questions on the informed consent comprehension questionnaire.
5. Evidence of clinically significant neurologic, cardiac, pulmonary, hepatic, rheumatologic, autoimmune, diabetes, or renal disease by history, physical examination, and/or laboratory studies.
6. Known or suspected immunodeficiency.
7. Laboratory evidence of liver disease (alanine aminotransferase [ALT] greater than 1.25-times the upper reference limit).
8. Laboratory evidence of renal disease (serum creatinine greater than 1.25-times the upper reference limit, or more than trace protein or blood on urine dipstick testing).
9. Laboratory evidence of hematologic disease (absolute leukocyte count $<3200/mm^3$ or $<3500/mm^3$ [in Americaninhas and Belo Horizonte, respectively]; absolute leukocyte count $>10.8 \times 10^3/mm^3$; hemoglobin <11.4 g/dl or <12.0 g/dl [females in Americaninhas and Belo Horizonte, respectively] or <12.1 g/dl or <13.5 [males in Americaninhas and Belo Horizonte, respectively]; absolute lymphocyte count $<900/mm^3$; or, platelet count $<130{,}000/mm^3$ or $<140{,}000/mm^3$ [in Americaninhas and Belo Horizonte, respectively]).
10. Laboratory evidence of a coagulopathy (PTT or PT INR greater than 1.1-times the upper reference limit [in Belo Horizonte] or PT INR greater than 1.3 [Americaninhas]).
11. Serum glucose (random) greater than 1.2-times the upper reference limit.
12. Other condition that in the opinion of the investigator would jeopardize the safety or rights of a volunteer participating in the trial or would render the subject unable to comply with the protocol.
13. Participation in another investigational vaccine or drug trial within 30 days of starting this study.
14. Volunteer has had medical, occupational, or family problems as a result of alcohol or illicit drug use during the past 12 months.
15. History of a severe allergic reaction or anaphylaxis.
16. Severe asthma as defined by the need for regular use of inhalers or emergency clinic visit or hospitalization within the last 6 months.

17. Positive ELISA for HCV.
18. Positive ELISA for HBsAg.
19. Positive ELISA for HIV.
20. Use of corticosteroids (excluding topical or nasal) or immunosuppressive drugs within 30 days of starting this study.
21. Receipt of a live vaccine within past 4 weeks or a killed vaccine within past 2 weeks prior to entry into the study.
22. History of a surgical splenectomy.
23. Receipt of blood products within the past 6 months.
24. History of allergy to yeast.
25. Anti-*Na*-GST-1 IgE antibody level above 0.35 kU_A/L by the ImmunoCAP method.
26. For Part I only: history of previous infection with hookworm; residence for more than 6 months in a hookworm-endemic area; or, positive for hookworm infection on screening microscopic fecal examination.
27. For Part II Cohorts #7, #9, and #11 only: previous receipt of a primary series of any hepatitis B vaccine.

6 VACCINE PREPARATION

6.1 Supplies

6.1.1 Na-*GST-1/Alhydrogel®*

Na-GST-1/Alhydrogel® is supplied as a sterile milky-white suspension (when shaken slightly). Each 2.0 ml vial contains 1.35 ml of a 0.1 mg/ml suspension of *Na*-GST-1 adsorbed to 0.8 mg/ml of Alhydrogel® in a buffer consisting of 10% glucose and 10 mM imidazole, pH 7.4. Glucose acts as an excipient and imidazole as the buffer based on evidence that these components specifically enhance the stability and solubility of *Na*-GST-1. The maximum dose that will be administered is 100 µg of *Na*-GST-1, or 1.0 ml of the final drug product. This volume contains the equivalent of approximately 400 µg aluminum. Lower doses of *Na*-GST-1 will be delivered by injecting smaller volumes of the 0.1 mg/ml suspension: for example, 0.3 ml will be injected to deliver 30 µg *Na*-GST-1 and 0.1 ml to deliver 10 µg *Na*-GST-1. For all doses, the ratio of *Na*-GST-1 to Alhydrogel® will therefore remain constant: for the 10, 30 and 100 µg doses of *Na*-GST-1 the respective amounts of Alhydrogel® will be 80, 240 and 800 µg (corresponding to approximately 40, 120 and 400 µg aluminum, respectively). *Na*-GST-1/Alhydrogel® was manufactured, formulated and vialed at Aeras Global Vaccine Foundation (Rockville, Maryland, USA). The product conforms to established requirements of purity, sterility, safety, and identity.

6.1.2 GLA-AF

GLA-AF will be supplied to the trial site as an aqueous solution in multi-dose vials containing 50 µg/mL or 100 µg/mL of GLA without preservative. Appropriate volumes of GLA-AF will be withdrawn from the multi-dose vials using a syringe and added to a vial containing 1.35 mL of *Na*-GST-1/Alhydrogel® or vice versa (described in the Investigator's Brochure). The

mixture must be administered not more than 24 hours after mixing the GLA-AF with *Na*-GST-1/Alhydrogel®.

6.1.3 Butang® Hepatitis B Vaccine

Butang® (Instituto Butantan, São Paulo, Brazil) is a non-infectious subunit viral vaccine derived from hepatitis B surface antigen (HBsAg) produced in yeast cells. The antigen is harvested and purified from fermentation cultures of a recombinant strain of the yeast *Pichia angusta* containing the gene for the *adw* subtype of HBsAg. The purified protein is then adjuvanted with amorphous aluminum hydroxide. Butang® is a slightly turbid sterile suspension for intramuscular injection. Butang® is supplied in multi-dose vials, with each vial containing enough vaccine to deliver 5 1.0 mL doses. Each 1.0 mL dose contains 20 µg of hepatitis B surface antigen and approximately 500 µg of aluminum, in addition to up to 0.05 mg of the preservative thimerosol. The product conforms to established requirements for sterility, safety, and identity.

6.2 Transport of Vaccines

Vials of *Na*-GST-1/Alhydrogel® for this study will be supplied to the study site by the Sponsor. Vials of GLA-AF will be supplied to the study site by IDRI, Seattle, Washington, US, where GLA-AF was manufactured. Butang® will be obtained in Brazil through the Ministry of Health of the state of Minas Gerais. *Na*-GST-1/Alhydrogel®, GLA-AF and Butang® will be transported to CPqRR in Belo Horizonte where they will be stored until used in the study, and to the study sites at 1°C to 9°C; temperature recording devices will accompany the vaccines at all times during transport to ensure temperature limits have not been violated. Vaccines and GLA-AF will be stored in Belo Horizonte in a refrigerator at 2°C to 8°C and will not be frozen; refrigerator temperature will be monitored continuously. For Part II of the study, prior to vaccination, adequate supplies of vaccine and GLA-AF will be transported to the study site in Americaninhas, in temperature-monitored coolers; at the study site, they will be stored in a temperature-monitored refrigerator. All vaccine vials will be stored in the upright position.

6.3 Preparation of Vaccine Doses

The *Na*-GST-1/Alhydrogel® plus GLA-AF formulations will be prepared by adding an appropriate volume of the GLA-AF solution (50 or 100 µg/ml) to a vial of *Na*-GST-1/Alhydrogel®, or vice versa, within 24 hours of vaccination.

6.4 Vaccine Storage

Na-GST-1/Alhydrogel, GLA-AF and Butang® should be maintained at 2°C to 8°C until just prior to administration. Vaccines and GLA-AF should NOT be frozen at any time.

6.5 Vaccine Accountability

Study vaccine and GLA-AF supplies must be received by a designated person at the study sites, handled and stored safely and properly, and kept in a secure location to which only the Investigator and designated assistants have access. Study-site personnel are responsible for maintaining accurate records of the vaccine supplies (i.e., *Na*-GST-1/Alhydrogel®, Butang® and

GLA-AF) received, the quantities administered to study participants, and the amounts remaining at the conclusion of the study.

6.6 Disposition of Used/Unused Supplies

After administration of vaccine or GLA-AF doses, vials will be stored at the study site, and vials will be accounted for and stored until monitoring by the study Sponsor or their designee. The vials may then be disposed of according to site protocol. At the conclusion of the study, all unused *Na*-GST-1/Alhydrogel® and GLA-AF supplies will be destroyed on site upon direction from the Sponsor, returned to the Sponsor, or maintained at 2 to 8°C until further notice from the Sponsor regarding their disposition. All other vials will be retained until requested by the Sponsor.

7 STUDY PROCEDURES

The following sections provide a detailed listing of the procedures and studies to be performed in this protocol at designated time points. The total volume of blood (approximately 540 mL) to be collected from each volunteer over their 16-month participation in the trial is approximately the volume collected when donating one unit of blood and should not compromise the health of trial participants.

7.1 Community Consent for Part II of the Study

Previous studies conducted by the study team in Americaninhas have permitted extensive contact with the local population that has led to the development of mutual trust and the establishment of a familiarity with the informed consent process.

First, a meeting will be held with municipal leaders (municipal mayor and the secretaries of health and education) during which the objectives and procedures of the study will be explained. Second, a meeting will be held with community leaders. Only after this initial contact with the leaders will the first contact with the community be held, by means of a communal meeting. During this meeting, the study team will be presented to the community and the planned study will be explained. After the initial meeting with the entire community, the study team will arrange visits with individual volunteers to conduct the process of informing them about the study, answering questions, and obtaining the volunteer's signature on the informed consent form. These steps allow the process to be conducted in a manner that guarantees that consent will be truly informed, ensuring that the decision of the volunteer to participate in the study is truly autonomous.

7.2 Individual Recruitment and Informed Consent

Volunteers aged 18-45 years, inclusive, will be invited to undergo screening for the study. During this initial screening visit, a member of the study team will read the consent form together with the volunteer, regardless of whether or not the volunteer is illiterate. The study team member will explain why the volunteer is being invited to participate in the study and will clarify all of the volunteer's questions. Volunteers will be encouraged to ask questions, and then take a true/false questionnaire to evaluate consent comprehension (**Appendix A**); this will be administered orally in the case of potential volunteers who cannot read. The volunteer must

answer all questions correctly prior to being eligible for enrollment. Study staff will use incorrect answers from the questionnaire to identify those areas of the informed consent form that need further review with the volunteer. This will help ensure the volunteer has sufficient understanding before the consent form is signed. The volunteer may either sign the consent form immediately or later after further consideration. Volunteers unable to read will place an imprint of their finger in the place of a signature; in addition, an independent witness, who is not a member of the study team, will sign the consent form to attest that the volunteer fully comprehended the contents.

The following procedures will be performed upon initial screening (note that all procedures might not be performed on the same day):

1. Explain the study and Informed Consent to the volunteer.
2. Ensure the subject has passed the informed consent comprehension questionnaire, has signed the Informed Consent and receives a signed copy of the Informed Consent.
3. Elicit a complete medical history, including medication history, and for female subjects, a menstrual and contraceptive history and/or history of surgical sterility.
4. Administer a complete physical examination.
5. Obtain blood for hematology, biochemistry, and serologic tests for HIV, viral hepatitis (B and C) and anti-*Na*-GST-1 IgE.
6. Obtain fecal sample for examination for ova and parasites.
7. Obtain urine for urine dipstick testing, as well as urine hCG testing in females.
8. Counsel females to avoid becoming pregnant during the study.

Screening steps 3-8 must be performed within 90 days of the planned enrollment into the study. Should this screening window be exceeded before the first vaccination, screening procedures (not including administration of the informed consent form or comprehension questionnaire) may be repeated to ensure continued eligibility for the study (screening procedures can be repeated a maximum of one time).

Any clinically relevant finding that is discovered upon screening will be treated appropriately according to the standard of care in Brazil, as follows. Initial management will be performed at the study clinic, free of charge. Should referral for more extensive investigation or treatment be required, the study will arrange and pay for transportation to a government healthcare facility and initial consultation. Initial care – according to the standard of care in Brazil – will be covered; however, in the event that a chronic illness is discovered during the course of screening, long-term treatment and care will not be reimbursed by the study, but the individual will be referred to the local health authorities for appropriate treatment and follow-up.

If the fecal exam performed during screening demonstrates infection with an intestinal parasite the individual will be treated with an appropriate medication prior to being enrolled in the study and before any vaccinations. Volunteers in Part I of the study will be excluded if infected with hookworm at screening. Individuals infected with *Ascaris lumbricoides* or *Trichuris trichiura* will be treated with a single 400 mg oral dose of albendazole; three 400 mg doses of albendazole will be given to individuals infected with hookworm; individuals infected with *S. mansoni* or *Taenia* spp. will be treated with a single 60 mg/kg oral dose of praziquantel; and, individuals

infected with *Strongyloides stercoralis* will be treated with three 400 mg daily oral doses of albendazole.

7.3 Randomization Process for Part II of the Study

For Part II of the study, the eligible volunteers assigned to Cohorts 7 to 12 will be asked to come to the study clinic on their scheduled day of enrollment into the study; transportation to the clinic will be provided. After undergoing a clinical interview and exam to ensure that they remain eligible for participation in the study, that they have had blood collected for the studies outlined in **Section 7.6**, and that females have had a urine pregnancy test performed that is documented to be negative, volunteers will be assigned a unique study number and vaccinated as described in **Section 7.5**.

Randomization to either *Na*-GST-1/Alhydrogel®, *Na*-GST-1/Alhydrogel®/GLA-AF or Butang® will be done through use of a randomization code furnished to the study vaccine manager by the data-management center. The study numbers will be assigned in the order in which the participants are enrolled in the study, so that among the first 10 study numbers in Part II of the study, 8 will be assigned to *Na*-GST-1/Alhydrogel and 2 to Butang®. Assignment of the study numbers will be done on the day of first vaccination, in the order that the study participants present for immunization.

Access to the randomization list will be exclusively limited to the study vaccine manager and their assistant. Between vaccination days, the randomization list will be stored in a sealed envelope in a locked cabinet. The study vaccine manager (and assistant) will be unblinded, but will not be involved in study participants' further evaluation.

7.4 Enrollment

Volunteers will not be considered enrolled in the study until they have received their first dose of vaccine. In the event that a volunteer is randomized but not enrolled on the day of first vaccination, they will be replaced with an eligible alternate.

7.5 Immunization Procedure

Participants will receive 3 immunizations, on Days 0, 56, and 112. *Na*-GST-1/Alhydrogel®, GLA-AF and Butang® supplies will be kept refrigerated at 2°C to 8°C until just before use, whereupon they will be warmed to room temperature. The *Na*-GST-1/Alhydrogel® formulations (10, 30 and 100 µg doses of *Na*-GST-1 administered with or without GLA-AF) and Butang® will be delivered by IM injection in the deltoid muscle with a single-use needle of appropriate length after preparation of the site with alcohol. Successive vaccinations will be given in alternating arms.

7.6 Clinical Monitoring and Evaluation

See **Appendix B** for a tabular representation of study procedures.

<u>Study Day 0 (Day of First Vaccination)</u>

1. Verify that Informed Consent was obtained.
2. Verify that all applicable eligibility criteria have been met.

3. Perform abbreviated history (including concomitant medications) and physical exam, focusing on any acute complaints.
4. Obtain blood for hematology, biochemistry, anti-GST-1 and total IgE antibody ELISAs and cellular immunology assays.
5. For females, obtain a urine sample for hCG testing. Ensure that test is negative before proceeding; a positive test will exclude the volunteer from the trial.
6. Record vital signs (blood pressure, oral temperature, and heart rate).
7. Administer the vaccine.
8. Observe for at least 2 hours after vaccination to evaluate for immediate adverse reactions. During the 2-hour post-immunization wait period, study staff will discuss signs and symptoms of potential AEs.

Study Day 1
1. Perform basic history and physical exam (including injection site), emphasizing examination of any acute complaints.
2. Record vital signs.

Study Day 3 +/- 1
1. Perform basic history and physical exam (including injection site), emphasizing examination of any acute complaints.
2. Record vital signs.

Study Day 7 +/- 1
1. Perform basic history and physical exam (including injection site), emphasizing examination of any acute complaints.
2. Record vital signs.

Study Day 14 +/- 2
1. Perform basic history and physical exam (including injection site), emphasizing examination of any acute complaints.
2. Record vital signs.
3. Obtain blood for hematology, biochemistry, anti-GST-1 and total IgE antibody ELISAs, and cellular assays.

Study Day 28 +/- 4
1. Perform basic history and physical exam (including injection site), emphasizing examination of any acute complaints.
2. Record vital signs.
3. Obtain blood for anti-GST-1 and total IgE antibody ELISAs and cellular assays.

Study Day 56 +/- 7 (Day of Second Vaccination)
1. Perform basic history (including concomitant medications) and physical exam (including injection site), emphasizing examination of any acute complaints.
2. Obtain blood for hematology, biochemistry, anti-GST-1 and total IgE antibody ELISAs, and cellular immunology assays.

3. For females, obtain a urine sample for hCG testing. Ensure that test is negative before proceeding; a positive test will exclude the volunteer from further vaccinations.
4. Record vital signs.
5. Administer the vaccine.
6. Observe for at least 2 hours after vaccination to evaluate for immediate adverse reactions. During the 2-hour post-immunization wait period, study staff will discuss signs and symptoms of potential AEs.

<u>Study Day 57 (1 day after Second Vaccination)</u>
1. Perform basic history and physical exam (including injection site), emphasizing examination of any acute complaints.
2. Record vital signs.

<u>Study Day 59 (3 +/- 1 days after Second Vaccination)</u>
1. Perform basic history and physical exam (including injection site), emphasizing examination of any acute complaints.
2. Record vital signs.

<u>Study Day 63 (7 +/- 1 days after Second Vaccination)</u>
1. Perform basic history and physical exam (including injection site), emphasizing examination of any acute complaints.
2. Record vital signs.

<u>Study Day 70 (14 +/- 2 days after Second Vaccination)</u>
1. Perform basic history and physical exam (including injection site), emphasizing examination of any acute complaints.
2. Record vital signs.
3. Obtain blood for hematology, biochemistry, anti-GST-1 and total IgE antibody ELISAs, and cellular immunology assays.

<u>Study Day 84 (28 +/- 4 days after Second Vaccination)</u>
1. Perform basic history and physical exam, emphasizing examination of any complaints.
2. Record vital signs.
3. Obtain blood for anti-GST-1 and total IgE antibody ELISAs and cellular assays.

<u>Study Day 112 +/- 14 (Day of Third Vaccination)</u>
1. Perform basic history (including concomitant medications) and physical exam (including injection site), emphasizing examination of any acute complaints.
2. Obtain blood for hematology, biochemistry, anti-GST-1 and total IgE antibody ELISAs, and cellular immunology assays.
3. For females, obtain a urine sample for hCG testing. Ensure that test is negative before proceeding; a positive test will exclude the volunteer from further vaccinations.
4. Record vital signs.
5. Administer the vaccine.

6. Observe for at least 2 hours after vaccination to evaluate for immediate adverse reactions. During the 2-hour post-immunization wait period, study staff will discuss signs and symptoms of potential AEs.

Study Day 113 (1 day after Third Vaccination)
1. Perform basic history and physical exam (including injection site), emphasizing examination of any acute complaints.
2. Record vital signs.

Study Day 115 (3 +/- 1 days after Third Vaccination)
1. Perform basic history and physical exam (including injection site), emphasizing examination of any acute complaints.
2. Record vital signs.

Study Day 119 (7 +/- 1 days after Third Vaccination)
1. Perform basic history and physical exam (including injection site), emphasizing examination of any acute complaints.
2. Record vital signs.

Study Day 126 (14 +/- 2 days after Third Vaccination)
1. Perform basic history and physical exam (including injection site), emphasizing examination of any acute complaints.
2. Record vital signs.
3. Obtain blood for hematology, biochemistry, anti-GST-1 and total IgE antibody ELISAs, and cellular immunology assays.

Study Day 140 (28 +/-4 days after Third Vaccination)
1. Perform basic history and physical exam, emphasizing examination of any acute complaints.
2. Obtain blood for anti-GST-1 and total IgE antibody ELISAs and cellular immunology assays.
3. Obtain stool for examination for ova and parasites (Part II only).

Study Day 200 (3 months +/-14 days after Third Vaccination)
1. Perform basic history and physical exam, emphasizing examination of any acute complaints.
2. Obtain blood for anti-GST-1 and total IgE antibody ELISAs and cellular immunology assays.

Study Day 290 (6 months +/-14 days after Third Vaccination)
1. Perform basic history and physical exam, emphasizing examination of any acute complaints.
2. Obtain blood for anti-GST-1 and total IgE antibody ELISAs and cellular immunology assays.

Study Day 380 (9 months +/-21 days after Third Vaccination)
1. Perform basic history and physical exam, emphasizing examination of any acute complaints.
2. Obtain blood for anti-GST-1 and total IgE antibody ELISAs.
3. Obtain stool for examination for ova and parasites (Part II only).

Study Day 470 (12 months +/- 21 days after Third Vaccination)

1. Perform basic history and physical exam, emphasizing examination of any acute complaints.

7.7 Laboratory Testing

Using standard techniques, the following tests will be performed at the Humberto Abrão clinical laboratory in Belo Horizonte, Minas Gerais, Brazil, in Part I of the study or the Carlos Chagas clinical laboratory in Governador Valadares, Minas Gerais, Brazil, for Part II of the study.

1. Complete blood count plus white blood cell (WBC) differential*
2. Serum creatinine
3. Alanine aminotransferase (ALT)
4. Serum glucose (random)
5. HBsAg ELISA
6. HCV ELISA
7. HIV ELISA
8. Prothrombin time - International Normalized Ratio (PT INR) [Part I only]
9. Partial thromboplastin time (PTT) [Part I only]

Urine hCG testing will be performed at the clinical trial sites using urine pregnancy test kits that have been approved by Brazilian and/or US regulatory agencies. Urine dipstick testing will also be performed at the trial sites using an approved product.

For Part II of the study, PT INR testing will be performed at the study site in Americaninhas using an FDA-approved portable, point-of-care instrument. Due to the distance between the field site and the Carlos Chagas clinical laboratory (approximately 4-5 hours' drive), it will not be possible to send blood collected from volunteers to this laboratory for PTT and PT/INR testing within the acceptable time limit.

Anti-*Na*-GST-1 ELISAs will be performed at FIOCRUZ in Belo Horizonte, Brazil, as will the exploratory immunology assays.

*The following CBC parameters will be assessed for safety throughout the trial: WBC, absolute neutrophil count, hemoglobin concentration and platelet count.

7.8 Immunologic Testing

*7.8.1 Anti-*Na*-GST-1 Antibody Assays*

Antibodies to *Na*-GST-1 will be measured in serum or plasma of study subjects using an indirect ELISA and/or a custom ImmunoCAP (Phadia, Inc.) assay. Antigen-specific IgG and IgE antibody levels will be measured by both methods at baseline and at several time-points post-vaccination; in addition, IgG subclasses will be measured by ELISA. For the ELISA method, microwell plates will be coated with purified recombinant *Na*-GST-1, blocked, and incubated with serial dilutions of a positive "standard reference serum" pool to generate a reference curve from which antibody levels of test sera can be interpolated. After washing, a horseradish peroxidase conjugated goat anti-human antibody will be added and incubated with plates,

washed again, incubated with a chromogenic substrate, and antibody levels measured on an ELISA plate reader.

Serum samples with anti-*Na*-GST-1 IgE results by ELISA that are greater than the reactivity limit will then be tested by the ImmunoCAP method. The ImmunoCAP method is considered to be the gold standard for measuring levels of antigen-specific IgE in serum. These tests will be performed at the Dermatology, Allergy, and Immunology Reference Laboratory of Johns Hopkins University (Baltimore, Maryland, US).

7.8.2 Western Blots

SDS-PAGE of recombinant *Na*-GST-1 followed by Western Blots with study participant's sera, and positive and negative control sera, will be performed.

7.8.3 Total IgE

Total IgE levels will be determined by use of a "sandwich" ELISA. Calibrators with specified IgE concentrations traceable to World Health Organization (WHO) reference preparations will be run simultaneously with the samples, and this calibration curve will provide the basis for calculating the IgE concentration in each test sample.

7.8.4 Cellular Immunology Assays

Lymphocyte proliferation assays will be performed using blood collected at various time points in the study, by separating peripheral blood mononuclear cells (PBMCs) from whole blood and culturing cells with media only, the mitogen phytohemagglutinin, or the antigen *Na*-GST-1. At the end of the culture period, the cells will be pulsed with [^{3}H]-thymidine, harvested, and the incorporated thymidine level detected using a beta-counter. Stimulation indices will be calculated and compared to a control consisting only of media.

Cytokines (e.g., IL-2, IL-4, IL-5, IL-10, TNFα, and IFNγ) will be quantified using Cytometric Bead Analysis kits of supernatants collected from PBMCs stimulated with *Na*-GST-1 antigen for 48 and 72 hours *in vitro*. Chemokines (e.g., RANTES, MIP1a, MCP-1, and IP-10) will be quantified in supernatants using commercially available ELISA kits. Flow cytometric analysis of cell surface markers (phenotyping) of whole blood will also be performed to evaluate the frequency and distribution of different cell types.

7.8.5 Storage of Participant Specimens

Extra samples of serum, plasma, and feces will be stored for at least five years, in case future questions arise regarding the safety or immune response to the vaccine. Extra lymphocytes will be cryopreserved for future development of human monoclonal antibodies to be used for future immunoassays. Prior to conducting any future tests on stored samples that are not described in this protocol, permission will first be obtained from the IRBs.

7.9 Stool Examination

Fecal exams will begin with an ether sedimentation assay to determine infection status (positive or negative). In Part II of the study, positive samples will undergo Kato Katz fecal thick smear exams to determine infection intensity (eggs per gram of feces).

8 ADVERSE EVENTS MONITORING AND REPORTING

8.1 Definitions

8.1.1 Adverse Event

An adverse event (AE) includes any noxious, pathological or unintended change in anatomical, physiological or metabolic functions as indicated by physical signs, symptoms and/or laboratory-detected changes occurring in any phase of the clinical study, whether associated with the study vaccine or active comparator, and whether or not considered vaccination related. This includes an exacerbation of pre-existing conditions and intercurrent illnesses. All AEs must be graded for severity and relationship to the study vaccine as described in **Section 8.2.2** and **Section 8.2.3** in this protocol.

8.1.2 Serious Adverse Event (SAE)

An SAE is an AE, whether considered related to the study vaccine or not, meeting one of the following conditions:

1. Death during the period of protocol-defined surveillance
2. Life threatening: defined as an event that places a subject at immediate risk of death at the time of the event and does not refer to an event that hypothetically might have caused death were it more severe
3. Hospitalization during the period of protocol-defined surveillance: defined as at least an overnight stay in the hospital or emergency ward for treatment that would have been inappropriate if administered in the outpatient setting
4. Results in a congenital anomaly or birth defect
5. Results in a persistent or significant disability or incapacity: defined as a substantial disruption of the study participant's ability to carry out normal life functions
6. Any other important medical event that may not result in death, be life threatening, or require hospitalization, may be considered a serious AE when, based upon appropriate medical judgment, the event may jeopardize the subject and may require medical or surgical intervention to prevent one of the outcomes listed above.

8.1.3 Adverse Events of Special Interest (AESIs)

Special attention will be paid to monitoring for the occurrence of certain adverse events termed, "Adverse Events of Special Interest" or AESIs. These include inflammatory and autoimmune disorders that may potentially be related to the use of an immunostimulatory adjuvant (although none have been associated with the use of GLA-AF). The occurrence of the following AESI's will be closely monitored:

- Neuroinflammatory disorders (optic neuritis, multiple sclerosis, demyelinating disease, transverse myelitis, Guillain-Barre syndrome, myasthenia gravis, encephalitis, neuritis, Bell's palsy)
- Musculoskeletal disorders (systemic lupus erythematosus, cutaneous lupus, Sjogren's syndrome, scleroderma, dermatomyositis, polymyositis, rheumatoid arthritis, juvenile

rheumatoid arthritis, polymyalgia, rheumatica, reactive arthritis, psoriatic arthropathy, ankylosing spondylitis, spondylarthropathy)

- Gastrointestinal disorders (Crohn's disease, ulcerative colitis, celiac disease)
- Metabolic diseases (autoimmune thyroiditis, Grave's or Basedow's disease, Hashimoto thyroiditis, insulin-dependent diabetes mellitus, Addison's disease)
- Skin disorders (psoriasis, vitiligo, Raynaud's phenomenon, erythema nodosum, autoimmune bullous skin diseases)
- Others (autoimmune hemolytic anemia, idiopathic thrombocytopenic purpura, antiphospholipid syndrome, temporal arteritis, Behcet's syndrome, pernicious anemia, autoimmune hepatitis, primary biliary cirrhosis, primary sclerosing cholangitis, autoimmune glomerulonephritis, autoimmune uveitis, autoimmune cardiomyopathy, sarcoidosis, Stevens-Johnson syndrome).
- Vasculitides

8.2 Assessment of Adverse Events

8.2.1 Identification of AEs

Assessment of safety will include clinical observation and monitoring of hematological, blood chemistry, and immunologic parameters. Safety will be evaluated by monitoring of participants for local and systemic adverse reactions during the course of the trial. Participants will be closely monitored for at least 2 hours following each immunization. Additionally, participants will be evaluated on Days 1, 3, 7, 14 and 28 following each vaccination for clinical assessments.

All AEs will be graded for severity and relationship to study vaccine. Reactions will be graded as described in **Section 8.2.2** in this protocol. During Part II of the study, a study clinician will stay in or near Americaninhas for the duration of the trial and will be available to the study participants at all times. Should a participant call on a study clinician to report an AE, it will be fully documented in their study chart, and discussed with the Principal Investigator.

8.2.2 Determination of Severity

All AEs will be assessed by the investigator using the following protocol-defined grading system:

Grade 0:	None
Grade 1:	Mild - No effect on activities of daily living; no medical intervention/therapy required
Grade 2:	Moderate - Partial limitation in activities of daily living (can complete $\geq$50% of baseline); no or minimal medical intervention/therapy required
Grade 3:	Severe - Activities of daily living limited to < 50% of baseline; medical evaluation/therapy required
Grade 4:	Potentially life threatening

Intensity of the following AEs will be assessed by the investigator as described in **Table 4**. All laboratory AEs will be graded in severity following the toxicity table in **Appendix C**. Unsolicited adverse events and clinically significant laboratory abnormalities not described in **Table 4** or **Appendix C** will be graded according to **Appendix D**.

Table 4: Assessment of Adverse Event Severity

Adverse Event	Grade	Severity
Pain at injection site	0	None
	1	Easily tolerated, does not interfere with activity
	2	Repeated use of non-narcotic pain reliever for > 24 hours or interferes with daily activity
	3	Any use of narcotic pain reliever or prevents daily activity
	4	Emergency room (ER) visit or hospitalization
Tenderness at injection site	0	None
	1	Mild discomfort to touch
	2	Discomfort with movement
	3	Significant discomfort at rest
	4	ER visit or hospitalization
Erythema at injection site	0	< 25 mm
	1	25 mm - 50 mm
	2	51 mm - 100 mm
	3	> 100 mm
	4	Necrosis or exfoliative dermatitis
Induration/swelling at injection site	0	<25 mm
	1	25 mm - 50 mm and does not interfere with daily activity
	2	51 mm - 100 mm or interferes with daily activity
	3	>100 mm or prevents daily activity
	4	Necrosis
Fever (oral)	0	<38.0°C
	1	38.0°C - 38.4°C
	2	38.5°C - 38.9°C
	3	39.0°C – 40.0°C
	4	>40.0°C
Headache	0	None
	1	Easily tolerated, does not interfere with daily activity
	2	Repeated use of non-narcotic pain reliever for >24 hours or interferes with daily activity
	3	Any use of narcotic pain reliever or prevents daily activity
	4	ER visit or hospitalization
Nausea	0	None
	1	Easily tolerated, does not interfere with daily activity
	2	Interferes with daily activity
	3	Prevents daily activity
	4	ER visit or hospitalization
Vomiting	0	None
	1	1-2 episodes in 24 hours and does not interfere with activity
	2	> 2 episodes in 24 hours or interferes with daily activity
	3	Prevents daily activity or requires outpatient IV hydration
	4	ER visit or hospitalization for hypotensive shock
Myalgia	0	None
	1	Easily tolerated, does not interfere with daily activity
	2	Interferes with daily activity
	3	Prevents daily activity
	4	ER visit or hospitalization
Arthralgia	0	None
	1	Easily tolerated, does not interfere with activity
	2	Interferes with daily activity
	3	Prevents daily activity
	4	ER visit or hospitalization

Urticaria	0<br>1<br>2<br><br>3<br>4	None<br>Requiring no medications<br>Requiring PO or topical treatment, or IV medication or steroids for <24 hours<br>Requiring IV medication or steroids for >24 hours<br>ER visit or hospitalization
Mucocutaneous Reaction/Rash	0<br>1<br>2<br>3<br>4	None<br>Erythema; pruritus or localized macular rash<br>Diffuse, maculo-papular rash, dry desquamation<br>Vesiculation or moist desquamation or ulceration<br>Necrosis

8.2.3 Association with Receipt of the Study Vaccine

All AEs will have their possible relationship to study vaccine assessed using the following terms:

Definite: Clear-cut temporal association, and no other possible cause.
Probable: Clear-cut temporal association and a potential alternative etiology is not apparent.
Possible: Less clear temporal association; other etiologies also possible.
Unlikely: Temporal association between the AE and the vaccine or the nature of the event is such that the vaccine is not likely to have had any reasonable association with the observed illness/event (cause and effect relationship improbable but not impossible).
Not Related: The AE is completely independent of vaccine administration; and/or evidence exists that the event is definitely related to another etiology.

The degree of certainty with which an AE can be attributed to administration of the study vaccine will be determined by how well the event can be understood in terms of one or more of the following:

1. The event being temporally related with vaccination or reproduced on re-vaccination.
2. A reaction of similar nature having previously been observed with this type of vaccine and/or formulation.
3. The event having often been reported in the literature for similar types of vaccines.

All local (injection-site) reactions will be considered definitely related to vaccination.

8.3 Adverse Event Reporting

All SAEs will be reviewed by a study physician, recorded on the appropriate SAE form, and followed through to resolution or stabilization by a study physician. All SAEs will be reported by email, telephone or fax within 1 working day of notification of the SAE occurrence to the Principal Investigator, to all of the following:

- Sponsor (Office of Vaccine Operations, Sabin Vaccine Institute): Phone: 202-842-5025, Fax: 202-842-7689
- Centro de Pesquisa René Rachou Ethics Committee: Phone: +55-31-3349-7825, Fax: +55-31-3295-3115
- GWUMC Institutional Review Board (IRB): Phone: 202-994-2715, Fax: 202-994-0247

Following notification from the investigator, SVI as the sponsor of the investigational product, will report events that are both serious and unexpected that are possibly, probably, or definitely related to the vaccine, to the FDA and ANVISA within the required timelines: fatal and life-threatening events within 7 calendar days (by phone, fax, or internet) and all other SAEs in writing within 15 calendar days. All SAEs not listed as possibly, probably, or definitely related will be reported to the FDA and ANVISA at least annually in a summary format.

All AESIs will be reported to Sponsor and to the FDA and ANVISA according to the same procedure as for reporting SAEs, and according to the same timelines as described above.

All local and systemic reactions not meeting the criteria for "serious adverse events" will be captured on the appropriate case report form (CRF). These events will be followed to resolution. Grade 3 adverse events deemed definitely or probably related to vaccination will be reported by email or fax within 15 working days of the Principal Investigator becoming aware of the event, to the Sponsor and the Safety Monitoring Committee.

8.4 Adverse Event Monitoring

8.4.1 Medical Monitor

An independent medical monitor will be appointed for oversight of participant safety in this trial. The Monitor for Part I of the study may be different than the one for Part II, due to the distance between the sites. The Medical Monitor(s) will be available to advise the investigators on trial-related medical questions or problems. Should the Monitor(s) not be available, they will recommend an alternative to serve as a substitute independent Medical Monitor.

The Medical Monitor's primary responsibility will be to monitor participant safety. The Principal Investigator is responsible for ensuring that the Medical Monitor is aware of any new safety information that becomes available during the course of the trial.

8.4.2 Safety Monitoring Committee (SMC)

SVI will select at least three individuals (one of whom will be the independent Medical Monitor) to advise SVI and the study investigators on the trial. These individuals will be independent from SVI and the Human Hookworm Vaccine Initiative (HHVI). Individuals may be affiliated with GWU or FIOCRUZ if their affiliation is unrelated to the SVI Product Development Partnership and HHVI. The SMC's primary responsibility will be to monitor participant safety. The Principal Investigator is responsible for ensuring that the SMC is aware of all new safety information. The SMC will periodically review data on safety and enrollment, and will review cumulative safety data for evidence of study related AEs, adherence to the protocol, and factors that may affect outcome or study data such as protocol violations and losses to follow up.

All cumulative safety data reports from the trial will be submitted to the SMC before beginning vaccinations in Part II of the study as well as before each dose escalation in Part II (i.e., between the first dose in Cohort 7 and Cohort 9 and the first dose in Cohort 9 and Cohort 11, and so on). Safety data reports will include data from at least the first 7 days after vaccination. After the third and final vaccination has been administered to all dose cohorts, additional safety and immunology results and reports will be submitted to the SMC as they become available. A final report will be submitted to the SMC following completion of the study.

Conference calls between the investigators and the SMC will be scheduled at least one week prior to beginning Part II of the study and prior to each dose escalation in Part II (i.e., after the first dose is administered to Cohort 7 but before the first dose is administered to Cohort 9, etc.). If no criteria for suspending the study are met (see **Section 8.5**), vaccinations will proceed with approval from the SMC. During Part II of the study, should the SMC not be able to meet in a timely fashion, the SMC may reserve the right to defer to the independent Medical Monitor to decide whether or not dose escalation may proceed.

Written approval (via fax or email) to proceed to Part II of the study or to the next dose concentration of vaccine must be obtained from the SMC (or Medical Monitor during Part II in the event that the SMC cannot review the safety data in a timely manner, as described above) prior to administration. Both the SMC and Medical Monitor will have access to the randomization code for Part II of the study, as they may wish to review the data in an unblinded fashion should significant safety questions arise prior to the final unblinding.

It is the Principal Investigator's (or designated agent) responsibility to ensure that the SMC reviews the current safety data (grouped by dose cohort), study protocol, and any other requested documents at its meetings. Occurrence of an SAE will be reported to the SMC at the same time that it is reported to the IRBs/IECs. Additionally, any new information that may adversely affect the safety of the subjects or the conduct of the study will be submitted to the SMC as it becomes available.

8.5 Criteria for Suspension of Further Injections

If a dose of vaccine is considered significantly reactogenic (see below), dose escalation and/or additional vaccinations will be suspended until reviewed by the Medical Monitor, SMC and study sponsor (SVI). Any recommendation of the Medical Monitor and SMC to resume or suspend further injections (either for an individual participant, an entire dose cohort, or all dose cohorts) will be communicated in writing to the sponsor and Principal Investigator. All communications from the SMC will subsequently be forwarded by the investigators to the CPqRR and GWUMC IRBs.

The following criteria will be used to define significant reactogenicity (note that in Part II of the study, these apply to both the *Na*-GST-1 vaccines and Butang® vaccine, as these criteria will be applied in advance of any consideration of unblinding):

a. One or more participants experience an SAE (as defined in **Section 8.1.2** in this protocol) that is determined to be possibly, probably, or definitely related to the vaccine (as defined in **Section 8.2.3** in this protocol), **OR**
b. One or more participants experience a hypersensitivity reaction (a Grade 3 allergic reaction, as defined in **Appendix D**) that is possibly, probably or definitely related to the vaccine, **OR**
c. One or more participants in a single dose cohort experience an objective physical finding or laboratory abnormality of Grade 3 or higher (with the exception of isolated Grade 3 erythema or swelling), as defined in **Section 8.2.2** in this protocol, that is determined to be probably or definitely related to the vaccine. **OR**

d. Two or more participants in a single dose cohort experience a Grade 2 or higher safety laboratory abnormality or Grade 3 clinical AE that is possibly, probably or definitely related to the vaccine.

8.6 Treatments That Could Potentially Interfere with Vaccine-Induced Immunity

The following criteria will be checked at each visit. If any are reported during the study, the participant will be excluded from receiving further doses of the study vaccine and will not be included in the immunogenicity evaluations after the time of exclusion. The participant will, however, be encouraged to remain in the safety evaluation for doses already received.

1. Use of any investigational drug or investigational vaccine other than the study vaccine during the study period.
2. Administration of chronic (defined as more than 14 days) immunosuppressants or other immune-modifying drugs up to 30 days after the last dose of vaccine. (Topical and nasal steroids are allowed.)
3. Administration of immunoglobulins and/or any blood products up to 30 days after the last dose of vaccine.

8.7 Criteria for Suspension of Injections

The following criteria will be checked prior to each immunization and are contraindications to further immunization. However, the participant will be encouraged to remain in the safety evaluation for doses already received.

1. Hypersensitivity reaction following administration of a study vaccine.
2. Pregnancy, as determined by a positive urine β-hCG.
3. Occurrence of any other condition, which in the view of the investigators or Medical Monitor could jeopardize the safety of the study participant or may complicate interpretation of the safety or immunogenicity data.

8.8 Criteria for Deferral of Injections

The following adverse events constitute grounds for deferral of vaccine administration at that point in time; if any one of these occurs at the time scheduled for vaccination, the participant may be vaccinated at a later date, within the allowable time interval specified in **Section 7.6** of this protocol, or withdrawn at the discretion of the investigator. The participant must be followed until resolution or stabilization of the event as with any AE. If the participant is withdrawn from the study, he/she will be encouraged to remain in the safety evaluation for the duration of the study.

1. Oral temperature > 38.0°C at the time of vaccination will warrant deferral of immunization until fever and symptoms resolve.
2. Any other acute condition that in the opinion of the investigator poses a threat to the individual if immunized or that may complicate interpretation of the safety of the vaccine following immunization.

Such individual(s) will be followed until the symptoms resolve or the window for immunization expires. No further vaccination will be performed if the participant does not recover (oral temperature ≤ 38.0°C and/or lack of symptoms) within the originally scheduled vaccination time interval. The participant, however, will be followed for safety and immunogenicity evaluations. If the individual meets any of the above criteria for deferral on the day of first immunization, as an alternative to deferral of vaccination, the investigator may instead elect to exclude the participant from enrollment in the study. Eligible alternates will then be vaccinated instead.

8.9 Criteria for Withdrawal of an Individual Participant from the Study

A participant will not be considered to have completed the trial if any of the following reasons apply. However, any participant who has received at least one dose of vaccine will be encouraged to remain in the safety evaluation for the duration of the study. Should a female participant become pregnant during the course of the study, she will not receive further doses of vaccine, but will be followed for the duration of the pregnancy.

1. *Research terminated by sponsor or investigator* – applies to the situation where the entire study is terminated by the sponsor or investigator for any reason.
2. *Withdrawal of consent* – applies to a participant who withdraws consent to participate in the study for any reason.
3. *Noncompliant with protocol* – applies to a participant who does not comply with protocol-specific visits or evaluations, on a consistent basis, such that adequate follow-up is not possible and the participant's safety would be compromised by continuing in the trial. Additionally, this applies to a participant who is lost to follow-up and cannot be located.
4. *Develops an adverse event* - applies to a participant who is withdrawn from the study due to an adverse event, serious or otherwise.
5. *Other* – is a category used when previous categories do not apply, and requires an explanation.

8.10 Breaking the Study Blind

As outlined in **Section 4.4**, Part II of the study will be double-blinded until all study participants have completed their Day 140 visit, after which the investigators will be unblinded. During the double-blinded part of the study, a study participant's randomization code may be unblinded only for safety purposes. This is very unlikely to occur, as once a vaccine is administered, knowing which vaccine was given is unlikely to influence the medical management of an AE. This procedure is therefore exceptional and any decision to unblind will be discussed with the sponsor, the Principal Investigator, the Medical Monitor, and the SMC. If deemed necessary for urgent safety reasons, the Medical Monitor, in consultation with the SMC (if possible in a timely manner), may unblind a specific participant without revealing the study blind to the investigators or the Sponsor. Any unblinding will be thoroughly documented. It is to be emphasized that the Medical Monitor may put the study on hold at any time and discuss with the SMC. The decision to completely unblind or permanently stop the study prior to Day 140 will take the form of a formal recommendation by the SMC to the study Sponsor. The Principal Investigator must then notify the IRBs of this decision.

In the event that the investigators come to know the study code prior to final unblinding, the Principal Investigator must notify the Sponsor immediately. The reasons will be documented by the Principal Investigator and added to the study file.

Unblinding of the investigators will be done after all study participants have completed the Day 140 visit and upon authorization of the Sponsor. This will take place only after monitoring and verification of cGCP compliance by SVI, and after all safety and primary immunological results (i.e., antigen-specific IgG antibody levels as determined by ELISA) have been entered and the databases locked.

9 Data Collection and Monitoring

9.1 Source Documentation and Case Report Forms

Complete source documentation (clinical evaluations and test results) will be collected for every study participant for the duration of the study. In addition, supplementary documents (laboratory test reports, supplementary hospital or medical records, etc.) may form part of the source documentation for a study participant. CRFs will be used to record study-specific data for enrolled participants. The Principal Investigator is responsible for the accuracy and completeness of the data reported in the CRFs and the source documents. Data reported in the CRFs that is derived from source documents should be consistent with source documents and the discrepancies should be explained.

9.2 Study Documentation

Study-related documentation will be completed as required by the IRBs, the sponsor, and regulatory authorities. Continuing review documentation will be submitted by the Principal Investigator to the IRBs on the anniversary date of initial review as specified by each IRB. An annual report will be submitted by the sponsor to the FDA within 60 days of the anniversary date that the IND for *Na*-GST-1/Alhydrogel® was submitted.

9.2.1 Study Reports

In addition to the study-related documentation required by the regulatory authorities, three reports will be generated. The first interim report will be completed after the safety data from the Day 70 visits of Cohorts 1-6 have been compiled, for the purposes of deciding whether it is safe to proceed with enrollment into **Part II** of the study. The second interim report will be completed after the safety and primary immunogenicity data from the Day 140 visit of Cohorts 7-12 have been compiled and the study investigators have been unblinded. This report will serve as the basis for deciding whether to continue with future phase 1 testing of the vaccine (and which formulation) in children, which is presently being planned for Brazil in 2015.

A final report containing all safety and immunology data will be prepared after trial completion.

9.3 Retention of Records

Trial-related documents will be maintained by the Investigator for a period of 2 years after final marketing approval of the vaccine, or if 2 years have elapsed since the formal discontinuation of clinical development of the product. The Sponsor is required to inform the Principal Investigator

as to when such documents need no longer be retained. Storage of all trial-related documents will be such that confidentiality will be strictly maintained.

9.4 Protocol Revisions

No revisions to this protocol will be permitted without documented approval from both the Sponsor and the IRBs that granted the original approval for the study. This does not apply to changes made to reduce discomfort or avert risk to study participants. Furthermore, in the event of a medical emergency, the investigators shall perform any medical procedures that are deemed medically appropriate. The Principal Investigator must notify the Sponsor of all such occurrences. Any change to the protocol will be submitted to the actively participating IRBs (GWUMC and CPqRR) as a protocol amendment, and changes not affecting risk to participants may be expedited, as appropriate.

9.5 Clinical Investigator's Brochure

Investigators will receive the current version of the Clinical Investigator's Brochure, which comprehensively describes all the available preclinical and human experience with the experimental vaccine. If relevant new information becomes available during the course of the trial, the investigators will receive the revised Investigator's Brochure.

9.6 Study Monitoring

The Sponsor (SVI or its designee) will monitor all aspects of the study, with respect to current Good Clinical Practices, and for compliance with applicable government regulations. Prior to the start of the study, the Principal Investigator will be informed of the frequency of monitoring visits and will be given reasonable notification prior to each visit. The objectives of a monitoring visit will be to verify the prompt reporting of SAEs, to check the availability of the signed Informed Consent for enrolled study participants, to compare CRFs and spreadsheets with source data for completeness and accuracy, to verify compliance with the clinical protocol, and to check investigational product accountability. During the monitoring visit, the Principal Investigator (and/or designee) and other study personnel should be available to discuss the study. Study documents must be available for review throughout the course of the study.

10 STATISTICAL CONSIDERATIONS

10.1 General Design

The goal of this Phase 1 vaccine trial is to assess the safety, reactogenicity, and immunogenicity of *Na*-GST-1/Alhydrogel® and *Na*-GST-1/Alhydrogel®/GLA-AF in healthy adults compared to the Butang® hepatitis B vaccine. These parameters will be assessed in adults without any history of infection with or exposure to hookworm, as well as individuals previously infected with hookworm.

10.1.1 Description of the Statistical Methods to Be Employed

The purpose of this trial is to estimate AE rates and patterns of immune response as well as to compare these rates and patterns between the investigational and comparator vaccines, in different doses of the *Na*-GST-1 formulations.

This section briefly describes the statistical methods to be used; a detailed analytical plan will fully describe the methods. The analytical plan will discuss the planned approaches to missing data. Deviations from the original analytical plan will be thoroughly documented and reported to the Sponsor.

Descriptive and hypothesis-testing approaches will be used to meet the protocol objectives as stated in **Section 2.0**. Estimates will be presented with their 95% confidence intervals. Formal statistical tests, as outlined below, will be used to compare doses. No formal adjustments for multiple comparisons will be made. Statistical tests will use a two-sided significance level of 5%.

Primary Objective: To estimate the frequency of vaccine-related AEs, graded by severity, for each dose and formulation of *Na*-GST-1.

AEs will be coded according to Medical Dictionary of Regulatory Activities (MedDRA™) preferred terms. The frequency, severity, and relationship of AEs for each vaccine formulation and dose cohort will be presented in tabular form using the MedDRA™ coded term and organized by MedDRA™ System, Organ, and Class (SOC) designations.

- The frequency of immediate, systemic, and local injection site AEs will be summarized by SOC and preferred term.
- A line listing of each clinical and laboratory AE classified as immediate (within the first 2 hours), systemic, and local will be displayed in tables stratified by vaccine allocation and dose cohort.
- AEs will be summarized by severity and relationship to vaccine formulation by individuals and dose of *Na*-GST-1.
- The frequency of vaccine-related SAEs will be tallied as well as summarized by body system, by vaccine formulation and dose cohort.

The proportion of participants with at least one injection site AE will be compared by vaccine formulation and by dose cohort. The incidence of AEs will be compared across cohorts and not within cohorts. Therefore, the data for all participants who receive the hepatitis B vaccine (8 in total) will be summarized and compared to those who receive 10 µg *Na*-GST-1/Alhydrogel® (n=10), to those who receive 10 µg *Na*-GST-1/Alhydrogel®/GLA-AF, and so on. By merging the AE data of all participants who receive the same vaccine (hepatitis B, *Na*-GST-1/Alhydrogel®, or *Na*-GST-1/Alhydrogel®/GLA-AF), even if the individuals are not in the same cohort, the strength of the comparisons will be increased.

Laboratory results (hematological and clinical chemistry) will be examined for trends over time and any clinically significant values for individuals will be reported.

Secondary Objective 1: To determine the dose that generates the highest anti-*Na*-GST-1 IgG antibody response at Day 126, as determined by an indirect enzyme-linked immunosorbent assay (ELISA).

a. The proportion of participants with detectable anti-*Na*-GST-1 responses will be summarized as a descriptive measure.

b. Geometric mean antibody responses will be compared between vaccine formulation and dose groups. Comparisons between groups will be made by a one-way analysis of variance (ANOVA) with pair-wise comparisons between dose cohorts made by contrasts. Data from participants receiving the same vaccine (e.g., hepatitis B, *Na*-GST-1/Alhydrogel®, or *Na*-GST-1/Alhydrogel®/GLA-AF) will be combined across cohorts to increase the strength of the comparisons.

Secondary Objective 2: To assess and compare the duration of antibody response to *Na*-GST-1.

a. Immunogenicity responses will be summarized by vaccine formulation and dose of *Na*-GST-1, over time.

b. Individual responses will be described over time and stratified by *Na*-GST-1 formulation and by dose of *Na*-GST-1.

c. Antibody responses will be measured at Days 0, 14, 28, 56, 70, 84, 112, 126, 140, 200, 290, and 380. To exploit the multiple measures of antibody within each subject, a longitudinal model may be built (if possible) to describe the antibody response over time. The model will explore if there are any differences between *Na*-GST-1/Alhydrogel®, *Na*-GST-1/Alhydrogel®/GLA-AF and Butang®, and explore dose effects. As described for Secondary Objective 2, data from participants receiving the same vaccine will be combined across cohorts.

Secondary Objective 3: To perform exploratory studies of the cellular immune responses to the *Na*-GST-1 antigen both before and after immunization.

The following assays will be performed to assess the cellular immune responses to vaccination with *Na*-GST-1:

a. Lymphocyte proliferative responses to *in vitro* stimulation with *Na*-GST-1.

b. Cytokine and chemokine production *in vitro* in response to stimulation with *Na*-GST-1.

c. Changes in *in vivo* PBMC subpopulations as determined by flow cytometry.

Summary statistics will be used to present these immunogenicity results. To assess the changes in lymphocyte proliferative responses over time, a generalized estimating equations model with robust standard errors will be constructed.

Should the study be terminated early (see **Section 8.5**), the investigative team will discuss with the SMC the reason for termination and determine which study questions can be addressed in an

unbiased manner with the available data. The available data will be analyzed and interpreted in light of early termination.

10.2 Sample Size

This Phase 1 trial is not powered to detect statistically significant differences between groups. Even though comparative statistics for the safety variables will be computed, the study will have low power to detect anything other than very large differences in the incidence of local injection site and systemic side effects between the vaccination groups. This is done weighing the need to detect any possible untoward reactions against the need to limit the number of volunteers involved for safety purposes. The sample size of 102 for this study is within the range commonly used in Phase 1 trials for the initial assessment of the safety, tolerance and immunogenicity of an investigational vaccine. Incorporation of a comparator vaccine will enable broad initial estimates of the incidence of local injection site and systemic side effects and of immune responses among vaccine recipients.

11 Protection of Human Subjects

11.1 Institutional Review Boards

The investigators will be responsible for obtaining IRB approvals for the study. Before the start of the study, the appropriate documents (including the protocol, Investigator's Brochure, and informed consent form) will be submitted to the IRBs. Full approval for the study will be obtained from the CPqRR and George Washington University Medical Center IRBs.

The study will be conducted according to the Declaration of Helsinki, the US Code of Federal Regulations (Protection of Human Subjects [21 CFR 50], Institutional Review Boards [21 CFR 56], and Obligations of Clinical Investigators [21 CFR 312]) and Brazilian Resolution Nº 196/96 on Research Involving Human Subjects.

Modifications to the protocol will not be implemented without prior written IRB approval except when necessary to eliminate immediate hazards to the participants. The IRBs will be informed by the Investigator of any new information that may adversely affect the safety of the subjects or the conduct of the study, an annual update and/or request for re-approval, and when the study has been completed.

11.2 Informed Consent

The principles of informed consent in the current edition of the Declaration of Helsinki will be implemented prior to any protocol-specified procedures being conducted. Informed consent will be obtained in accordance with US 21 CFR 50.25 and Brazilian Resolution Nº 196/96 on Research Involving Human Subjects.

Informed consent will be documented by the use of a written consent form approved by the IRBs. All relevant information will be provided in both oral and written form in a way that is understandable to the subject. Ample time and opportunity will be given for the participant to inquire about details of the study. Subjects unable to read will place an imprint of their finger/thumb in the place of a signature; in addition, an independent witness, who is not a

member of the study team, will sign the consent form to attest that the volunteer comprehended the contents.

The Principal Investigator (or the Investigator's designee) will explain the nature of the study and will inform the subject that participation is voluntary and that they can withdraw at any time. The volunteer will be informed about the study's purpose, goals, expected benefits and risks, and potential risks that are currently unforeseeable. They will be provided with a description of the procedures and an estimated duration of time that will be required to participate in the study, and they will be informed of alternatives to participation in the study. The volunteers will receive an explanation as to what medical treatments are available if injury occurs as a result of participation in the study and whom to contact in the event of a study-related injury. They will also be informed whom they should contact for answers to any questions relating to the study. The volunteer will be informed that his/her signature or fingerprint on the informed consent form indicates that he/she has decided to participate in the study having discussed the information presented.

The original signed informed consent form for each volunteer will be maintained as part of the volunteer's study records by the Principal Investigator. A copy of the informed consent form will be provided to every volunteer.

11.3 Risks

Risks to the participants are those associated with venipuncture and with immunization. These risks are outlined below.

Female participants will be cautioned of the unknown risk of the *Na*-GST-1/Alhydrogel® or *Na*-GST-1/GLA-AF vaccines to the fetus. Females of childbearing potential, unless surgically sterile (i.e., bilateral tubal ligation, bilateral oophorectomy, or complete hysterectomy), is at least 2 years postmenopausal, or practices abstinence, must use 2 effective methods of avoiding pregnancy (including oral, transdermal, or implanted contraceptives, intrauterine device, female condom with spermicide, diaphragm with spermicide, cervical cap, or use of a condom with spermicide by the sexual partner) until at least one month following the last immunization. Female participants will be counseled by a study clinician or nurse, or referred to the local health center for evaluation and institution of an appropriate contraceptive method.

11.3.1 Venipuncture

Risks occasionally associated with venipuncture include pain and bruising at the site of venipuncture, lightheadedness, and syncope (rarely).

11.3.2 Immunization with Na-GST-1/Alhydrogel®

Possible local vaccine reactions include pain, swelling, erythema, induration, transient limitation of limb movement, lymphadenopathy, or pruritus at the injection site. Local subcutaneous nodules, believed to be granulomatous reactions to aluminum hydroxide, have been observed with use of aluminum hydroxide-based adjuvants. Thus, most aluminum hydroxide-adsorbed vaccines are injected intramuscularly rather than subcutaneously. Systemic reactions such as fever, headache, malaise, myalgia, and joint pain, may also possibly occur. Immediate

hypersensitivity reactions including urticaria, anaphylaxis, or other IgE-mediated responses are possible as with any vaccine. As with any investigational vaccine, there is a theoretical possibility of risks about which we have no present knowledge. Participants will be informed of any such risks should further data become available.

11.3.3 Immunization with Na-*GST-1/Alhydrogel® plus GLA-AF*

The possible local vaccine reactions to be expected are similar to those of *Na*-GST-1/Alhydrogel® administered without GLA-AF. However, in a study of another vaccine administered with GLA-SE (a formulation similar to GLA-AF), the frequency and intensity of local reactions were somewhat greater than when the vaccine was administered without GLA-SE.

11.3.4 Immunization with Butang®

Recombinant hepatitis B vaccines have been administered to millions of people worldwide and have an established safety record. Adverse reactions may be local and include erythema, warmth, edema, induration with or without tenderness as well as urticaria, and ecchymosis.[38] Malaise, transient fever, hypotension, nausea, diarrhea, headache, and arthralgia may develop in some patients after the injection. An apparent hypersensitivity syndrome (serum-sickness-like) of delayed onset has been reported days to weeks after vaccination, including: arthralgia/arthritis (usually transient), fever, and dermatologic reactions such as urticaria, erythema multiforme, ecchymoses and erythema nodosum. On very rare occasions, non-fatal anaphylaxis has been reported following administration of recombinant hepatitis B vaccines.

11.4 Precautions taken to Minimize Risks

As outlined above, the participants will be monitored closely during their participation in this study. The study vaccines have been produced according to current Good Manufacturing Practices (cGMP). The vaccines will be administered by experienced investigators with drugs and equipment available for the treatment of anaphylaxis and other potential adverse reactions. All vaccine doses will be given by intramuscular injection to minimize injection site reactions such as pain. Furthermore, to reduce the likelihood of immediate-type allergic reactions to the *Na*-GST-1 vaccine formulations, all potential trial participants will be screened for detectable IgE to *Na*-GST-1; individuals with detectable IgE antibodies to the vaccine antigen by the ELISA assay that are confirmed to be >0.35 kU_A/L by the ImmunoCAP method as described in **Section 7.8**, will be excluded from participation in the study.

11.5 Benefits

Participants may not receive any direct benefit from participation in this study. It is hoped that information gained in this study will contribute to the development of a safe and effective hookworm vaccine. Participants who receive Butang® during the course of the study proper may receive the benefit of subsequent protection against exposure to the hepatitis B virus. These participants will be tested for antibodies to the hepatitis B surface antigen using serum collected during the Day 380 visit. If a participant does not have protective levels of anti-hepatitis B surface antigen antibodies, they will be offered a second series of the vaccine using the manufacturer-recommended 0, 1, and 6-month vaccination schedule. This will be offered after the final study visit and at no charge to the participant. Participants who receive either of the *Na*-

GST-1 formulations during the trial will be offered Butang® hepatitis B vaccine after the conclusion of the study, and thus may also receive this benefit.

All participants in Part II of the study will undergo a stool examination for ova and parasites at the Day 140 and Day 380 study visits. Those found to have a helminth infection at either of these study visits will be offered appropriate treatment at the conclusion of the study, free-of-charge.

Free medical treatment will be provided to all enrolled participants during the active immunization phase and the follow-up period. If the investigators judge that a participant requires hospitalization, transportation will be arranged and the medical management of the participant will be monitored by a study physician and the local Medical Monitor. Medical care for ailments not related to vaccination will not extend beyond the study period, but will be referred to the nearest government-run health clinic. Medical care for ailments related to vaccination will extend at least until the condition has resolved or stabilized (if a chronic condition).

11.6 Confidentiality

All study-related information will be stored securely at the study sites at CPqRR and Americaninhas. All participant information will be stored in locked file cabinets in areas with access limited to study staff. All laboratory specimens, reports, study data collection, and administrative forms will be identified by coded number only, to maintain participant confidentiality. All computer entry will be done by coded number only, and all local databases will be secured with password-protected access systems. Forms, lists, and any other listings that link participant ID numbers to other identifying information will be stored in a separate, locked file in an area with limited access.

Participants' study information will not be released without the written permission of the participant, except as necessary for monitoring by SVI and/or its designee, and the FDA or Brazilian regulatory authorities.

11.7 Compensation

Participants will not be given any monetary compensation. If participants incur any expenses to come to the clinic, their transportation expenses will be paid for by the study, as will any expenses related to meals that are justified due to the time spent travelling to or remaining in the study clinic.

11.8 Publication of Study Results

It is anticipated that results from this study will be published in peer-reviewed journals. If publication is sought, the identity of study participants or any easily traceable identifiers will not be revealed. Authorship issues will be discussed and agreed upon between the Sponsor and collaborating partners prior to submission for publication. Additionally, the results of the study will be communicated to both study participants and the community at large.

12 References

1. Ashford RW, Craig PS, Oppenheimer SJ. Polyparasitism on the Kenya coast. 1. Prevalence, and association between parasitic infections. Ann Trop Med Parasitol. 1992; **86**(6): 671-9.
2. de Silva NR, Brooker S, Hotez PJ, Montresor A, Engels D, Savioli L. Soil-transmitted helminth infections: updating the global picture. Trends Parasitol. 2003; **19**(12): 547-51.
3. Hotez PJ, Brooker S, Bethony JM, Bottazzi ME, Loukas A, Xiao S. Hookworm infection. N Engl J Med. 2004; **351**(8): 799-807.
4. Stoltzfus RJ, Chwaya HM, Montresor A, Albonico M, Savioli L, Tielsch JM. Malaria, hookworms and recent fever are related to anemia and iron status indicators in 0- to 5-y old Zanzibari children and these relationships change with age. J Nutr. 2000; **130**(7): 1724-33.
5. Stoltzfus RJ, Dreyfuss ML, Chwaya HM, Albonico M. Hookworm control as a strategy to prevent iron deficiency. Nutr Rev. 1997; **55**(6): 223-32.
6. Guyatt HL, Brooker S, Peshu N, Shulman CE. Hookworm and anaemia prevalence. Lancet. 2000; **356**(9247): 2101.
7. Brooker S, Bethony J, Hotez PJ. Human hookworm infection in the 21st century. Adv Parasitol. 2004; **58**: 197-288.
8. Brooker S, Peshu N, Warn PA, Mosobo M, Guyatt HL, Marsh K, et al. The epidemiology of hookworm infection and its contribution to anaemia among pre-school children on the Kenyan coast. Trans R Soc Trop Med Hyg. 1999; **93**(3): 240-6.
9. Bundy DA, Chan MS, Savioli L. Hookworm infection in pregnancy. Trans R Soc Trop Med Hyg. 1995; **89**(5): 521-2.
10. Stoltzfus RJ, Chwaya HM, Tielsch JM, Schulze KJ, Albonico M, Savioli L. Epidemiology of iron deficiency anemia in Zanzibari schoolchildren: the importance of hookworms. Am J Clin Nutr. 1997; **65**(1): 153-9.
11. Guyatt HL, Brooker S, Kihamia CM, Hall A, Bundy DA. Evaluation of efficacy of school-based anthelmintic treatments against anaemia in children in the United Republic of Tanzania. Bull World Health Organ. 2001; **79**(8): 695-703.
12. Sakti H, Nokes C, Hertanto WS, Hendratno S, Hall A, Bundy DA, et al. Evidence for an association between hookworm infection and cognitive function in Indonesian school children. Trop Med Int Health. 1999; **4**(5): 322-34.
13. Jardim-Botelho A, Raff S, Rodrigues Rde A, Hoffman HJ, Diemert DJ, Correa-Oliveira R, et al. Hookworm, Ascaris lumbricoides infection and polyparasitism associated with poor cognitive performance in Brazilian schoolchildren. Trop Med Int Health. 2008; **13**(8): 994-1004.
14. Bundy DA, Medley GF. Immuno-epidemiology of human geohelminthiasis: ecological and immunological determinants of worm burden. Parasitology. 1992; **104 Suppl**: S105-19.
15. Bethony J, Chen J, Lin S, Xiao S, Zhan B, Li S, et al. Emerging patterns of hookworm infection: influence of aging on the intensity of Necator infection in Hainan Province, People's Republic of China. Clin Infect Dis. 2002; **35**(11): 1336-44.
16. Sampathkumar V, Rajaratnam A. Prevalence of anaemia and hookworm infestation among adolescent girls in one rural block of TamilNadu. Indian J Matern Child Health. 1997; **8**(3-4): 73-5.
17. Utzinger J, Keiser J. Schistosomiasis and soil-transmitted helminthiasis: common drugs for treatment and control. Expert Opin Pharmacother. 2004; **5**(2): 263-85.
18. Bennett A, Guyatt H. Reducing intestinal nematode infection: efficacy of albendazole and mebendazole. Parasitol Today. 2000; **16**(2): 71-4.

19. Horton J. Albendazole: a review of anthelmintic efficacy and safety in humans. Parasitology. 2000; **121 Suppl**: S113-32.
20. Keiser J, Utzinger J. Efficacy of current drugs against soil-transmitted helminth infections: systematic review and meta-analysis. JAMA. 2008; **299**(16): 1937-48.
21. Hotez PJ, Zhan B, Bethony JM, Loukas A, Williamson A, Goud GN, et al. Progress in the development of a recombinant vaccine for human hookworm disease: the Human Hookworm Vaccine Initiative. Int J Parasitol. 2003; **33**(11): 1245-58.
22. Diemert DJ, Bethony JM, Hotez PJ. Hookworm vaccines. Clin Infect Dis. 2008; **46**(2): 282-8.
23. Bethony JM, Simon G, Diemert DJ, Parenti D, Desrosiers A, Schuck S, et al. Randomized, placebo-controlled, double-blind trial of the Na-ASP-2 hookworm vaccine in unexposed adults. Vaccine. 2008; **26**(19): 2408-17.
24. Ranjit N, Zhan B, Hamilton B, Stenzel D, Lowther J, Pearson M, et al. Proteolytic degradation of hemoglobin in the intestine of the human hookworm Necator americanus. J Infect Dis. 2009; **199**(6): 904-12.
25. Williamson AL, Brindley PJ, Abbenante G, Prociv P, Berry C, Girdwood K, et al. Cleavage of hemoglobin by hookworm cathepsin D aspartic proteases and its potential contribution to host specificity. FASEB J. 2002; **16**(11): 1458-60.
26. Williamson AL, Brindley PJ, Abbenante G, Datu BJ, Prociv P, Berry C, et al. Hookworm aspartic protease, Na-APR-2, cleaves human hemoglobin and serum proteins in a host-specific fashion. J Infect Dis. 2003; **187**(3): 484-94.
27. Williamson AL, Lecchi P, Turk BE, Choe Y, Hotez PJ, McKerrow JH, et al. A multi-enzyme cascade of hemoglobin proteolysis in the intestine of blood-feeding hookworms. J Biol Chem. 2004; **279**(34): 35950-7.
28. Loukas A, Bethony JM, Mendez S, Fujiwara RT, Goud GN, Ranjit N, et al. Vaccination with recombinant aspartic hemoglobinase reduces parasite load and blood loss after hookworm infection in dogs. PLoS Med. 2005; **2**(10): e295.
29. Pearson MS, Bethony JM, Pickering DA, de Oliveira LM, Jariwala A, Santiago H, et al. An enzymatically inactivated hemoglobinase from Necator americanus induces neutralizing antibodies against multiple hookworm species and protects dogs against heterologous hookworm infection. Faseb J. 2009; **23**(9): 3007-19.
30. Zhan B, Liu S, Perally S, Xue J, Fujiwara R, Brophy P, et al. Biochemical characterization and vaccine potential of a heme-binding glutathione transferase from the adult hookworm Ancylostoma caninum. Infect Immun. 2005; **73**(10): 6903-11.
31. Zhan B, Perally S, Brophy PM, Xue J, Goud G, Liu S, et al. Molecular Cloning, Biochemical Characterization and Partial Protective Immunity of the Heme-binding Glutathione S-Transferases from the Human Hookworm Necator americanus. Infect Immun.
32. Asojo OA, Homma K, Sedlacek M, Ngamelue M, Goud GN, Zhan B, et al. X-ray structures of Na-GST-1 and Na-GST-2 two glutathione S-transferase from the human hookworm Necator americanus. BMC Struct Biol. 2007; **7**: 42.
33. van Rossum AJ, Jefferies JR, Rijsewijk FA, LaCourse EJ, Teesdale-Spittle P, Barrett J, et al. Binding of hematin by a new class of glutathione transferase from the blood-feeding parasitic nematode Haemonchus contortus. Infect Immun. 2004; **72**(5): 2780-90.
34. Schuller DJ, Liu Q, Kriksunov IA, Campbell AM, Barrett J, Brophy PM, et al. Crystal structure of a new class of glutathione transferase from the model human hookworm nematode Heligmosomoides polygyrus. Proteins. 2005; **61**(4): 1024-31.

35. Zhan B, Perally S, Brophy PM, Xue J, Goud G, Liu S, et al. Molecular cloning, biochemical characterization, and partial protective immunity of the heme-binding glutathione S-transferases from the human hookworm Necator americanus. Infect Immun. 2010; **78**(4): 1552-63.
36. Xiao S, Zhan B, Xue J, Goud GN, Loukas A, Liu Y, et al. The evaluation of recombinant hookworm antigens as vaccines in hamsters (Mesocricetus auratus) challenged with human hookworm, Necator americanus. Exp Parasitol. 2008; **118**(1): 32-40.
37. Cody CL, Baraff LJ, Cherry JD, Marcy SM, Manclark CR. Nature and rates of adverse reactions associated with DTP and DT immunizations in infants and children. Pediatrics. 1981; **68**(5): 650-60.
38. Merck and Co. I. Recombivax HB® Product Insert. Whitehouse Station, NJ: Merck & Co., Inc.; 2003.
39. Ogutu BR, Apollo OJ, McKinney D, Okoth W, Siangla J, Dubovsky F, et al. Blood stage malaria vaccine eliciting high antigen-specific antibody concentrations confers no protection to young children in Western Kenya. PLoS One. 2009; **4**(3): e4708.
40. Thera MA, Doumbo OK, Coulibaly D, Laurens MB, Kone AK, Guindo AB, et al. Safety and immunogenicity of an AMA1 malaria vaccine in Malian children: results of a phase 1 randomized controlled trial. PLoS One. 2010; **5**(2): e9041.
41. Sacarlal J, Aide P, Aponte JJ, Renom M, Leach A, Mandomando I, et al. Long-term safety and efficacy of the RTS,S/AS02A malaria vaccine in Mozambican children. J Infect Dis. 2009; **200**(3): 329-36.
42. Velez ID, Gilchrist K, Martinez S, Ramirez-Pineda JR, Ashman JA, Alves FP, et al. Safety and immunogenicity of a defined vaccine for the prevention of cutaneous leishmaniasis. Vaccine. 2009; **28**(2): 329-37.
43. Vandepapeliere P, Horsmans Y, Moris P, Van Mechelen M, Janssens M, Koutsoukos M, et al. Vaccine adjuvant systems containing monophosphoryl lipid A and QS21 induce strong and persistent humoral and T cell responses against hepatitis B surface antigen in healthy adult volunteers. Vaccine. 2008; **26**(10): 1375-86.
44. Paavonen J, Naud P, Salmeron J, Wheeler CM, Chow SN, Apter D, et al. Efficacy of human papillomavirus (HPV)-16/18 AS04-adjuvanted vaccine against cervical infection and precancer caused by oncogenic HPV types (PATRICIA): final analysis of a double-blind, randomised study in young women. Lancet. 2009; **374**(9686): 301-14.
45. Konno R, Dobbelaere KO, Godeaux OO, Tamura S, Yoshikawa H. Immunogenicity, reactogenicity, and safety of human papillomavirus 16/18 AS04-adjuvanted vaccine in Japanese women: interim analysis of a phase II, double-blind, randomized controlled trial at month 7. Int J Gynecol Cancer. 2009; **19**(5): 905-11.
46. Toledo AC, Jr., Greco DB, Felga M, Barreira D, Gadelha Mde F, Speranza FA. Seroprevalence of hepatitis B and C in Brazilian army conscripts in 2002: a cross-sectional study. Braz J Infect Dis. 2005; **9**(5): 374-83.

Appendix A – Sample Informed Consent Comprehension Questionnaire

INFORMED CONSENT COMPREHENSION QUESTIONNAIRE

Phase 1 Study of the Safety and Immunogenicity of *Na*-GST-1/Alhydrogel® with or without GLA-AF in Brazilian Adults

____________________ ________________________ ________________________

ID# Last Name First Name

1. During the study, you'll receive a small part of the hookworm parasite, but it won't cause the illness in you……………………………………….…...……....T F
2. There is a chance you could get sick from this vaccine……………..……………..T F
3. Women enrolled in this study should not become pregnant up until 1 month after the last shot….………………………………………………….….……...T F
4. If you change your mind about being in the study after you are vaccinated, you can withdraw your consent……..……………………………….…………..…T F
5. This vaccine has been given to hundreds of people already, so we know it is completely safe…………………………………………..…….………..…T F
6. You'll have your blood drawn as part of this study……………………….….…T F
7. You'll get 3 shots in this study……………………………………………….…..…T F
8. If you feel sick during the study, you shouldn't tell anyone…………………...….T F
9. If you join the study, you need to be followed by the study team for 16 months.…T F
10. Everybody in this study will get the same kind of vaccine………………….….…T F

- Total number correct before review……………..……..……… ____/10
- Total number correct after review…………………………….. ____/10

Reviewed by ______________________________ Date ___/___/___ *d m y*

Volunteer signature _________________________ Date ___/___/___ *d m y*

Witness signature __________________________ Date ___/___/___ *d m y*

Appendix B – Schedule of Visits

Procedures	Blood Volume	Day	Pre[2]	0	1	3	7	14	28	56	57	59	63	70	84	112	113	115	119	126	140	200	290	380	470
Complete History/Physical			X																						
Obtain Informed Consent			X																						
Interim Clinical Evaluation				X	X	X	X	X	X	X	X	X	X	X	X	X	X	X	X	X	X	X	X	X	X
CBC[1]	2 mL		X	X				X		X				X		X				X					
PTT/PT INR	2 mL		X																						
ALT	5 mL		X	X				X		X				X		X				X					
Creatinine			X	X				X		X				X		X				X					
Glucose (random)			X																						
Urinalysis			X																						
Urine pregnancy test (females)			X	X						X						X									
HCV ELISA	10 mL		X																						
HBsAg ELISA			X																						
HIV ELISA			X																						
**VACCINATION**				**1**						**2**						**3**									
Anti-*Na*-GST-1 antibody ELISA	10 mL		X	X				X	X	X				X	X	X				X	X	X	X	X	
Total IgE				X				X	X	X				X	X	X				X	X	X	X	X	
Cellular assays	30 mL			X				X	X	X				X	X	X				X	X	X	X		
Stool examination			X																		X[3]			X[3]	
**Blood Volume (mL)**			30	50				50	40	50				50	40	50				50	40	40	40	10	
**Total Blood Volume (mL)**			30	80				130	170	220				270	310	360				410	450	490	530	540	540

[1]CBC parameters to be assessed for safety: WBC, absolute neutrophil count, hemoglobin, and platelet count
[2]To be completed within 90 days of enrolment.
[3]Part II of the study only.

Appendix C – Toxicity Table for Grading Laboratory Adverse Events

These tables are to be used to assess laboratory adverse events for those tests to be performed as part of the study.

ESTIMATING SEVERITY GRADE

GRADE 1 **Mild:** no effect on activities of daily living; no medical intervention/therapy required

GRADE 2 **Moderate:** partial limitation in activities of daily living (can complete $\geq$ 50% of baseline); no or minimal medical intervention/therapy required

GRADE 3 **Severe:** activities of daily living limited to < 50% of baseline; medical evaluation/therapy required

GRADE 4 **Potentially life threatening**

STUDY PART I – BELO HORIZONTE*				
**HEMATOLOGY**	**Grade 1**	**Grade 2**	**Grade 3**	**Grade 4**
Hemoglobin<br>*Males*<br>*Females*	 12.5 – 13.4 g/dL<br>11.0 – 11.9 g/dL	 10.5 – 12.4 g/dL<br>9.5 – 10.9 g/dL	 8.5 – 10.4 g/dL<br>8.0 – 9.4 g/dL	 <8.5 g/dL<br><8.0 g/dL
Hemoglobin – (*change from Day 0)*	0.6 – 1.5 g/dL	1.6 – 2.0 g/dL	2.1 – 5.0 g/dL	>5.0 g/dL
Platelets	125,000 – 140,000/mm^3	100,000 – 124,999/mm^3	25,000 - 99,999/mm^3	<25,000/mm^3
WBCs *(increase)*	10,800 – 15,000/mm^3	15,001 – 20,000/mm^3	20,001-25,000/mm^3	>25,000
WBCs *(decrease)*	2500 – 3500/mm^3	1500 – 2499/mm^3	1000 - 1499/mm^3	<1000/mm^3
ANC *(decrease)*	1000 – 1599/mm^3	750 – 999/mm^3	500 - 749/mm^3	<500/mm^3
**CHEMISTRIES**	**Grade 1**	**Grade 2**	**Grade 3**	**Grade 4**
Serum creatinine	1.5 – 1.7 mg/dL	1.8 – 2.0 mg/dL	2.1 – 2.5 mg/dL	>2.5 mg/dL or requires dialysis
ALT<br>*Males*<br>*Females*	 49 – 98 U/L<br>46 – 93 U/L	 99 – 195 U/L<br>94 – 185 U/L	 196 - 390 U/L<br>186 - 370 U/L	 >390 U/L<br>>370 U/L

*Based on the reference intervals of the Humberto Abrãao Laboratory.

STUDY PART II - AMERICANINHAS				
**HEMATOLOGY**	**Grade 1**	**Grade 2**	**Grade 3**	**Grade 4**
Hemoglobin<br>*Males*<br>*Females*	 11.0 – 12.0 g/dL<br>10.3 – 11.3 g/dL	 9.5 – 10.9 g/dL<br>8.5 – 10.2 g/dL	 8.0 - 9.4 g/dL<br>7.5 - 8.4 g/dL	 <8.0 g/dL<br><7.5 g/dL
Hemoglobin –<br>*(change from Day 0)*	0.6 – 1.0 g/dL	1.1 – 1.8 g/dL	1.9 – 4.0 g/dL	>4.0 g/dL
Platelets	110,000 – 130,000/mm^3	90,000 – 109,999/mm^3	25,000 - 89,999/mm^3	<25,000/mm^3
WBCs<br>*(increase)*	10,800 – 15,000/mm^3	15,001 – 20,000/mm^3	20,001- 25,000/mm^3	>25,000/mm^3
WBCs<br>*(decrease)*	2300 – 3200/mm^3	1400 – 2299/mm^3	900 - 1399/mm^3	< 900/mm^3
ANC<br>*(decrease)*	750 – 1000/mm^3	500 – 749/mm^3	300 - 499/mm^3	< 300/mm^3
**CHEMISTRIES**	**Grade 1**	**Grade 2**	**Grade 3**	**Grade 4**
Serum creatinine<br>*Males*<br>*Females*	 1.5 – 1.7 mg/dL<br>1.5 – 1.7 mg/dL	 1.8 – 2.0 mg/dL<br>1.8 – 2.0 mg/dL	 2.1 – 2.5 mg/dL<br>2.1 – 2.5 mg/dL	 >2.5 mg/dL or requires dialysis<br>>2.5 mg/dL or requires dialysis
ALT<br>*Males*<br>*Females*	 58 – 115 U/L<br>64 – 128 U/L	 116 – 230 U/L<br>129 – 255 U/L	 231 – 460 U/L<br>256 – 510 U/L	 >460 U/L<br>>510 U/L

Appendix D – Toxicity Table for Grading Unsolicited Systemic Adverse Events

These tables are to be used to grade unsolicited adverse events not described in **Table 4** of this protocol.

**Vital Signs***	**Mild (Grade 1)**	**Moderate (Grade 2)**	**Severe (Grade 3)**	**Potentially Life Threatening (Grade 4)**
Tachycardia – beats per minute	101-115	116-130	>130	ER visit or hospitalization for arrhythmia
Bradycardia – beats per minute	50-54	45-49	<45	ER visit or hospitalization for arrhythmia
Hypertension** (systolic, mm Hg)	141-150	151-155	>155	ER visit or hospitalization for hypertension
Hypertension** (diastolic, mm Hg)	91-95	96-100	>100	ER visit or hospitalization for hypertension
Hypotension** (systolic, mm Hg)	85-89 (and symptomatic)	80-84 (and symptomatic)	<80	ER visit or hospitalization for hypotensive shock
Respiratory Rate – breaths per minute	18-20	21-25	>25	Intubation
** Participant should be at rest for measurement of vital signs*<br>*** with repeat testing at same visit*				

**Systemic AE**	**Mild (Grade 1)**	**Moderate (Grade 2)**	**Severe (Grade 3)**	**Potentially Life Threatening (Grade 4)**
Anorexia	Loss of appetite without decreased oral intake lasting greater than 48 hours	Loss of appetite associated with decreased oral intake without significant weight loss	Loss of appetite associated with significant weight loss	ER visit or hospitalization
Diarrhea	2-3 loose stools/24 hours	4-5 loose stools/24 hours	>6 loose stools or requires outpatient IV hydration	ER visit or hospitalization
Constipation	Not Applicable	Persistent constipation requiring regular use of dietary modifications, laxatives, or enemas	Obstipation with manual evacuation indicated	ER visit or hospitalization
Fatigue	No interference w/activity	Some interference w/activity	Significant, prevents daily activity	ER visit or hospitalization
Arthritis	Mild pain with inflammation, erythema or joint swelling – but not interfering with function	Moderate pain with inflammation, erythema or joint swelling – interfering with function, but not with activities of daily living	Severe pain with inflammation, erythema or joint swelling –and interfering with activities of daily living	ER visit or hospitalization
Vasovagal episode (associated with a procedure of any kind)	Present without loss of consciousness	Present with transient loss of consciousness	Not Applicable	Not Applicable

**Systemic AE**	**Mild (Grade 1)**	**Moderate (Grade 2)**	**Severe (Grade 3)**	**Potentially Life Threatening (Grade 4)**
Vertigo	Causes no or minimal interference with usual daily activities	Causes greater than minimal interference with usual daily activities	Inability to perform daily activities	ER visit or hospitalization
Cough	Transient- no treatment	Persistent cough; treatment responsive	Paroxysmal cough; uncontrolled with treatment	ER visit or hospitalization
Bronchospasm, Acute	Transient; no treatment; 70% - 80% FEV_1 of peak flow	Requires treatment; normalizes with bronchodilator; FEV_1 50% - 70% (of peak flow)	No normalization with bronchodilator; FEV_1 25% - 50% of peak flow; or retractions present	ER visit or hospitalization
Dyspnea	Dyspnea on exertion	Dyspnea with normal activity	Dyspnea at rest	ER visit or hospitalization
Hypersensitivity	Transient flushing or rash	Rash; flushing; urticaria; dyspnea	Symptomatic bronchospasm, with or without urticaria; parenteral medications(s) indicated; allergy-related edema/angioedema; hypotension	ER visit or hospitalization
Illness or clinical AE NOT identified on the toxicity table	No interference with daily activities	Partial limitation in activities of daily living (can complete $\geq$50% of baseline); no or minimal medical intervention/therapy required	Activities of daily living limited to < 50% of baseline; medical evaluation/therapy required	ER visit or hospitalization
