# Supplementary material for: Safety and immunogenicity of the Na-GST-1 hookworm vaccine in Brazilian and American adults
Source: PLoS Negl Trop Dis. 2017 May 2;11(5):e0005574. doi: 10.1371/journal.pntd.0005574 (PMC5441635; doi:10.1371/journal.pntd.0005574)
Supplement: S3 Supporting Information — (PDF) [file pntd.0005574.s006.pdf]

PHASE 1 STUDY OF THE SAFETY AND IMMUNOGENICITY OF *NA*-GST-1/ALHYDROGEL® WITH DIFFERENT DOSES OF THE NOVEL IMMUNOSTIMULANT GLA-AF IN HEALTHY ADULTS

**Protocol Number:**	SVI-11-01
**Version (Date):**	10 (13 June 2013)
**US FDA IND Number:**	14616
**CNMC IRB Number:**	00001665
**GWU IRB Number:**	051109
**ClinicalTrials.gov ID:**	NCT01385189

Sponsor: **Albert B. Sabin Vaccine Institute**
2000 Pennsylvania Avenue, Suite 7100
Washington, DC 20006
Tel: 202-842-5025 Fax: 202-842-7689

This document is a confidential communication of the Sabin Vaccine Institute. Acceptance of this document constitutes agreement by the recipient that no unpublished information contained herein will be published or disclosed without Sabin Vaccine Institute's prior written approval, except that this document may be disclosed to appropriate Institutional Review Boards under the condition that they are required to keep it confidential.

<u>TEAM ROSTER</u>

Principal Investigator: **David Diemert, MD**
Sabin Vaccine Institute
2000 Pennsylvania Avenue
Washington, DC 20006
Tel: 202-994-2909
Email: david.diemert@sabin.org

Co-Investigators: **Stephen Teach, MD**
Roberta DeBiasi, MD
Grace Appiah
Kimberly Martin
Children's National Medical Center
Washington, DC

Shannon Grahek, PhD, MPH
Department of Microbiology, Immunology and Tropical Medicine
George Washington University
Washington, DC

Scientific Investigators: **Jeffrey Bethony, PhD**
Department of Microbiology, Immunology and Tropical Medicine
George Washington University
Washington, DC

Robert Hamilton, PhD
Dermatology, Allergy, and Clinical Immunology Reference Laboratory
Johns Hopkins University
Baltimore, MD

Regulatory Affairs: **Marva Loblack, MS, MBA**
Director of Regulatory and Quality Assurance
Sabin Vaccine Institute
Washington, DC, USA

Medical Monitor: **Kirsten Lyke, MD**
University of Maryland
Baltimore, MD

Participating Site

Clinical Trial Sites:

Clinical Research Center
Children's National Medical Center (CNMC)
111 Michigan Ave, NW
Washington, DC 20010
Tel: 202-476-5000

George Washington University
Dept. of Microbiology, Immunology & Tropical Medicine
2300 I St, NW
Ross 723-D
Washington, DC

Participating Laboratories

Clinical:

Division of Laboratory Medicine
Children's National Medical Center
111 Michigan Ave, NW
Washington, DC 20010

Immunology:

Clinical Immunology Laboratory (CIL)
Department of Microbiology, Immunology & Tropical Medicine
George Washington University Medical Center
Room 73X, Ross Hall
2300 Eye Street, NW
Washington, DC 200037

Johns Hopkins Dermatology, Allergy and Clinical Immunology (DACI) Reference Laboratory
Johns Hopkins University
Baltimore, Maryland

Table of Abbreviations

AE	Adverse Event
AESI	Adverse Event of Special Interest
CFR	Code of Federal Regulations
cGCP	Current Good Clinical Practices
cGMP	Current Good Manufacturing Practices
CIL	Clinical Immunology Laboratory (GWUMC)
CNMC	Children's National Medical Center
CRF	Case Report Form
DACI	Dermatology, Allergy, and Clinical Immunology Reference Laboratory
ELISA	Enzyme linked immunosorbent assay
FDA	Food and Drug Administration
GLA-AF	Gluco-Pyranosylphospho-Lipid A Aqueous Formulation
GST-1	Glutathione S-Transferase
GWUMC	George Washington University Medical Center
HBsAg	Hepatitis B surface antigen
HBV	Hepatitis B virus
hCG	Human choriogonadotropin
HCV	Hepatitis C virus
HHVI	Human Hookworm Vaccine Initiative
HIV	Human Immunodeficiency Virus
ICH	International Conference on Harmonization
IM	Intramuscular
INR	International Normalized Ratio
IRB	Institutional Review Board
MedDRA®	Medical Dictionary of Regulatory Activities
MITM	Microbiology, Immunology, and Tropical Medicine
Na	*Necator americanus*
PBMC	Peripheral Blood Mononuclear Cell
PT	Prothrombin time
PTT	Partial thromboplastin time
SAE	Serious Adverse Event
SMC	Safety Monitoring Committee
SVI	Sabin Vaccine Institute
US	United States
WBC	White blood cell

Table of Contents

TEAM ROSTER 2
Table of Abbreviations 4
Protocol Summary 9
1 Introduction 11
1.1 Background 11
1.2 Rationale for Developing a Hookworm Vaccine 12
1.5 *Na*-GST-1 Vaccines 19
1.6 Preclinical Toxicity Study of *Na*-GST-1 Hookworm Vaccines 19
1.7 Immunogenicity Studies with *Na*-GST-1 20
1.8 *In vitro* Tissue Cross-Reactivity Studies 21
1.9 Clinical Experience with *Na*-GST-1 22
1.10 Clinical Experience with Aluminum-Based Adjuvants 23
1.11 Clinical Experience with GLA-AF 24
1.12 Clinical Development Plan 24
2 Objectives 25
2.1 Primary Objective 25
2.2 Secondary Objectives 25
3 Study Site 25
4 Study Design 26
4.1 Overall Design 26
4.2 Sample Size and Estimated Duration of Study 28
4.3 Group Allocation 28
4.4 Blinding for Cohorts 1 and 2 28
5 Selection of Participants 29
5.1 Inclusion Criteria 29
5.2 Exclusion Criteria 29
6 Vaccine Preparation 30
6.1 Supplies 30
6.3 Preparation of Vaccine Doses 31
6.4 Vaccine Storage 31
6.5 Vaccine Accountability 31
6.6 Disposition of Used/Unused Supplies 32
7 Study Procedures 32
7.1 Individual Recruitment and Informed Consent 32
7.2 Inclusion 32
7.3 Enrollment 33
7.4 Immunization Procedure 33
7.5 Clinical Monitoring and Evaluation 33
7.6 Participant Symptom Diary 36
7.7 Laboratory Testing 36
7.8 Immunologic Testing 37
7.8.1 Anti-Na-GST-1 Antibody Assays 37
7.8.2 ImmunoCAP for IgG, IgG4 and IgE to Na-GST-1 38
7.8.3 Surface Plasmon Resonance (Biacore) 38
7.8.4 Detection and quantification of memory B cells to Na-GST-1 39
7.8.5 Detection of Cytokines and Chemokines 39
7.9 Storage of Participant Specimens 39
8 Adverse Events Monitoring and Reporting 39
8.1 Definitions 39

8.1.1 Adverse Event 39
8.1.2 Serious Adverse Event (SAE) 39
8.1.3 Adverse Events of Special Interest (AESIs) 40
8.2 Assessment of Adverse Events 40
8.2.1 Identification of AEs 40
8.2.2 Determination of Severity 41
8.2.3 Association with Receipt of the Study Vaccine 43
8.3 Adverse Event Reporting 43
8.4 Adverse Event Monitoring 44
8.4.1 Medical Monitor 44
8.4.2 Safety Monitoring Committee (SMC) 44
8.5 Criteria for Suspension of Further Injections 45
8.6 Treatments That Could Potentially Interfere with Vaccine-Induced Immunity 46
8.7 Criteria for Suspension of Injections 46
8.8 Criteria for Deferral of Injections 46
8.9 Criteria for Withdrawal of an Individual Participant from the Study 47
8.10 Breaking the Study Blind for Cohorts 1 and 2 47
9 Data Collection and Monitoring 48
9.1 Source Documentation and Case Report Forms 48
9.2 Study Documentation 48
9.2.1 Study Reports 48
9.3 Retention of Records 48
9.4 Protocol Revisions 48
9.5 Clinical Investigator's Brochure 49
9.6 Study Monitoring 49
10 Statistical Considerations 49
10.1 General Design 49
10.1.1 Description of the Statistical Methods to Be Employed 49
10.2 Sample Size 52
11 Protection of Human Subjects 52
11.1 Institutional Review Board Review 52
11.2 Informed Consent 53
11.3 Risks 53
11.3.1 Venipuncture 53
11.3.2 Immunization with Na-GST-1/Alhydrogel® 54
11.3.3 Immunization with Na-GST-1/Alhydrogel® plus GLA-AF 54
11.4 Precautions taken to Minimize Risks 54
11.5 Benefits 54
11.6 Confidentiality 54
11.7 Compensation 55
11.8 Publication of Study Results 55
12 References 55

Appendix A – Schedule of Visits 59
Appendix B – Toxicity Table for Grading Laboratory Adverse Events 60
Appendix C – Toxicity Table for Grading Unsolicited Systemic Adverse Events 61

List of Figures and Tables

Figure 1: Vaccination of Brazilian Adults with Previous Hookworm Infection with 10 µg *Na*-ASP-2/Alhydrogel® induced immediate-type urticarial reactions 13
Figure 2: Anti-*Na*-ASP-2 IgE antibody levels in residents of a hookworm-endemic region of northeastern Minas Gerais state, Brazil 14
Figure 3: Comparison of GST dimers from parasitic nematodes, *N. americanus* (*Na*) and *Heligomosoides polygyrus* (*Hpol*) of the Nu class and comparison with a GST of the Sigma class. 15
Figure 4: Geometric mean titers of the IgG1 and IgG2 antibody responses of vaccinated dogs against r*Ac*-GST-1 formulated with GlaxoSmithKline's AS03 adjuvant 16
Figure 5: Anti-*Na*-GST-1 IgE levels in (A) adults and children and (B) young children aged 1-10 years (n=128) living in a hookworm-endemic area of Brazil. 17
Figure 6: Anti-*Na*-GST-1 IgE levels in a subset of adults and children (n=179) living in a hookworm-endemic area of Brazil.. 18
Figure 7: Anti-*Na*-GST-1 IgG levels (arbitrary units) in Sprague-Dawley Rats vaccinated with recombinant *Na*-GST-1. 20
Figure 8: Geometric mean anti-*Na*-GST-1 IgG antibody units 2 weeks after the 2nd Immunization of BALB/c mice with *Na*-GST-1/Alhydrogel® with or without co-administration of CPG 10104 and CPG 10105. 21
Figure 9: Geometric mean anti-*Na*-GST-1 IgG antibody units 2 weeks after the 3rd Immunization of Healthy Hookworm-Unexposed Brazilian Adults with *Na*-GST-1/Alhydrogel® with (A) or without (B) co-administration of 2.5 µg GLA-AF (SVI-10-01). 23

Table 1: Study Design 27
Table 2: Vaccination Schedule 27
Table 3: Assessment of Solicited Adverse Event Severity 42

PRINCIPAL INVESTIGATOR'S STATEMENT:

I, the undersigned, have reviewed this protocol, including Appendices, and will conduct the clinical study as described and will adhere to the principles of the ICH/cGCP as well as all applicable regulatory requirements. I have read and understood the contents of the Investigator's Brochure provided by the Human Hookworm Vaccine Initiative (HHVI) of the Albert B. Sabin Vaccine Institute.

David Diemert, MD

____
Signature

____
Date

Protocol Summary

**Title**	**Phase 1 Study of the Safety and Immunogenicity of *Na*-GST-1/Alhydrogel® with Different Doses of the Novel Immunostimulant GLA-AF in Healthy Adults**
**Study Population**	Healthy male and non-pregnant female volunteers aged 18-45 years, inclusive.
**Rationale**	*Na*-GST-1 is a protein expressed during the adult stage of the *Necator americanus* hookworm life cycle that is thought to play a role in the parasite's degradation of host hemoglobin for use as an energy source. Vaccination with recombinant GST-1 has protected dogs and hamsters from infection in challenge studies. This study will evaluate the safety and immunogenicity of two formulations of *Na*-GST-1 in healthy adult volunteers when co-administered with different concentrations of the immunostimulant GLA-AF.
**Study Design**	Double-blind, randomized, dose-escalation phase 1 clinical trial in healthy, hookworm-naïve adults:

- Study site: Clinical Research Center, Children's National Medical Center, Washington, DC
- Number of participants: **40 in 5 groups**. Two different doses of GLA-AF will be tested: 1 µg and 5 µg. Cohorts will be enrolled consecutively, with the first receiving 10 µg *Na*-GST-1, the second and third receiving 30 µg *Na*-GST-1, and the fourth and fifth receiving100 µg *Na*-GST-1.

- Immunization schedule: Study days 0, 56 and 112.
- Route: IM in the deltoid muscle.
- Doses of *Na*-GST-1 to be tested: 10, 30 and 100 µg.
- Doses of Alhydrogel®: 80, 240 and 800 µg for the 10, 30 and 100 µg doses of *Na*-GST-1, respectively.
- Doses of GLA-AF to be tested: 1 µg and 5 µg.

- Study duration: 24 months; each participant will be followed for a total of 16 months.

Immunization Schedule

Cohort	Total Participants	Immunization Schedule		
		Day 0	Day 56	Day 112
1	12	A (n=4) + D (n=8)	A (n=4) + D (n=8)	A (n=4) + D (n=8)
2	12	B (n=4) + E (n=8)	B (n=4) + E (n=8)	B (n=4) + E (n=8)
3	4	F (n=4)	F (n=4)	F (n=4)
4	4	C (n=4)	C (n=4)	C (n=4)
5	8	G (n=8)	G (n=8)	G (n=8)
Total	40	A: 10 µg *Na*-GST-1/Alhydrogel<br>B: 30 µg *Na*-GST-1/Alhydrogel<br>C: 100 µg *Na*-GST-1/Alhydrogel<br>D: 10 µg *Na*-GST-1/Alhydrogel/GLA-AF (1 µg)<br>E: 30 µg *Na*-GST-1/Alhydrogel/GLA-AF (1 µg)<br><br>F: 30 µg *Na*-GST-1/Alhydrogel/GLA-AF (5 µg)<br>G: 100 µg *Na*-GST-1/Alhydrogel/GLA-AF (5 µg)		

Objectives

Primary

1. To estimate the frequency of vaccine-related adverse events, graded by severity, for each dose and formulation of *Na*-GST-1.

Secondary

1. To determine the dose and formulation that generates the highest anti-*Na*-GST-1 antibody response following vaccination.
2. To determine the dose, formulation, and number of injections of *Na*-GST-1 that generates the antibody response of greatest affinity.
3. To determine the dose and formulation of the *Na*-GST-1 vaccine that results in the most robust production of *Na*-GST-1 specific B cells and subtypes (memory or plasma).
4. To perform exploratory studies of the cellular immune responses to the *Na*-GST-1 antigen both before and after immunization.

Product Descriptions

The *Na*-GST-1 formulations to be studied contain the recombinant *Na*-GST-1 protein expressed by *Pichia pastoris*. Purified *Na*-GST-1 was subsequently adsorbed onto aluminum hydroxide gel (Alhydrogel®) and suspended in a solution containing 10% glucose and 10 mM imidazole. The final concentration of *Na*-GST-1 in the drug product is 0.1 mg/ml whereas that of Alhydrogel® is 0.8 mg/ml. Different doses of *Na*-GST-1 will be delivered by injecting different volumes of the 0.1 mg/ml *Na*-GST-1 preparation.

The *Na*-GST-1/Alhydrogel®/GLA-AF formulation will be prepared immediately prior to vaccination by adding an appropriate volume of GLA-AF (Gluco-Pyranosylphospho-Lipid A Aqueous Formulation) to *Na*-GST-1/Alhydrogel® (or vice versa) and withdrawing an appropriate volume to administer the desired amount of *Na*-GST-1.

1 INTRODUCTION

1.1 Background

There is an urgent need for new tools to control human hookworm infection and to reduce its burden of disease in developing countries. This is especially true for children and women of reproductive age who represent populations that are highly vulnerable to the effects of hookworm disease. Up to 65,000 deaths annually have been attributed to human hookworm infection.[1] However, the mortality figures pale in comparison to global disease burden estimates that suggest that hookworm may account for the loss of up to 22 million Disability-Adjusted Life Years annually.[2] With the exception of malaria, hookworm is the most important parasitic disease of humans.

Human hookworm infection is a soil-transmitted helminth infection caused by the nematode parasites *Necator americanus* and *Ancylostoma duodenale*. It is one of the most common chronic infections of humans, afflicting up to 740 million people in the developing nations of the tropics.[2] The largest number of cases occurs in impoverished rural areas of sub-Saharan Africa, Southeast Asia, China, and the tropical regions of the Americas. Approximately 3.2 billion people are at risk for hookworm infection in these areas. *N. americanus* is the most common hookworm worldwide, whereas *A. duodenale* is more geographically restricted.[3]

Hookworm transmission occurs when skin comes into contact with infective third-stage larvae (L3) in fecally-contaminated soil. The L3 have the ability to penetrate the skin, usually of the hands, feet, arms, buttocks and legs. The L3 invade human tissues and enter the gastrointestinal tract where they molt to the adult stage approximately 5-9 weeks following initial host entry. Adult hookworms are approximately 1 cm long parasites that cause host injury by attaching to the mucosa and submucosa of the small intestine to produce intestinal blood loss. There is a direct relationship between hookworm intensity (as determined by fecal egg counts) and host blood loss; typically the presence of between 40 and 160 adult hookworms in the intestine results in blood loss sufficient to cause anemia and malnutrition. The term "hookworm disease" refers primarily to the iron deficiency anemia and protein losses that occur in moderate and heavy infections.[4] When host iron stores become depleted, there is a direct correlation between hookworm intensity and reduced host hemoglobin, serum ferritin, and protoporphyrin. Because of their low iron stores, children and women of reproductive age are the populations considered the most vulnerable to hookworm-associated blood loss.[4-11]

In children, chronic hookworm infection and the resultant iron deficiency anemia have been shown to impair physical and intellectual development.[3, 12, 13] Preschool children are particularly vulnerable to the effects of hookworm anemia and disease.[8] In addition to its health impact on children, hookworm infection also affects adults. Unlike other soil-transmitted helminth infections, such as ascariasis and trichuriasis, in which the highest intensity infections occur almost exclusively in school-aged children, it has been shown that high-intensity hookworm infections may also occur in adult populations.[14-16]

The primary approach to hookworm control worldwide has been the frequent and periodic mass administration of benzimidazole anthelminthics to school-aged children living in high-prevalence areas. In 2001, the World Health Assembly adopted Resolution 54.19 which urges

member states to provide regular anthelminthic treatment to high-risk groups with the target of regular treatment of at least 75% of all at-risk school-aged children. However, reported cure rates for a single dose of a benzimidazole vary from as low as 19% (single dose) and 45% (repeated dose) for mebendazole to 61% (400 mg) and 67% (800 mg) for albendazole.[17-20] These concerns have prompted interest in developing alternative tools for hookworm control.[3, 21, 22] Vaccination to prevent the anemia associated with moderate and heavy intensity hookworm infection would alleviate the public health deficiencies of drug treatment alone.

1.2 Rationale for Developing a Hookworm Vaccine

The feasibility of developing a hookworm vaccine is based on the previous success of using live, irradiated hookworm larvae (L3 stage) as a vaccine for canine hookworm infection. This provided the experimental basis for the commercial development of a canine hookworm vaccine, which was marketed in the United States during the early 1970s. However, it is not realistic to develop a live L3 vaccine for humans due to multiple reasons including high production costs, challenging storage requirements (8-10°C), a short shelf-life, and a lack of sterilizing immunity.

Alternatively, the strategy being pursued is to identify key hookworm proteins to which protective immune responses are directed in the animal models for this infection (namely the canine model) and to produce these as recombinant proteins that could then be used as vaccine antigens. This effort has focused primarily on identifying antigens expressed by the invading larvae (L3). In addition, a separate strategy has been to identify targets of the adult stage of the hookworm lifecycle; since hookworms attach onto the intestinal lumen and ingest host blood, antibodies could also be ingested that if directed against key hookworm proteins, would interfere with their function, ultimately resulting in the death or reduced fecundity of the worm.

As originally conceived, an eventual hookworm vaccine would ideally consist of a combination of one or more proteins targeting the larval stage of the hookworm life cycle together with one or more antigens targeting the adult stage. Such a vaccine could therefore potentially interfere with both distinct stages of the hookworm life cycle.

1.3 Prior Clinical Experience with Hookworm Vaccines

The first hookworm vaccine to be tested in humans was the *Na*-ASP-2 (Ancylostoma Secreted Protein-2 of *N. americanus*) Hookworm Vaccine, consisting of recombinant *Na*-ASP-2 expressed in *Pichia pastoris* and adsorbed to aluminum hydroxide gel (Alhydrogel®). *Na*-ASP-2 is an excretory/secretory product produced by infective *N. americanus* larvae upon penetration of human skin. In animal models, vaccination with this recombinant antigen was shown to result in reduced worm burdens after challenge infection. Accordingly, a phase 1 clinical trial of several different dose concentrations of the vaccine was conducted in healthy, hookworm-naïve adults living in the United States which showed the formulation to be safe, well-tolerated and immunogenic.[23]

However, upon testing the vaccine in adults who had previously been infected with hookworm in an endemic area of Brazil (protocol #SVI-06-02), several volunteers in the first (i.e., lowest dose) cohort to be vaccinated developed generalized urticaria within 1-2 hours of immunization. Due to these immediate-type hypersensitivity reactions, vaccinations in this study were halted and

have not been resumed. Subsequent investigations revealed that the volunteers who developed urticaria upon their first dose of *Na*-ASP-2 had elevated levels of baseline (i.e., pre-vaccination) IgE to the vaccine antigen (**Figure 1**). In order to investigate the prevalence and age distribution of IgE to *Na*-ASP-2 in a population living in a typical hookworm-endemic area, a seroepidemiological survey was conducted in an endemic region of Brazil; this study revealed that even in young children, a significant proportion of individuals have detectable levels of IgE to this protein, likely due to previous infection with *N. americanus* (**Figure 2**). In addition, similar findings were demonstrated for other larval proteins that were being considered as vaccine candidates.

Figure 1: Vaccination of Brazilian Adults with Previous Hookworm Infection with 10 µg *Na*-ASP-2/Alhydrogel® induced immediate-type urticarial reactions. Anti-*Na*-ASP-2 IgE antibody levels were measured in sera collected immediately prior to the first vaccination by ImmunoCAP in individuals vaccinated with the Butang hepatitis B vaccine (n=2, black squares) or 10 µg *Na*-ASP-2/Alhydrogel. For those receiving *Na*-ASP-2, IgE antibody levels are shown for individuals who developed urticaria (n=3, red circles) and those who did not (n=4, grey circles).

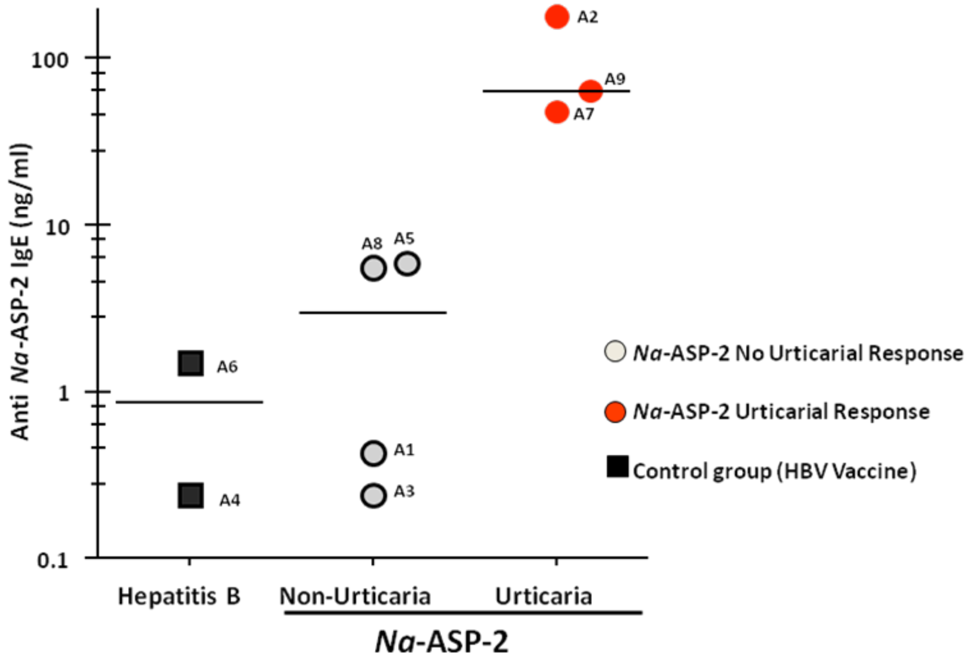

Figure 2: Anti-*Na*-ASP-2 IgE antibody levels in residents of a hookworm-endemic region of northeastern Minas Gerais state, Brazil. Antibody levels were measured by ELISA; each dot represents an individual. Levels are shown for individuals of various age groups, in addition to the 9 participants of the Phase 1 trial of *Na*-ASP-2 in Brazil (protocol #SVI-06-02) and the participants of the first Phase 1 trial of *Na*-ASP-2 that was conducted in hookworm-naïve adults in the United States (protocol #SVI-04-01).23

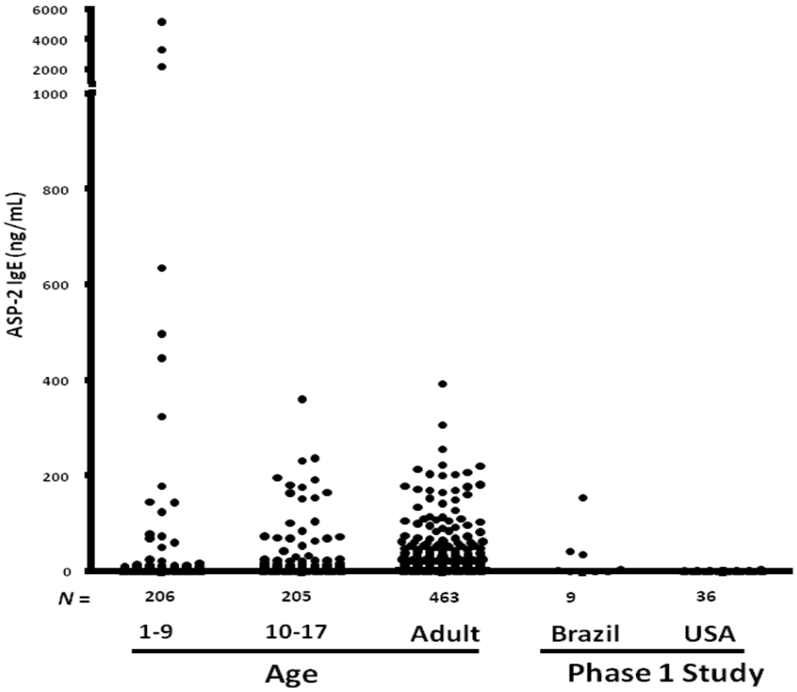

<u>Due to these cumulative data, clinical development of the *Na*-ASP-2 and other larval-stage antigens as candidate vaccines has been halted</u>. Instead, the current strategy is to develop antigens expressed during the <u>adult</u> stage of the hookworm life cycle that play a role in digesting the host hemoglobin that is used by the worm as an energy source. These antigens are relatively hidden from the human immune system during natural infection, and hence have a lower likelihood of inducing antigen-specific IgE in exposed/infected individuals. This should make the potential for inducing allergic reactions upon vaccination much less likely.

1.4 Rationale for Developing the *Na*-GST-1 Hookworm Vaccine

The nutritional and metabolic requirements of the adult hookworm living in the human intestine are dependent upon degradation of host hemoglobin that has been ingested by the worm. *N. americanus* hookworms depend on host hemoglobin for survival;[24] following hemolysis, adult hookworms use an ordered cascade of hemoglobinases to cleave hemoglobin into smaller molecules.[24-29] Following hemoglobin digestion, the freed heme generates toxic oxygen radicals that can be bound and detoxified by molecules such as glutathione S-transferase-1 (GST-1).[30-32] GST-1 of *N. americanus* (*Na*-GST-1) is a critical enzyme that plays a role in parasite blood

feeding; when used as a vaccine, we hypothesize that the antigen will induce anti-enzyme neutralizing antibodies that will interfere with parasite blood-feeding and cause parasite death or reduce worm fecundity.

Na-GST-1 is a 24 kDa protein with peroxidase enzymatic activity that catalyzes the conjugation of reduced glutathione to a variety of electrophiles.[30-32] This hookworm protein belongs to a recently named Nu class of nematode GSTs, which also includes GSTs from the blood-feeding parasite of ruminants, *Haemonchus contortus*, and the rodent nematode *Heligomosoides polygyrus*. This new class is characterized by diminished peroxidase activity relative to other classes of GSTs, but elevated binding capacity for heme and related products.[30, 32-35] X-ray crystallography of *Na*-GST-1 demonstrates that the protein can form homodimers in solution, which create atypically large binding cavities accessible to a diversity of ligands, including heme (**Figure 3**).[32] *Na*-GST-1 binds heme at high affinity *in vitro*.[30, 35] Because both heme and hematin contain oxidative iron, these molecules are potent generators of toxic reactive oxygen species that could potentially damage parasite macromolecules. *In vivo*, hookworm GSTs may therefore bind and detoxify the heme and hematin byproducts generated during the blood degradation process.

Figure 3: Comparison of GST dimers from parasitic nematodes, *N. americanus* (*Na*) and *Heligomosoides polygyrus* (*Hpol*) of the Nu class and comparison with a GST of the Sigma class. A) Superposition of GST dimers reveals that they are very similar, however, Nu class (*Na*-GST-1, magenta; *Na*-GST-2, gold; *Hpol*GST, green) have a more accessible binding cavity than Sigma class (*Hs*GST, cyan). The path to the binding cavity is indicated by the red arrow. The surface plots of Nu class GSTs b) *Hpol*GST, c) *Na*-GST-1, d) *Na*-GST-2 reveal larger access way to binding cavity than e) Sigma class GST (*Hs*GST).32

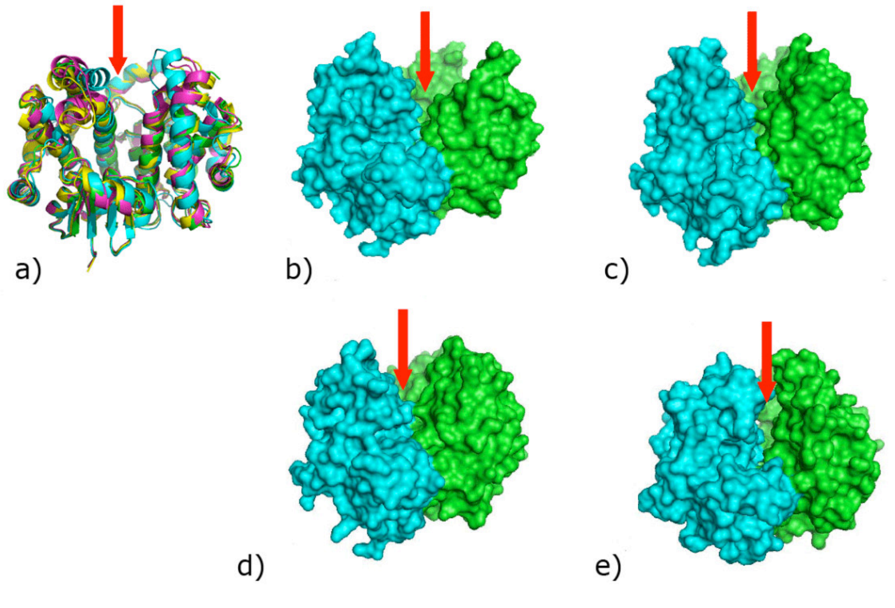

Based on their putative role in hookworm blood feeding, both *Na*-GST-1 and its orthologue from the canine hookworm *Ancylostoma caninum* (*Ac*-GST-1) were tested as experimental vaccines. In dogs, vaccination with yeast-expressed recombinant *Ac*-GST-1 resulted in high levels of antigen-specific antibody (**Figure 4**); following challenge with *A. caninum* infective larvae, significantly lower host worm burdens and fecal egg counts were observed compared to control animals vaccinated only with adjuvant.[30] In hamsters, vaccination with recombinant *Ac*-GST-1 also resulted in substantially lower worm burdens (51-54%) following heterologous challenge with *N. americanus* infective larvae compared to controls, as did vaccination with recombinant *Na*-GST-1 followed by homologous larval challenge.[30, 31, 36] Because of these encouraging preclinical results, *Na*-GST-1 was manufactured according to current good manufacturing practices (cGMP) and formulated on Alhydrogel® in preparation for clinical trials.

Figure 4: Geometric mean titers of the IgG1 and IgG2 antibody responses of vaccinated dogs against r*Ac*-GST-1 formulated with GlaxoSmithKline's AS03 adjuvant. Vaccination time points (1, 2, 3, and 4) and challenge day (C) are marked with arrows.[30]

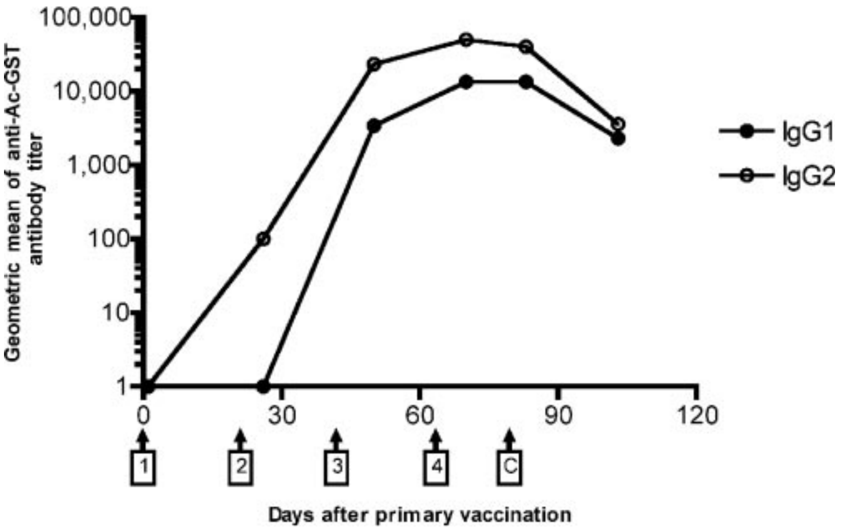

Most importantly, extensive studies have been conducted to test for sensitization to the *Na*-GST-1 protein in individuals living in a hookworm endemic area who have been repeatedly exposed and infected with *N. americanus*. As shown in **Figure 5**, over 1000 individuals of all ages from a hookworm endemic area of Brazil have been tested for serum IgE antibodies to *Na*-GST-1 using an indirect ELISA. In addition, a subset of these serum samples stratified by age and infection status (n = 179) underwent confirmatory testing at the Johns Hopkins Dermatology, Allergy and Clinical Immunology (DACI) Reference Laboratory (Baltimore, Maryland) using a custom ImmunoCAP assay. The ImmunoCAP method is considered the standard for measuring specific IgE to antigens in serum. This confirmatory testing demonstrated that none of the samples had *Na*-GST-1 IgE values above the clinical cut-off of 0.35 kU_A/L (**Figure 6**).

An advisory meeting was held by the HHVI on March 19, 2010, at George Washington University with several external experts to review the cumulative immunoepidemiology data on *Na*-GST-1. Based on the results of the testing of a population living in a highly endemic area for IgE to *Na*-GST-1 (by both an indirect ELISA and the ImmunoCAP method), the opinion of the advisory group was that the likelihood of inducing immediate-type hypersensitivity reactions by vaccinating individuals living in hookworm-endemic areas with *Na*-GST-1 is low and likely not more than that associated with any new vaccine antigen entering clinical trials. The situation with *Na*-GST-1 is therefore very different from that of *Na*-ASP-2 in that repeated infection with hookworm does not seem to induce an IgE response to the antigen, most likely due to the fact that it is a protein found in the digestive tract of adult hookworms and is therefore relatively hidden from the human immune system. This lack of antigen-specific IgE in people living in an area of high transmission has served as a major justification for advancing development of *Na*-GST-1 as a candidate vaccine antigen.

Figure 5: Anti-*Na*-GST-1 IgE levels in (A) adults and children and (B) young children aged 1-10 years (n=128) living in a hookworm-endemic area of Brazil.

IgE levels (optical density at 492 nm) were measured by ELISA.

A.

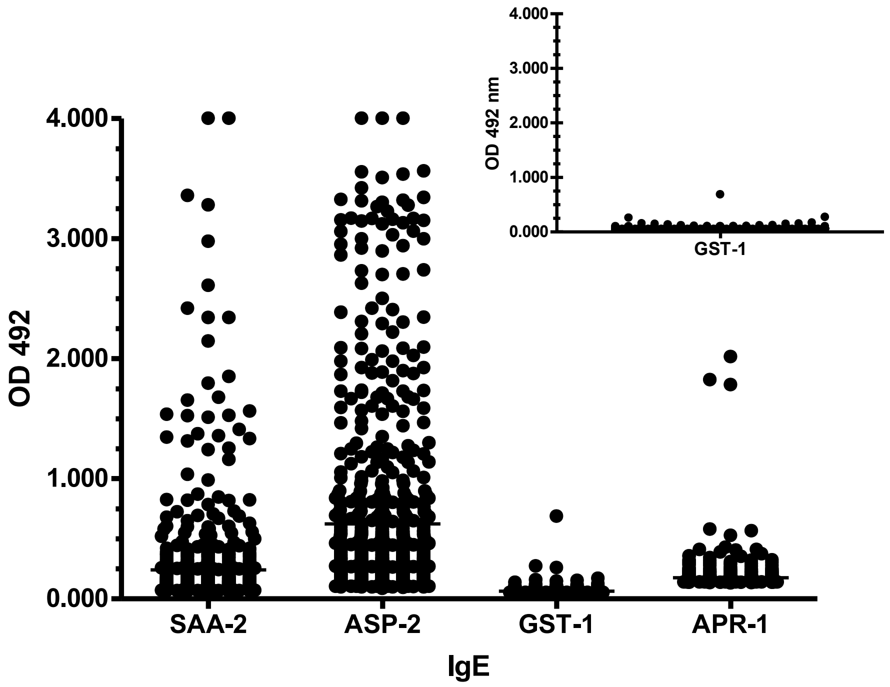

B.

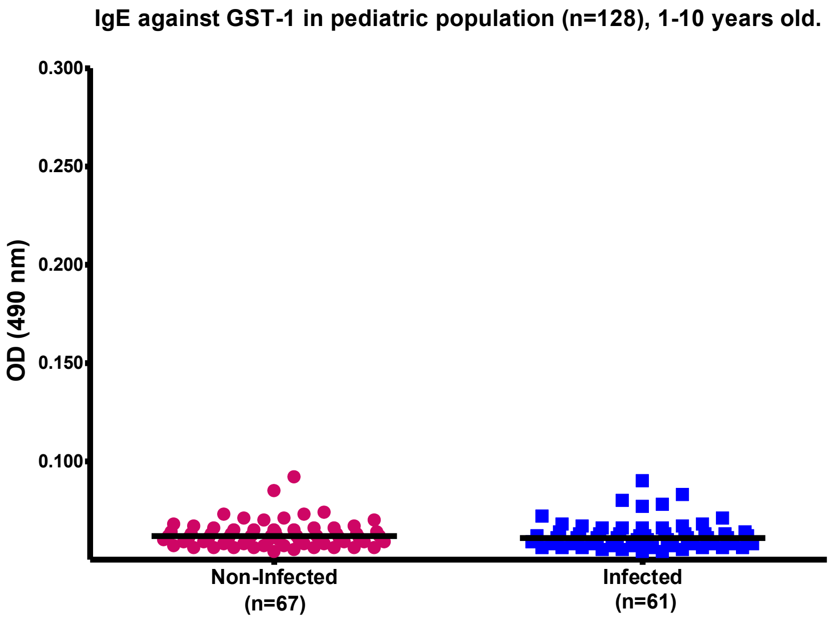

Figure 6: Anti-*Na*-GST-1 IgE levels in a subset of adults and children (n=179) living in a hookworm-endemic area of Brazil. IgE levels (kU_A/L) were measured by custom ImmunoCAP.

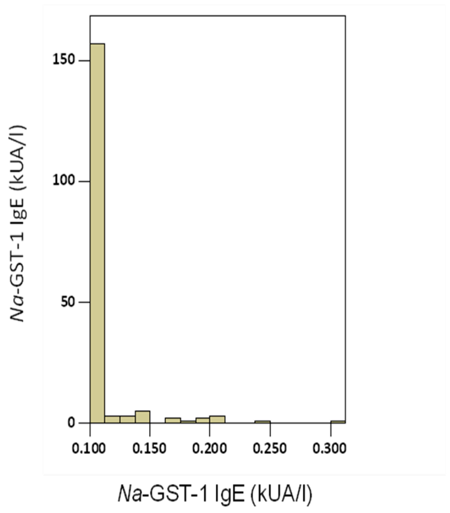

Na-GST-1 has been successfully manufactured and tested in the laboratory and in animals with both Alhydrogel® and Alhydrogel® plus GLA-AF. *Na*-GST-1 has been shown to be pure, potent, and stable in both of these two formulations. *Na*-GST-1 is being tested in humans for the first

time in a phase 1 study of *Na*-GST-1/Alhydrogel® with or without GLA-AF at a single dose concentration of 2.5 µg in healthy Brazilian adults.

1.5 *Na*-GST-1 Vaccines

The *Na*-GST-1 vaccine formulations to be tested in this study consist of the 24-kDa recombinant protein *Na*-GST-1, adsorbed to an adjuvant, Alhydrogel® (aluminum hydroxide suspension) either with or without the addition of two different dose concentrations (1 µg or 5 µg) of Gluco-Pyranosylphospho-Lipid A Aqueous Formulation (GLA-AF). For the *Na*-GST-1/Alhydrogel®/GLA-AF formulations, the GLA-AF will be added to the Alhydrogel® formulation immediately prior to immunization. The active ingredient in both vaccine formulations is the recombinant *Na*-GST-1 protein that is derived by fermentation of *Pichia pastoris* yeast cells genetically engineered to express *Na*-GST-1.

1.6 Preclinical Toxicity Study of *Na*-GST-1 Hookworm Vaccines

A 9-week toxicity study of GMP-grade *Na*-GST-1 (Aeras Lot# 09-69F-001) in rats was conducted at Frontier Biosciences, Inc./WestChina-Frontier Pharmatech, Co., China, according to current Good Laboratory Practices. The purpose of the study was to assess potential toxicity of vaccination by multiple IM injections of *Na*-GST-1 adjuvanted with Alhydrogel®, Alhydrogel® plus CPG 10104 and Alhydrogel® plus GLA-AF in Sprague-Dawley Rats. The toxicity study was conducted on an accelerated and extended dosing schedule of one injection on Days 0, 21, 42, and 63. In this rat study, 7 groups of animals (15 males and 15 females per group) were vaccinated by IM injection with 13.5 or 27 µg *Na*-GST-1 in diluent (10% glucose, 10 mM imidazole) or with the same dose levels of *Na*-GST-1 formulated with Alhydrogel®, Alhydrogel® plus CPG 10104 (100 or 300 µg) and Alhydrogel® plus GLA (2 or 20 µg). Four control groups, each consisting of 15 rats per sex, were also vaccinated with either the diluent vehicle, Alhydrogel® alone, Alhydrogel® plus CPG 10104, or Alhydrogel® plus GLA-AF.

Intramuscular injection with *Na*-GST-1/Alhydrogel® or *Na*-GST-1/Alhydrogel®/GLA-AF did not have any adverse effects on survival, body weights, or organ weights or produce any clinical signs of toxicity or abnormal gross necropsy findings in the toxicology study in Sprague-Dawley Rats. Thus, this study did not indicate any safety issues with *Na*-GST-1 adjuvanted with Alhydrogel® with or without the addition of GLA-AF.

There were no test article-related mortalities, clinical signs or effects on body weights or food consumption. Hematology, clinical chemistry, gross pathology and organ weights were unaffected at any dose. Histopathological evaluation of tissues revealed no unexpected effects associated with treatment with *Na*-GST-1 adsorbed to Alhydrogel® or *Na*-GST-1/Alhydrogel® co-administered with GLA-AF. Mild local granulomatous inflammation was observed in the muscle and occasionally in the skin of the injection site in groups treated with formulations containing Alhydrogel® with or without *Na*-GST-1 or the combination of *Na*-GST-1/Alhydrogel® plus GLA-AF. This finding was likely related to the Alhydrogel®, which is well-known to cause granulomatous inflammation.

1.7 Immunogenicity Studies with *Na*-GST-1

Several preclinical animal studies have been conducted in both mice and rats to assess the immunogenicity of *Na*-GST-1 in combination with different adjuvants. First, a study conducted in Sprague-Dawley Rats (HR-25-00) demonstrated that the addition of an adjuvant to recombinant *Na*-GST-1 was necessary, since administration of the recombinant protein without an adjuvant resulted in minimal specific antibody responses (**Figure 7**).

Figure 7: Anti-*Na*-GST-1 IgG levels (arbitrary units) in Sprague-Dawley Rats vaccinated with recombinant *Na*-GST-1. Rats were vaccinated twice on days 0 and 28 with 60 µg *Na*-GST-1 with or without Alhydrogel (480 µg), Alhydrogel (480 µg) + CPG 10104 (50 µg), or Alhydrogel (480 µg) + CPG 10105 (50 µg). Antibody measurements were performed on blood collected 2 weeks after the final vaccination.

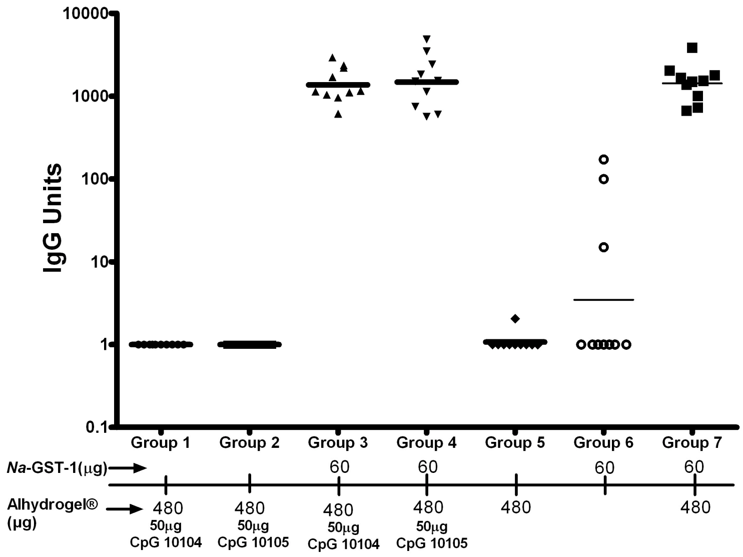

A second study was conducted in BALB/c mice to assess the effect of co-administering CPG 10104 with recombinant *Na*-GST-1/Alhydrogel® (**Figure 8**). In this study, mice were vaccinated with *Na*-GST-1/Alhydrogel® at antigen doses ranging from of 0.01 to 31.25 µg *Na*-GST-1 with or without CPG 10104 (5 µg) or CPG 10105 (5 µg). CPG 10105 is a CpG oligodeoxynucleotide sequence that is similar to CPG 10104 but that is not being proposed to be tested in the study described in this protocol. Mice were vaccinated twice intramuscularly at a 3-week interval, with blood collected for anti-*Na*-GST-1 IgG ELISA two weeks after the second immunization. This study demonstrated a large, highly significant increase in IgG specific for *Na*-GST-1 in the group administered *Na*-GST-1/Alhydrogel®/CPG 10104 compared to that administered only *Na*-GST-1/Alhydrogel® as shown in **Figure 8**.

Figure 8: Geometric mean anti-*Na*-GST-1 IgG antibody units 2 weeks after the 2nd Immunization of BALB/c mice with *Na*-GST-1/Alhydrogel® with or without co-administration of CPG 10104 and CPG 10105.

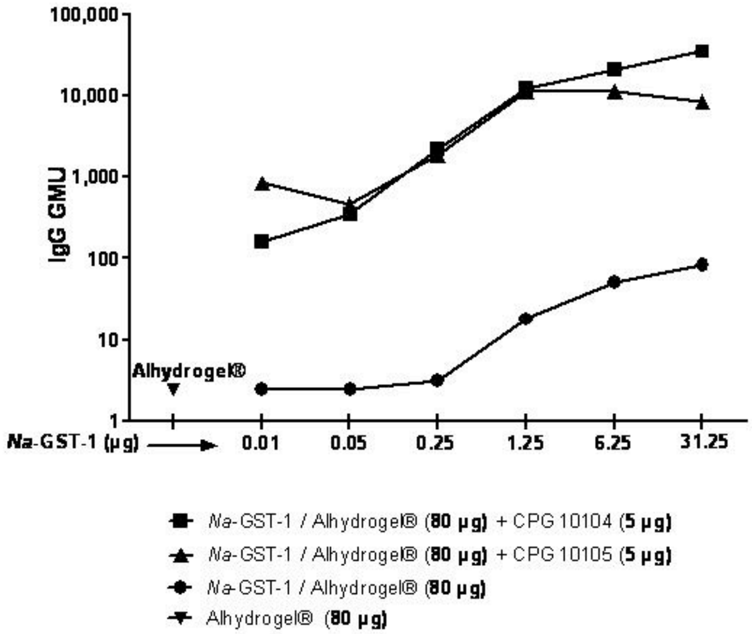

1.8 *In vitro* Tissue Cross-Reactivity Studies

GST-1 is an important detoxification enzyme that exists in different species of organisms. *Na*-GST-1 belongs to a nematode-specific Nu class GST family that is different from human GST classes of protein (e.g., alpha, kappa, mu, omega, pi, theta, zeta and microsomal GSTs), both structurally and functionally. Nu class GSTs have diminished peroxidase activity compared to other classes of GSTs, but elevated binding capacity for heme and related products. The sequence homology between *Na*-GST-1 and GSTs expressed in human tissues ranges from 10-36%. The human protein that shares the highest (36%) amino acid homology with *Na*-GST-1 – GSTS (prostaglandin D synthase) – is expressed abundantly in placenta, lung and liver and moderately in heart and lymph node tissues. Since recombinant *Na*-GST-1 is being developed as a vaccine for humans, there is a theoretical concern that anti-*Na*-GST-1 antibodies induced by immunizing with the recombinant protein might cross-react with human GSTs expressed in human tissues. Western-blot assays using anti-*Na*-GST-1 and commercially-available anti-human GSTK1 (a protein expressed mostly in human liver, lung, thymus and lymph node tissues) antibodies were used to evaluate this potential cross-reactivity.

In these experiments, pooled immune sera from mice vaccinated with recombinant *Na*-GST-1 only recognized recombinant *Na*-GST-1 or native *Na*-GST-1 in the extracts of *N. americanus*

adult worms, but not human GST proteins (GSTK1, GSTT1 and GSTA1) or native GSTs in human tissue lysates of liver, brain, spleen, lung, heart, placenta, lymph node and thymus tissue. Likewise, monoclonal anti-human GSTK1 antibodies specifically recognized human GSTK1 protein or native GSTK1 expressed mainly in human liver, lung, heart, lymph node and thymus tissues, but not any recombinant *Na*-GST-1 or *N. americanus* adult extracts. Based upon the current data generated in these *in vitro* studies, it appears unlikely that antibodies induced by vaccination with recombinant *Na*-GST-1 will cross-react with GST proteins expressed in human tissue.

1.9 Clinical Experience with *Na*-GST-1

The recombinant *Na*-GST-1 protein is currently being tested in humans in a phase 1 trial of *Na*-GST-1/Alhydrogel® (with or without GLA-AF) in Brazil (protocol SVI-10-01), which was initiated in November 2011 and which will run in parallel with the study described in this protocol. The study in Brazil is testing *Na*-GST-1/Alhydrogel® when co-formulated with a single concentration of GLA-AF (2.5 µg).

Initial results of the phase 1 trial of *Na*-GST-1/Alhydrogel® (with or without GLA-AF) in Brazil indicate that in hookworm-naïve healthy adults, the vaccine is well tolerated and results in no significant adverse events. In 36 healthy, hookworm-unexposed Brazilian adults living in the urban center of Belo Horizonte, Brazil, and who received up to 3 injections of up to 100 µg *Na*-GST-1/Alhydrogel® with or without 2.5 µg GLA-AF (administered at 0, 2 and 4 months), the most common adverse events observed that were possibly-, probably-, or definitely related to vaccination were: injection site pain, injection site tenderness, headache, regional (axillary) lymphadenopathy, and myalgias. Most events were of mild to moderate intensity; three cases of injection site pain were graded as severe. In addition, preliminary immunogenicity results indicate that volunteers vaccinated with *Na*-GST-1/Alhydrogel® develop antigen-specific IgG antibodies in a dose-dependent fashion, with significant levels of antibody induced only after a third vaccination (**Figure 9**). Furthermore, the addition of 2.5 µg of GLA-AF did not appear to appreciably increase the induced levels of anti-*Na*-GST-1 IgG antibodies during the time period studied. It should be noted that this study is ongoing and that participants continue to be followed for long-term immunogenicity and occurrence of adverse events; the data presented above include only those collected from study participants up to and including 2 weeks following the final vaccination.

Figure 9: Geometric mean anti-*Na*-GST-1 IgG antibody units 2 weeks after the 3rd Immunization of Healthy Hookworm-Unexposed Brazilian Adults with *Na*-GST-1/Alhydrogel® with (A) or without (B) co-administration of 2.5 µg GLA-AF (SVI-10-01).

A

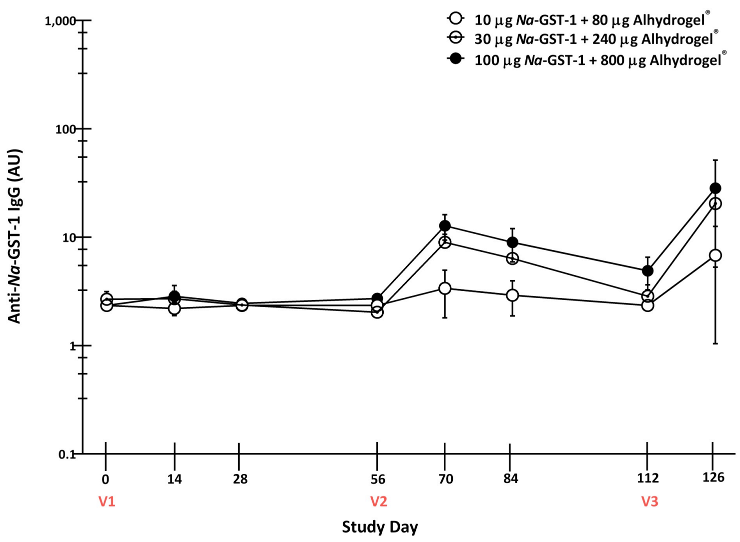

B

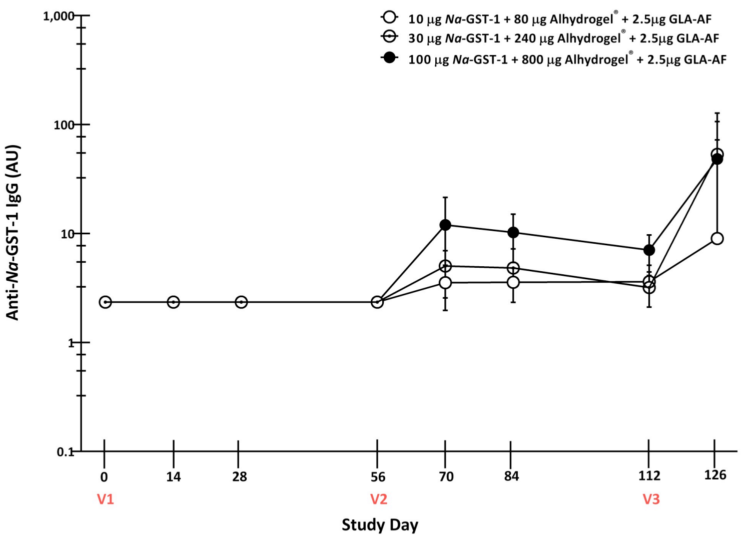

1.10 Clinical Experience with Aluminum-Based Adjuvants

Several licensed vaccines contain aluminum-based adjuvants, including the recombinant Hepatitis B vaccine (e.g., Recombivax®), the tetanus toxoid vaccine, and the diphtheria-tetanus toxoids vaccine.[37, 38] For these aluminum hydroxide-adsorbed vaccines, local reactions such as pain, tenderness, and swelling are experienced in between 7.6% and 16.7% of volunteers in studies that included over 1,200 healthy adults. Fever is seen in 3.2% to 9.3%, headache in 4.1%, and other systemic symptoms such as fatigue, malaise, nausea, and diarrhea at lower

frequencies. Urticaria has been reported in 0.1% of individuals vaccinated with the hepatitis B vaccine.

1.11 Clinical Experience with GLA-AF

Gluco-Pyranosylphospho-Lipid A Aqueous Formulation (GLA-AF, Infectious Diseases Research Institute [IDRI], Seattle, WA) contains a synthetic monophosphoryl lipid A (MPL) molecule that has TLR4 agonist activity. MPL is itself derived from the lipopolysaccharide (LPS) of *Salmonella minnesota*, a natural TLR4 agonist that is pyrogenic and can induce toxic shock. LPS, and more specifically, its lipid A component, has long been known for its strong adjuvant effects; however, its high toxicity has precluded its use in a vaccine formulation. Ribi et al[22] showed that the monophosphorylated form of lipid A retains its immunostimulant function and almost completely loses its endotoxin effects.

GLA-AF is currently being tested in a phase 1 study of *Na*-GST-1/Alhydrogel® in Brazil as described above in **Section 1.9** (SVI-10-01). To date in that study, 38 subjects have received a 2.5 µg dose of GLA-AF co-administered with 10, 30, or 100 µg *Na*-GST-1/Alhydrogel® with no serious adverse events reported. An oil-in-water emulsion of GLA (GLA-SE) has also been used in combination with the Fluzone® trivalent killed influenza vaccine in a Phase 1 trial. In this study, doses up to 1 µg of GLA-SE were safe and well-tolerated and significantly enhanced influenza-specific antibody responses (unpublished data provided by IDRI).

In addition, there have been many clinical trials involving thousands of participants in which MPL or a derivative have been administered as vaccine adjuvants to adults and children, including vaccines for human papillomavirus, malaria,[39-41] leishmaniasis,[42] and hepatitis B.[43] In general, these trials have demonstrated that administering MPL to humans is safe and well tolerated; when compared to formulations of vaccine that do not contain MPL, those adjuvanted with MPL may result in a minor increase in the incidence and/or severity of local injection site reactions. However, the addition of MPL also often results in a much improved specific antibody response to the vaccine antigen(s).

Of note, MPL is one of the components of the newly licensed Cervarix® vaccine (GlaxoSmithKline, Research Triangle Park, NC) for the prevention of cervical cancer due to human papillomavirus serotypes 16 and 18. The adjuvant for this vaccine consists of MPL adsorbed to aluminum hydroxide salt and is therefore similar to the combination of GLA-AF and Alhydrogel® that we propose testing in combination with *Na*-GST-1 in the study described in this protocol. The Cervarix® vaccine has been shown to have a very favorable safety profile after having been tested in tens of thousands of healthy females.[44, 45]

1.12 Clinical Development Plan

The phase 1 trial described in this protocol will enroll healthy adults who have not been infected with or exposed to hookworm who are living in the Washington DC area. A phase 1 trial of *Na*-GST-1/Alhydrogel (with or without GLA-AF) was initiated in Brazil in November 2011 with first dosing in January 2012 (the protocol [SVI-10-01] has been approved by the local Brazilian IRB, the George Washington University Medical Center IRB and the Brazilian national regulatory agency). In the study in Brazil, both hookworm-naïve and hookworm-exposed healthy adults will be vaccinated with either *Na*-GST-1/Alhydrogel®, *Na*-GST-1/Alhydrogel®

co-administered with 2.5 µg GLA-AF, or the Butang® hepatitis B vaccine. The objectives of this study will be to assess the safety and immunogenicity of the *Na*-GST-1 formulations in healthy Brazilian adults (initial safety and immunogenicity results are described above in **Section 1.9**). The study described in this protocol (SVI-11-01) will complement the study in Brazilian adults as it will provide additional information about different dose concentrations of GLA-AF as well as detailed data on the humoral immune response to the different formulations that will be tested.

Provided no safety concerns are observed during the two adult phase 1 studies that will be conducted concurrently in the United States (i.e., the study described in this protocol) and Brazil (SVI-10-01), age de-escalation, phase 2, and eventually phase 3 clinical trials will be undertaken in hookworm-endemic areas. A phase 1 trial of *Na*-GST-1 in Brazilian school-aged children is being planned for 2013, assuming no safety issues are identified in the adult study described in this protocol or the one in Brazil.

2 OBJECTIVES

2.1 Primary Objective

1. To determine the frequency of vaccine-related AEs, graded by severity, for each dose and formulation of *Na*-GST-1.

2.2 Secondary Objectives

1. To determine the dose and formulation that generates the highest anti-*Na*-GST-1 antibody response following vaccination.
2. To determine the dose, formulation, and number of injections of *Na*-GST-1 that generates the antibody response of greatest affinity.
3. To determine the dose and formulation of the *Na*-GST-1 vaccine that results in the most robust production of *Na*-GST-1 specific B cells and subtypes (memory or plasma).
4. To perform exploratory studies of the cellular immune responses to the *Na*-GST-1 antigen both before and after immunization.

3 STUDY SITE

The study will be conducted in healthy adult volunteers without history of hookworm infection at the clinical research unit of the Clinical Research Center located at Children's National Medical Center (CNMC) in Washington, DC, as well as at the Department of Microbiology, Immunology, and Tropical Medicine (MITM) of the George Washington University School of Medicine and Health Sciences. All screening and vaccination visits and visits that require collection of venous blood for clinical safety laboratory tests will be conducted at CNMC whereas all other follow-up visits may be conducted either at CNMC or MITM.

4 STUDY DESIGN

4.1 Overall Design

The study will be a phase 1 dose-escalating clinical trial in healthy adult volunteers to evaluate the safety, reactogenicity, and immunogenicity of *Na*-GST-1/Alhydrogel® with or without two different dose concentrations of GLA-AF (1 µg or 5 µg).

Safety parameters will be monitored throughout both parts of the study. The primary immunological endpoint for both parts will be the measurement of the antigen-specific antibody response.

Volunteers will be invited to participate in the study. After providing written informed consent, volunteers will undergo eligibility screening, including a complete medical history, physical examination, hematology and coagulation testing, serum glucose testing, liver and renal function testing, HIV antibody testing, Hepatitis B and C serology, and urinalysis; urine pregnancy testing will be performed on all female volunteers. All clinically significant abnormalities will be reviewed with volunteers and they will be referral for follow-up care if appropriate. After screening, those volunteers determined to be eligible, based on the inclusion and exclusion criteria described in **Section 5** in this protocol, will be invited to participate in the study.

In the study, 40 volunteers will be progressively enrolled into one of five dose cohorts. Dose cohorts will be enrolled and vaccinated in a consecutive fashion as described in **Section 4.3**. The 12 volunteers in each of cohorts 1 and 2 will be randomized in a 1:2 ratio to receive either the *Na*-GST-1/Alhydrogel® (n=4) or *Na*-GST-1/Alhydrogel®/GLA-AF (n=8). Volunteers in each of cohorts 3-5 will not be randomized and will all receive the same dose and formulation of *Na*-GST-1/Alhydrogel®. Volunteers will receive either 10 µg, 30 µg or 100 µg of *Na*-GST-1, as outlined in **Table 2**.

In the first and second cohorts, volunteers will be randomized to receive either *Na*-GST-1/Alhydrogel® (n=4) <u>OR</u> *Na*-GST-1/Alhydrogel® co-administered with 1 µg GLA-AF (n=8); doses will be administered in a double-blind fashion for these two cohorts. In the third, fourth, and fifth cohorts, volunteers will receive *Na*-GST-1/Alhydrogel® or *Na*-GST-1/Alhydrogel® co-administered with 5 µg GLA-AF in an open-label fashion, as shown in **Table 2**.

As with other aluminum hydroxide-adsorbed vaccines, hypersensitivity reactions would be expected to occur within the first 24 hours after receipt of either of the two vaccines, and other severe local or systemic reactions within 72 hours of vaccination. Participants will therefore be observed for immediate reactions following each vaccination for at least 2 hours, and will have a clinical assessment at the study clinic on Days 3, 7, and 14 following <u>each</u> vaccination. See **Tables 1** and **2** for tabular descriptions of the vaccination schedule for all dose cohorts, as well as **Section 7.5** and **Appendix A** for a detailed description of the scheduled clinical and laboratory evaluations.

Prior to dose escalation from the 1 µg dose of GLA-AF to the 5 µg dose of GLA-AF (after the first vaccination of Cohort 2 but before the first vaccination of Cohort 3), safety data up to and including Day 7 post-vaccination will be compiled from the lower dose cohorts for review by the

Medical Monitor and/or SMC. The trial will not proceed to the higher dose cohort if any of the stopping criteria listed in **Section 8.5** are met or, in the clinical judgment of the SMC and/or Medical Monitor, the higher dose would pose an unacceptable safety risk to the participants.

Table 1: Study Design

Cohort	Dose *Na*-GST-1	Cohort Characteristics	Total
1	10 µg	4 *Na*-GST-1/Alhydrogel® +<br>8 *Na*-GST-1/Alhydrogel®/GLA-AF(1 µg)	12
2	30 µg	4 *Na*-GST-1/Alhydrogel® +<br>8 *Na*-GST-1/Alhydrogel®/GLA-AF(1 µg)	12
3	30 µg	4 *Na*-GST-1/Alhydrogel®/GLA-AF(5 µg)	4
4	100 µg	4 *Na*-GST-1/Alhydrogel®	4
5	100 µg	8 *Na*-GST-1/Alhydrogel®/GLA-AF(5 µg)	8
**Total**		**12 *Na*-GST-1/Alhydrogel® +<br>28 *Na*-GST-1/Alhydrogel®/GLA-AF**	**40**

Table 2: Vaccination Schedule

**Time**[1] (Calendar Week)	**Cohort 1** (n = 12)<br><br>10 µg *Na*-GST-1<br>80 µg Alhydrogel®<br>(n=4)<br>**OR**<br>10 µg *Na*-GST-1<br>80 µg Alhydrogel® +<br>1 µg GLA-AF<br>(n=8)	**Cohort 2** (n = 12)<br><br>30 µg *Na*-GST-1<br>240 µg Alhydrogel®<br>(n=4)<br>**OR**<br>30 µg *Na*-GST-1<br>240 µg Alhydrogel®<br>+ 1 µg GLA-AF<br>(n=8)	**Cohort 3** (n = 4)<br><br>30 µg *Na*-GST-1<br>240 µg Alhydrogel®<br>+ 5 µg GLA-AF<br>(n=4)	**Cohort 4** (n = 4)<br><br>100 µg *Na*-GST-1<br>800 µg<br>Alhydrogel®<br>(n=4)	**Cohort 5** (n = 8)<br><br>100 µg *Na*-GST-1<br>800 µg Alhydrogel®<br>+ 5 µg GLA-AF<br>(n=8)
0	Vaccination 1 (Day 0)				
2		Vaccination 1 (Day 0)			
4			Vaccination 1 (Day 0)		
6				Vaccination 1 (Day 0)	Vaccination 1 (Day 0)
8	Vaccination 2 (Day 56)				
10		Vaccination 2 (Day 56)			
12			Vaccination 2 (Day 56)		
14				Vaccination 2 (Day 56)	Vaccination 2 (Day 56)
16	Vaccination 3 (Day 112)				
18		Vaccination 3 (Day 112)			

20			Vaccination 3 (Day 112)			
22				Vaccination 3 (Day 112)	Vaccination 3 (Day 112)	

[1]Minimum time delay between cohorts (see **Section 7.5** for more detail)

4.2 Sample Size and Estimated Duration of Study

A total of 40 participants will be enrolled (see **Section 10.2** for a justification of the sample size). Twelve will receive *Na*-GST-1/Alhydrogel® (one of three dose concentrations) without GLA-AF, 16 will receive *Na*-GST-1/Alhydrogel® (one of two dose concentrations) co-administered with 1 µg GLA-AF, and 12 will receive *Na*-GST-1/Alhydrogel® (one of two dose concentrations) co-administered with 5 µg GLA-AF. The trial is expected to last for approximately 24 months. Each participant will be followed for 16 months from the time of the first injection.

4.3 Group Allocation

The five dose cohorts will be enrolled consecutively. Within Cohorts 1 and 2, of 12 participants each, 4 will be randomly assigned to receive *Na*-GST-1/Alhydrogel® and 8 to receive *Na*-GST-1/Alhydrogel® co-administered with 1 µg GLA-AF, with the dose of *Na*-GST-1 being 10 and 30 µg for all participants within each cohort, respectively (see **Section 7.2**). Within Cohorts 3 and 4 of 4 participants each, participants will receive 30 µg *Na*-GST-1/Alhydrogel® co-administered with 5 µg GLA-AF and 100 µg *Na*-GST-1/Alhydrogel®, respectively. Finally, in Cohort 5, 8 participants will receive 100 *Na*-GST-1/Alhydrogel® co-administered with 5 µg GLA-AF.

Once screening has started and the first 15 participants have been deemed eligible, they will be assigned to Cohort 1, and a Day 0 visit will be scheduled for each one (this may not be on the same date for all 15). On the day of vaccination, the first 12 who present to the study clinic will be evaluated for continued eligibility; the remaining 3 will be kept as alternates if some of the first 12 cannot be vaccinated on the day of first vaccination (i.e., due to pregnancy, withdrawal of consent, etc.). If the alternates are not vaccinated, they will be invited to participate as members of the next dose cohort. The 12 eligible participants will be randomly assigned to receive either *Na*-GST-1 (10 µg)/Alhydrogel® (n=4) or *Na*-GST-1 (10 µg)/Alhydrogel® co-administered with 1 µg GLA-AF (n=8). Following assembly of the first dose cohort, the next 15 eligible participants will be assigned to Cohort 2 and their Day 0 visit scheduled using the same procedure as described for the first dose cohort. The 12 eligible participants of the second cohort will be randomly assigned to receive either *Na*-GST-1 (30 µg)/Alhydrogel® (n=4) or *Na*-GST-1 (30 µg)/Alhydrogel® co-administered with 1 µg GLA-AF (n=8). Those alternates for the second cohort who are not vaccinated will be invited to participate as members of the next dose cohort. Enrollment of the third, fourth, and fifth cohorts will proceed in a similar fashion.

4.4 Blinding for Cohorts 1 and 2

For Cohorts 1 and 2 only, investigators and participants will be blinded as to an individual study participant's allocation to either *Na*-GST-1/Alhydrogel® or *Na*-GST-1/Alhydrogel®/GLA-AF until all participants have completed their Day 140 visit, the safety and primary immunogenicity data have been monitored and entered into the database, and the database has been locked. Only the study vaccine manager(s) will be aware of the participants' vaccine allocation. The study

vaccine manager will refer to the unique randomization code assigned to that participant to determine the assigned vaccine formulation for each participant (see **Section 7.2**). Vaccine doses will be prepared by the study vaccine manager in a separate room and the vaccine-filled syringes will be delivered to the vaccinator(s). Since the doses of the *Na*-GST-1/Alhydrogel® formulations will be of slightly different volumes than the *Na*-GST-1/Alhydrogel®/GLA-AF formulations, the contents of all syringes for Cohorts 1 and 2 will be disguised using opaque tape.

The investigators will be unblinded to participants' vaccine allocation status after all Cohort 1 and 2 participants have had their study Day 140 visit (see **Section 8.10**). After this point, the study will be single-blinded (i.e., study participants in Cohorts 1 and 2 will remain blinded to what vaccine they have received).

For Cohorts 3, 4 and 5, vaccines will be administered in an unblinded, open fashion. That is, both investigators and participants will know which dose of *Na*-GST-1 and GLA-AF that they have received. This represents a modification from the original design of this study and has been prompted by interim safety and immunogenicity results from an ongoing phase 1 clinical trial of the vaccine in Brazil as discussed in **Section 1.9** above. In this study, doses of up to 100 µg *Na*-GST-1 (with or without 2.5 µg GLA-AF) were well tolerated, but the addition of 2.5 µg GLA-AF did not appreciable increase the antigen-specific antibody response. Since adequate safety data has been obtained from the current trial and the one in Brazil, it is felt that reducing the size of the remaining cohorts and administering vaccinations in an unblinded fashion is justified.

5 SELECTION OF PARTICIPANTS

5.1 Inclusion Criteria

1. Males or females between 18 and 45 years, inclusive.
2. Good general health as determined by means of the screening procedure.
3. Available for the duration of the trial (16 months).
4. Willingness to participate in the study as evidenced by signing the informed consent document.

5.2 Exclusion Criteria

1. Pregnancy as determined by a positive urine hCG (if female).
2. Participant unwilling to use reliable contraception methods up until one month following the third immunization (if female).
3. Currently lactating and breast-feeding (if female).
4. Evidence of clinically significant neurologic, cardiac, pulmonary, hepatic, rheumatologic, autoimmune, diabetes, or renal disease by history, physical examination, and/or laboratory studies.
5. Known or suspected immunodeficiency.
6. Laboratory evidence of liver disease (alanine aminotransferase [ALT] greater than 1.25-times the upper reference limit).
7. Laboratory evidence of renal disease (serum creatinine greater than 1.25-times the upper reference limit, or more than trace protein or blood on urine dipstick testing).

8. Laboratory evidence of hematologic disease (hemoglobin <11.0 g/dl [females] or <12.2 g/dl [males]; absolute leukocyte count <3500/mm^3 or >10.5 x 10^3/mm^3; absolute neutrophil count [ANC] <1700/ mm^3; absolute lymphocyte count <1100/mm^3; or platelet count <140,000/mm^3).
9. Laboratory evidence of a coagulopathy (PTT or PT INR greater than 1.1-times the upper reference limit).
10. Serum glucose (random) greater than 1.2-times the upper reference limit.
11. Other condition that in the opinion of the investigator would jeopardize the safety or rights of a volunteer participating in the trial or would render the subject unable to comply with the protocol.
12. Participation in another investigational vaccine or drug trial within 30 days of starting this study.
13. Volunteer has had medical, occupational, or family problems as a result of alcohol or illicit drug use during the past 12 months.
14. History of a severe allergic reaction or anaphylaxis.
15. Severe asthma as defined by the need for regular use of inhalers or emergency clinic visit or hospitalization within the last 6 months.
16. Positive ELISA for HBsAg.
17. Positive ELISA and confirmatory Western blot tests for HIV.
18. Positive ELISA and confirmatory immunoblot tests for HCV.
19. Use of corticosteroids (excluding topical or nasal) or immunosuppressive drugs within 30 days of starting this study.
20. Receipt of a live vaccine within past 4 weeks or a killed vaccine within past 2 weeks prior to entry into the study.
21. History of a surgical splenectomy.
22. Receipt of blood products within the past 6 months.
23. History of allergy to yeast.
24. History of previous infection with hookworm or residence for more than 6 months in a hookworm-endemic area.

6 VACCINE PREPARATION

6.1 Supplies

6.1.1 Na-*GST-1/Alhydrogel®*

Na-GST-1/Alhydrogel® is supplied as a sterile milky-white suspension (when shaken slightly). Each 2.0 ml vial contains 1.35 ml of a 0.1 mg/ml suspension of *Na*-GST-1 adsorbed to 0.8 mg/ml of Alhydrogel® in a buffer consisting of 10% glucose and 10 mM imidazole, pH 7.4. Glucose acts as an excipient and imidazole as the buffer based on evidence that these components specifically enhance the stability and solubility of *Na*-GST-1. The maximum dose that will be administered is 100 µg of *Na*-GST-1, or 1.0 ml of the final drug product. This volume contains the equivalent of approximately 400 µg aluminum. Lower doses of *Na*-GST-1 will be delivered by injecting smaller volumes of the 0.1 mg/ml suspension: for example, 0.3 ml will be injected to deliver 30 µg *Na*-GST-1 and 0.1 ml to deliver 10 µg *Na*-GST-1. For all doses, the ratio of *Na*-GST-1 to Alhydrogel® will therefore remain constant: for the 10, 30 and 100 µg

doses of *Na*-GST-1 the respective amounts of Alhydrogel® will be 80, 240 and 800 µg (corresponding to approximately 40, 120 and 400 µg aluminum, respectively). *Na*-GST-1/Alhydrogel® was manufactured, formulated and vialed at Aeras Global Vaccine Foundation (Rockville, Maryland, USA). The product conforms to established requirements of purity, sterility, safety, and identity.

6.1.2 GLA-AF

GLA-AF will be supplied to the trial site as a 100 µg/mL aqueous solution in multi-dose vials containing 40 µg of GLA without preservative. Each vial contains a total volume of 0.4 mL. Appropriate volumes of GLA-AF will be withdrawn and added to a vial containing 1.35 mL of *Na*-GST-1/Alhydrogel® or vice versa (described in the Investigator's Brochure). The mixture must be administered not less than 30 minutes before and not more than 24 hours after mixing the GLA-AF with *Na*-GST-1/Alhydrogel®.

6.2 Transport of Vaccines

Vials of *Na*-GST-1/Alhydrogel® and GLA-AF will be supplied to the study site by the Sponsor. Vials of *Na*-GST-1/Alhydrogel® and GLA-AF will be transported to the study site at 1°C to 9°C; temperature recording devices will accompany the vaccines at all times to ensure temperature limits during transport have not been violated. Vials of *Na*-GST-1/Alhydrogel® and GLA-AF will be stored at the study site in a refrigerator at 2°C to 8°C and must not be frozen; refrigerator temperature should be monitored continuously. All vials of *Na*-GST-1/Alhydrogel® and GLA-AF must be stored in the upright position.

6.3 Preparation of Vaccine Doses

The *Na*-GST-1/Alhydrogel® plus GLA-AF formulations will be prepared by adding an appropriate volume of the GLA-AF solution (100 µg/ml) to a vial of *Na*-GST-1/Alhydrogel®, or vice versa, within 24 hours of vaccination.

6.4 Vaccine Storage

Na-GST-1/Alhydrogel® and GLA-AF should be maintained at 2°C to 8°C until just prior to administration. *Na*-GST-1/Alhydrogel® and GLA-AF should NOT be frozen at any time.

6.5 Vaccine Accountability

Study *Na*-GST-1/Alhydrogel® and GLA-AF supplies must be received by a designated person at the study site, handled and stored safely and properly, and kept in a secure location to which only the Investigator and designated assistants have access. Study-site personnel are responsible for maintaining accurate records of the vaccine supplies (i.e., *Na*-GST-1/Alhydrogel® and GLA-AF) received, the quantities administered to study participants, and the amounts remaining at the conclusion of the study.

6.6 Disposition of Used/Unused Supplies

After administration of *Na*-GST-1/Alhydrogel® or GLA-AF doses, vials will be stored at the study site, and vials will be accounted for and stored until monitoring by the study Sponsor or their designee. The vials may then be disposed of according to site protocol. At the conclusion of the study, all unused *Na*-GST-1/Alhydrogel® and GLA-AF supplies will be destroyed on site upon direction from the Sponsor, returned to the Sponsor, or maintained at 2 to 8°C until further notice from the Sponsor regarding their disposition. All other vials will be retained until requested by the Sponsor.

7 STUDY PROCEDURES

The following sections provide a detailed listing of the procedures and studies to be performed in this protocol at designated time points. The total volume of blood (approximately 510 mL) to be collected from each volunteer over their 16-month participation in the trial is approximately the volume collected when donating one unit of blood and should not compromise the health of trial participants.

7.1 Individual Recruitment and Informed Consent

Volunteers aged 18-45 years, inclusive, will be invited to undergo screening for the study. During this initial screening visit, a member of the study team will read the consent form together with the volunteer. The study team member will explain why the volunteer is being invited to participate in the study and will clarify all of the volunteer's questions. Volunteers will be encouraged to ask questions. The volunteer may either sign the consent form immediately or later after further consideration.

The following procedures will be performed upon initial screening (note that all procedures might not be performed on the same day):

1. Explain the study and Informed Consent to the volunteer.
2. Elicit a complete medical history, including medication history, and for female subjects, a menstrual and contraceptive history and/or history of surgical sterility.
3. Administer a complete physical examination.
4. Obtain blood for hematology, coagulation tests, biochemistry, and serologic tests for HIV and viral hepatitis (B and C).
5. Obtain urine for urine dipstick testing, as well as urine hCG testing in females.
6. Counsel females to avoid becoming pregnant during the study.

Screening steps 2-6 must be performed within 100 days of the planned enrollment into the study. Should this screening window be exceeded before the first vaccination, screening procedures (not including administration of the informed consent form) may be repeated to ensure continued eligibility for the study (screening blood tests may be repeated a maximum of one time).

7.2 Inclusion

Eligible volunteers will be asked to come to the study clinic on their scheduled day of enrollment into the study. After undergoing a clinical interview and exam to ensure that they remain

eligible for participation in the study, that they have had blood collected for the studies outlined in **Section 7.5**, and that females have had a urine pregnancy test performed on the day of vaccination that is documented to be negative, volunteers will be assigned a unique study number and vaccinated as described in **Section 7.4**.

For Cohorts 1 and 2 only, randomization to either *Na*-GST-1/Alhydrogel® or *Na*-GST-1/Alhydrogel®/GLA-AF will be done through use of a randomization code furnished to the study vaccine manager by the Sponsor. The study numbers will be assigned in the order in which the participants are enrolled in the study, so that among the first 12 study numbers of the study, 4 will be assigned to *Na*-GST-1(10 µg)/Alhydrogel® and 8 to *Na*-GST-1(10 µg)/Alhydrogel®/GLA-AF(1 µg). Assignment of the study numbers will be done on the day of first vaccination, in the order that the study participants present for immunization. Access to the randomization list will be exclusively limited to the study vaccine manager(s). Between vaccination days, the randomization list will be stored in a sealed envelope in a locked cabinet. The study vaccine manager(s) will be unblinded, but will not be involved in study participants' further evaluation.

For Cohorts 3, 4, and 5, all study participants in each of these cohorts will receive the same dose and formulation of vaccine; therefore, there is no need to randomize and vaccines will be delivered in an unblinded fashion.

7.3 Enrollment

Volunteers will not be considered enrolled in the study until they have received their first dose of vaccine. In the event that a volunteer is randomized but not enrolled on the day of first vaccination, they will be replaced with an eligible alternate.

7.4 Immunization Procedure

Participants will receive 3 immunizations, on Days 0, 56, and 112. *Na*-GST-1/Alhydrogel® and GLA-AF supplies will be kept refrigerated at 2°C to 8°C until just before use, whereupon they will be warmed to room temperature. Vaccinations will be delivered by intramuscular injection in the deltoid muscle with a single-use needle of appropriate length after preparation of the site with alcohol. Successive vaccinations will be given in alternating arms.

7.5 Clinical Monitoring and Evaluation

See **Appendix A** for a tabular representation of study procedures.

<u>Study Day 0 (Day of First Vaccination)</u>

1. Verify that Informed Consent was obtained.
2. Verify that all applicable eligibility criteria have been met.
3. Perform abbreviated history (including concomitant medications) and physical exam, focusing on any acute complaints.
4. Obtain blood for hematology, biochemistry, anti-GST-1 antibody ELISA and cellular immunology assays.
5. For females, obtain a urine sample for hCG testing. Ensure that test is negative before proceeding; a positive test will exclude the volunteer from the trial.

6. Record vital signs (blood pressure, oral temperature, and heart rate).
7. Administer the vaccine.
8. Observe for at least 2 hours after vaccination to evaluate for immediate adverse reactions. During the 2-hour post-immunization wait period, study staff will discuss signs and symptoms of potential AEs, describe proper use of digital thermometers, injection-site reaction measurement tools, and participant symptom diaries.

Study Day 3 +/- 1

1. Perform basic history and physical exam (including injection site), emphasizing examination of any acute complaints.
2. Record vital signs.
3. Collect participant symptom diary card.

Study Day 7 +/- 1

1. Perform basic history and physical exam (including injection site), emphasizing examination of any acute complaints.
2. Record vital signs.

Study Day 14 +/- 2

1. Perform basic history and physical exam (including injection site), emphasizing examination of any acute complaints.
2. Record vital signs.
3. Obtain blood for hematology, biochemistry, anti-GST-1 antibody ELISA, and cellular assays.

Study Day 28 +/- 4

1. Perform basic history and physical exam (including injection site), emphasizing examination of any acute complaints.
2. Record vital signs.
3. Obtain blood for anti-GST-1 antibody ELISA and cellular assays.

Study Day 56 +/- 7 (Day of Second Vaccination)

1. Perform basic history (including concomitant medications) and physical exam (including injection site), emphasizing examination of any acute complaints.
2. Obtain blood for hematology, biochemistry, anti-GST-1 antibody ELISA, and cellular immunology assays.
3. For females, obtain a urine sample for hCG testing. Ensure that test is negative before proceeding; a positive test will exclude the volunteer from further vaccinations.
4. Record vital signs.
5. Administer the vaccine.
6. Observe for at least 2 hours after vaccination to evaluate for immediate adverse reactions. During the 2-hour post-immunization wait period, study staff will discuss signs and symptoms of potential AEs, describe proper use of digital thermometers, injection-site reaction measurement tools, and participant symptom diaries.

Study Day 59 (3 +/- 1 days after Second Vaccination)
1. Perform basic history and physical exam (including injection site), emphasizing examination of any acute complaints.
2. Record vital signs.

Study Day 63 (7 +/- 1 days after Second Vaccination)
1. Perform basic history and physical exam (including injection site), emphasizing examination of any acute complaints.
2. Record vital signs.
3. Collect participant symptom diary card.

Study Day 70 (14 +/- 2 days after Second Vaccination)
1. Perform basic history and physical exam (including injection site), emphasizing examination of any acute complaints.
2. Record vital signs.
3. Obtain blood for hematology, biochemistry, anti-GST-1 antibody ELISA, and cellular immunology assays.

Study Day 84 (28 +/- 4 days after Second Vaccination)
1. Perform basic history and physical exam, emphasizing examination of any complaints.
2. Record vital signs.
3. Obtain blood for anti-GST-1 antibody ELISA and cellular assays.

Study Day 112 +/- 14 (Day of Third Vaccination)
1. Perform basic history (including concomitant medications) and physical exam (including injection site), emphasizing examination of any acute complaints.
2. Obtain blood for hematology, biochemistry, anti-GST-1 antibody ELISA, and cellular immunology assays.
3. For females, obtain a urine sample for hCG testing. Ensure that test is negative before proceeding; a positive test will exclude the volunteer from further vaccinations.
4. Record vital signs.
5. Administer the vaccine.
6. Observe for at least 2 hours after vaccination to evaluate for immediate adverse reactions. During the 2-hour post-immunization wait period, study staff will discuss signs and symptoms of potential AEs, describe proper use of digital thermometers, injection-site reaction measurement tools, and participant symptom diaries.

Study Day 115 (3 +/- 1 days after Third Vaccination)
1. Perform basic history and physical exam (including injection site), emphasizing examination of any acute complaints.
2. Record vital signs.

Study Day 119 (7 +/- 1 days after Third Vaccination)
1. Perform basic history and physical exam (including injection site), emphasizing examination of any acute complaints.
2. Record vital signs.

3. Collect participant symptom diary card.

Study Day 126 (14 +/- 2 days after Third Vaccination)
1. Perform basic history and physical exam (including injection site), emphasizing examination of any acute complaints.
2. Record vital signs.
3. Obtain blood for hematology, biochemistry, anti-GST-1 antibody ELISA, and cellular immunology assays.

Study Day 140 (28 +/-4 days after Third Vaccination)
1. Perform basic history and physical exam, emphasizing examination of any acute complaints.
2. Obtain blood for anti-GST-1 antibody ELISA and cellular immunology assays.

Study Day 170 (2 months +/-14 days after Third Vaccination)
1. Perform basic history and physical exam, emphasizing examination of any acute complaints.
2. Obtain blood for anti-GST-1 antibody ELISA.

Study Day 230 (4 months +/-14 days after Third Vaccination)
1. Perform basic history and physical exam, emphasizing examination of any acute complaints.
2. Obtain blood for anti-GST-1 antibody ELISA.

Study Day 290 (6 months +/-14 days after Third Vaccination)
1. Perform basic history and physical exam, emphasizing examination of any acute complaints.
2. Obtain blood for anti-GST-1 antibody ELISA and cellular immunology assays.

Study Day 470 (12 months +/- 21 days after Third Vaccination)
1. Perform basic history and physical exam, emphasizing examination of any acute complaints.

7.6 Participant Symptom Diary

Study participants will be asked to keep daily symptom diaries recording oral temperature at two different times during the day, as well as pain, tenderness, erythema, and swelling at the injection site, and headache, nausea, vomiting, myalgias and arthralgias for 7 days following each immunization. The size of any injection-site reaction will be measured using a standardized measurement device that will be provided to the participant and recorded in the participant's symptom diary.

7.7 Laboratory Testing

Using standard techniques, the following tests will be performed at the clinical laboratory of Children's National Medical Center.

1. Complete blood count plus white blood cell (WBC) differential*
2. Serum creatinine
3. Alanine aminotransferase (ALT)
4. Serum glucose

5. Prothrombin time - International Normalized Ratio (PT INR)
6. Partial thromboplastin time (PTT)
7. HBsAg ELISA
8. HCV ELISA and immunblot
9. HIV ELISA and Western blot

**The following CBC parameters will be assessed for safety throughout the trial: WBC, absolute neutrophil count, hemoglobin concentration and platelet count.*

Urine hCG testing will be performed at the clinical trial site using urine pregnancy test kits that have been approved by the FDA. Urine dipstick testing will also be performed at the trial site using an approved product.

Anti-*Na*-GST-1 ELISAs, memory B cell assays, and cellular immunology assays will be performed at the CIL laboratory of the Department of Microbiology, Immunology, and Tropical Medicine at GWUMC. ImmunoCAP and Surface Plasmon Resonance (Biacore) assays will be performed at the Dermatology, Allergy, and Clinical Immunology (DACI) Reference Laboratory at Johns Hopkins University.

7.8 Immunologic Testing

*7.8.1 Anti-*Na*-GST-1 Antibody Assays*

Antibodies to *Na*-GST-1 will be measured in serum or plasma of study subjects using an indirect ELISA and/or a custom ImmunoCAP (Phadia, Inc.) assay. Antigen-specific IgG and IgE antibody levels will be measured by both methods at baseline and at several time-points post-vaccination. For the indirect ELISA method, microwell plates will be adsorbed with purified recombinant *Na*-GST-1, blocked, and incubated with test sample sera or plasma, and incubated overnight at approximately 4°C. Plates will then be washed and a horseradish peroxidase conjugated anti-human antibody (e.g., anti-human IgG) will be added and incubated with plates. Plates will be washed again, incubated with a chromogenic substrate, and then the Optical Density (OD) of the samples read using an automated ELISA plate reader. Levels of antibodies against *Na*-GST-1 in sera or plasma will be converted to Arbitrary Units of antibody by homologous interpolation of their OD readings into a Standard Reference Curve derived from serial dilutions of a positive "Standard Reference Serum" unique to each antibody isotype. A "Reactivity Threshold" (to determine seropositivity) will be set by the interpolated intersection of the upper 95% Confidence Interval (CI) of the Standard Reference Curve's lower asymptote with the lower 95% CI of the same asymptote.

The HP-series monoclonal anti-human IgG1-Fc (HP6070), anti-IgG2-Fc (clone HP6002), anti-IgG3-hinge (clone HP6047) and IgG4-Fc (clone HP6023) specific reagents from the Hybridoma Reagent Laboratory (Baltimore, MD) will be used to characterize the subclass distribution of the IgG antibody response in a biotinylated form of these subclass-specific reagents in an indirect ELISA assay system to characterize the subclass distribution of the IgG antibody response. In this assay, immobilized *Na*-GST-1 will bind anti-*Na*-GST-1 antibody in different dilutions of serum and the subclass distribution of bound IgG will be detected with the biotinylated IgG subclass specific monoclonal antibodies.

7.8.2 ImmunoCAP for IgG, IgG4 and IgE to **Na-*GST-1***

We will employ the ImmunoCAP 250 which is an FDA-approved autoanalyzer produced by Phadia, Inc. (Kalamazoo, MI). *Na*-GST-1 antigen will be covalently coupled to the sponge-based solid phase matrix in the form of a small cap. The *Na*-GST-1 protein will be sent to Phadia for coupling since the method for doing so is proprietary. Once the sera and *Na*-GST-1 caps are available, analysis will be performed using buffers and the human IgE, IgG or IgG4 specific conjugates and calibrators according to the documented procedures required by Phadia. Results will be reported in nanograms/ml of anti-*Na*-GST-1 IgE or micrograms/ml of anti-*Na*-GST-1 IgG or IgG4.

7.8.3 Surface Plasmon Resonance (Biacore)

The Johns Hopkins DACI Laboratory is equipped with a Biacore-3000 instrument that is designed with highly developed software to perform affinity determinations on sera collected in the study described in this protocol. First, optimal covalent coupling conditions for *Na*-GST-1 to a CM5 chip will be determined using the pH scouting program. Since affinity determinations are best performed with limited concentrations of *Na*-GST-1 on the chip, the second step will be to define the optimal concentration of immobilized *Na*-GST-1 to obtain optimal K-on and K-off binding rate constants for several anti-*Na*-GST-1 containing sera. Once the *Na*-GST-1-chip and regeneration conditions have been optimized, sequential sera from study subjects receiving different dose combinations of *Na*-GST-1 and GLA-AF will be systematically evaluated. For each serum sample, at least 8 to 12 dilutions of the serum sample containing anti-*Na*-GST-1 antibodies will be injected across the surface of the sensor chip (each in its own cycle) and changes in the index of refraction at the surface, where the antibody-antigen binding interaction occurs, will be measured as Resonance Units.

The antibody concentration injected into the Biacore instrument will be determined by ImmunoCAP analysis described above. With the resonance unit traces from the affinity analysis, various fitting algorithms will be used, such as the 1:1 Langmuir binding model that are built into the Biacore 3000 System software to compute an overall equilibrium binding constant. This analysis will enable determination of a variety of thermodynamic constants for anti-*Na*-GST-1 antibodies in the serum from subjects in different vaccine groups and assess the relative avidity of serum antibodies at different times after vaccine administration to participants in the different study cohorts.

The dose, formulation, and number of injections of *Na*-GST-1 that generates the IgG antibody response of greatest avidity will also be determined by Surface Plasmon Resonance. Here, the average affinity at equilibrium (K), which is the value of the association constant when one half of the antibody binding sites are filled, will be applied. Using the simple 1:1 Langmuir binding model for antibody:antigen interactions, the affinity constant will be computed as a ratio of the forward and reverse rate binding constants ($K = k_a/k_d$). The reliability of the calculated affinity constants is directly dependent on the reliability of the kinetic constants, the measure of antibody introduced into the system and the appropriateness of the fitting model employed. We expect overall antibody affinity to increase (or mature) from low (1×10^8 M^{-1}) to higher levels of 1×10^{12} M^{-1} with increasing number of immunizations, with the addition of GLA-AF and with higher concentrations of *Na*-GST-1.

7.8.4 Detection and quantification of memory B cells to Na-*GST-1*

In brief, blood from each volunteer will be collected in a heparanized Vacutainer® tube. Recombinant *Na*-GST-1 protein will be biotinylated and incubated with the whole blood sample. The biotinylated samples will then incubated at room temperature with the corresponding antibodies for detection of *Na*-GST-1 specific B cells (Streptavidin-FITC + CD20-PE + CD19-PECy5), activated B cells (Streptavidin FITC + CD80 PE + CD19 PECy5), memory B cells (Streptavidin-FITC + CD27-PE + CD19-PECy5), and plasma cells (Streptavidin-FITC + CD38-PE + CD19 PECy5). As controls, tubes for *Na*-GST-1 (Streptavidin-FITC) and CD19 (CD19-PECy5) will also be included. The samples will then be lysed and centrifuged. The supernatant will be discarded and samples washed with PBS+5% FSA and centrifuged. The supernatant will be discarded and FACS Fixing Solution added for read-out of 100,000 counts on a flow cytometer.

7.8.5 Detection of Cytokines and Chemokines

Cytokines and chemokines are to be evaluated through the use of BD™ CBA kits, including the Human Th_1/Th_2 Cytokine Kit for detection of IL-2, IL-4, IL-5, IL-10, TNF-α and IFN-γ and the Human Inflammatory Cytokine Kit for detection of IL-8, IL-1β, IL-6, IL-10, TNF and IL-12p70. These will be performed as per manufacturer's instructions.

7.9 Storage of Participant Specimens

Extra samples of serum, plasma, and PBMCs will be stored in case future questions arise regarding the safety or immune response to the vaccine. Extra lymphocytes will be cryopreserved for future development of human monoclonal antibodies to be used for future immunoassays. Prior to conducting any future tests on stored samples that are not described in this protocol, IRB permission will first be obtained.

8 Adverse Events Monitoring and Reporting

8.1 Definitions

8.1.1 Adverse Event

An adverse event (AE) includes any noxious, pathological or unintended change in anatomical, physiological or metabolic functions as indicated by physical signs, symptoms and/or laboratory-detected changes occurring in any phase of the clinical study, whether associated with the study vaccine or active comparator, and whether or not considered vaccination related. This includes an exacerbation of pre-existing conditions and intercurrent illnesses. All AEs must be graded for severity and relationship to the study vaccine as described in **Section 8.2.2** and **Section 8.2.3** in this protocol.

8.1.2 Serious Adverse Event (SAE)

An SAE is an AE, whether considered related to the study vaccine or not, meeting one of the following conditions:

1. Death during the period of protocol-defined surveillance

2. Life threatening: defined as an event that places a subject at immediate risk of death at the time of the event and does not refer to an event that hypothetically might have caused death were it more severe
3. Hospitalization during the period of protocol-defined surveillance: defined as at least an overnight stay in the hospital or emergency ward for treatment that would have been inappropriate if administered in the outpatient setting
4. Results in a congenital anomaly or birth defect
5. Results in a persistent or significant disability or incapacity: defined as a substantial disruption of the study participant's ability to carry out normal life functions
6. Any other important medical event that may not result in death, be life threatening, or require hospitalization, may be considered a serious AE when, based upon appropriate medical judgment, the event may jeopardize the subject and may require medical or surgical intervention to prevent one of the outcomes listed above.

8.1.3 Adverse Events of Special Interest (AESIs)

Special attention will be paid to monitoring for the occurrence of certain adverse events termed, "Adverse Events of Special Interest" or AESIs. These include inflammatory and autoimmune disorders that may potentially be related to the use of an immunostimulatory adjuvant (although none have been associated with the use of GLA-AF). The occurrence of the following AESI's will be closely monitored:

- Neuroinflammatory disorders (optic neuritis, multiple sclerosis, demyelinating disease, transverse myelitis, Guillain-Barre syndrome, myasthenia gravis, encephalitis, neuritis, Bell's palsy)
- Musculoskeletal disorders (systemic lupus erythematosus, cutaneous lupus, Sjogren's syndrome, scleroderma, dermatomyositis, polymyositis, rheumatoid arthritis, juvenile rheumatoid arthritis, polymyalgia, rheumatica, reactive arthritis, psoriatic arthropathy, ankylosing spondylitis, spondylarthropathy)
- Gastrointestinal disorders (Crohn's disease, ulcerative colitis, celiac disease)
- Metabolic diseases (autoimmune thyroiditis, Grave's or Basedow's disease, Hashimoto thyroiditis, insulin-dependent diabetes mellitus [IDDM], Addison's disease)
- Skin disorders (psoriasis, vitiligo, Raynaud's phenomenon, erythema nodosum, autoimmune bullous skin diseases)
- Others (autoimmune hemolytic anemia, idiopathic thrombocytopenic purpura, antiphospholipid syndrome, temporal arteritis, Behcet's syndrome, pernicious anemia, autoimmune hepatitis, primary biliary cirrhosis, primary sclerosing cholangitis, autoimmune glomerulonephritis, autoimmune uveitis, autoimmune cardiomyopathy, sarcoidosis, Stevens-Johnson syndrome).
- Vasculitides

8.2 Assessment of Adverse Events

8.2.1 Identification of AEs

Assessment of safety will include clinical observation and monitoring of hematological, blood chemistry, and immunologic parameters. Safety will be evaluated by monitoring of participants

for local and systemic adverse reactions during the course of the trial. Participants will be closely monitored for at least 2 hours following each immunization. Additionally, participants will be evaluated on Days 3, 7, 14 and 28 following each vaccination for clinical assessments.

All AEs will be graded for severity and relationship to study vaccine. Reactions will be graded as described in **Section 8.2.2**. Should a participant report an AE, it will be fully documented in their study chart, and discussed with the Principal Investigator.

8.2.2 Determination of Severity

All AEs will be assessed by the investigator using the following protocol-defined grading system:

Grade 0:	None
Grade 1:	Mild – No effect on activities of daily living; no medical intervention/therapy required
Grade 2:	Moderate – Partial limitation in activities of daily living (can complete ≥50% of baseline); no or minimal medical intervention/therapy required
Grade 3:	Severe – Activities of daily living limited to < 50% of baseline; medical evaluation/therapy required
Grade 4:	Potentially life threatening

Intensity of the following AEs will be assessed by the investigator as described in **Table 3**. All laboratory AEs will be graded in severity following the toxicity table in **Appendix B**. Unsolicited adverse events and clinically significant laboratory abnormalities not described in **Table 3** or **Appendix B** will be graded according to **Appendix C**.

Table 3: Assessment of Solicited Adverse Event Severity

Adverse Event	Grade	Severity
Pain at injection site	0	None
	1	Easily tolerated, does not interfere with activity
	2	Repeated use of non-narcotic pain reliever for > 24 hours or interferes with daily activity
	3	Any use of narcotic pain reliever or prevents daily activity
	4	Emergency room (ER) visit or hospitalization
Tenderness at injection site	0	None
	1	Mild discomfort to touch
	2	Discomfort with movement
	3	Significant discomfort at rest
	4	ER visit or hospitalization
Erythema at injection site	0	<25 mm
	1	25 mm - 50 mm
	2	51mm - 100 mm
	3	> 100 mm
	4	Necrosis or exfoliative dermatitis
Induration/swelling at injection site	0	<25 mm
	1	25 mm - 50 mm and does not interfere with daily activity
	2	51 mm - 100 mm or interferes with daily activity
	3	>100 mm or prevents daily activity
	4	Necrosis
Fever (oral)	0	<38.0°C
	1	38.0°C - 38.4°C
	2	38.5°C - 38.9°C
	3	39.0°C – 40.0°C
	4	>40.0°C
Headache	0	None
	1	Easily tolerated, does not interfere with daily activity
	2	Repeated use of non-narcotic pain reliever for >24 hours or interferes with daily activity
	3	Any use of narcotic pain reliever or prevents daily activity
	4	ER visit or hospitalization
Nausea	0	None
	1	Easily tolerated, does not interfere with daily activity
	2	Interferes with daily activity
	3	Prevents daily activity
	4	ER visit or hospitalization
Vomiting	0	None
	1	1-2 episodes in 24 hours and does not interfere with activity
	2	> 2 episodes in 24 hours or interferes with daily activity
	3	Prevents daily activity or requires outpatient IV hydration
	4	ER visit or hospitalization for hypotensive shock
Myalgia	0	None
	1	Easily tolerated, does not interfere with daily activity
	2	Interferes with daily activity
	3	Prevents daily activity
	4	ER visit or hospitalization
Arthralgia	0	None
	1	Easily tolerated, does not interfere with activity
	2	Interferes with daily activity
	3	Prevents daily activity
	4	ER visit or hospitalization

Urticaria	0<br>1<br>2<br><br>3<br>4	None<br>Requiring no medications<br>Requiring PO or topical treatment, or IV medication or steroids for <24 hours<br>Requiring IV medication or steroids for >24 hours<br>ER visit or hospitalization
Mucocutaneous Reaction/Rash	0<br>1<br>2<br>3<br>4	None<br>Erythema; pruritus or localized macular rash<br>Diffuse, maculo-papular rash, dry desquamation<br>Vesiculation or moist desquamation or ulceration<br>Necrosis

8.2.3 Association with Receipt of the Study Vaccine

All AEs will have their possible relationship to study vaccine assessed using the following terms:

Definite: Clear-cut temporal association, and no other possible cause.
Probable: Clear-cut temporal association and a potential alternative etiology is not apparent.
Possible: Less clear temporal association; other etiologies also possible.
Unlikely: Temporal association between the AE and the vaccine or the nature of the event is such that the vaccine is not likely to have had any reasonable association with the observed illness/event (cause and effect relationship improbable but not impossible).
Not Related: The AE is completely independent of vaccine administration; and/or evidence exists that the event is definitely related to another etiology.

The degree of certainty with which an AE can be attributed to administration of the study vaccine will be determined by how well the event can be understood in terms of one or more of the following:

1. The event being temporally related with vaccination or reproduced on re-vaccination.
2. A reaction of similar nature having previously been observed with this type of vaccine and/or formulation.
3. The event having often been reported in the literature for similar types of vaccines.

All local (injection-site) reactions will be considered related to vaccination.

8.3 Adverse Event Reporting

All SAEs will be reviewed by a study physician, recorded on the appropriate SAE form, and followed through to resolution or stabilization by a study physician. All SAEs will be reported by email, telephone or fax within 1 working day of notification of the SAE occurrence to the Principal Investigator, to the following:

- Sponsor (Office of Vaccine Operations, Sabin Vaccine Institute): Phone: 202-842-5025, Fax: 202-842-7689
- CNMC Institutional Review Board (IRB): Phone: 202-476-5000
- GWUMC IRB: Phone: 202-994-2715, Fax: 202-994-0247

Following notification from the investigator, SVI as the Investigational New Drug (IND) Sponsor, will report events that are both serious and unexpected that are possibly, probably, or definitely related to the vaccine, to the FDA within the required timelines: fatal and life-threatening events within 7 calendar days (by phone or fax) and all other SAEs in writing within 15 calendar days. All SAEs not listed as possibly, probably, or definitely related will be reported to the FDA at least annually in a summary format.

All AESIs will be reported to Sponsor and to the FDA according to the same procedure as for reporting SAEs, and according to the same timelines as described above.

All local and systemic reactions not meeting the criteria for SAEs will be captured on the appropriate Case Report Form (CRF). These events will be followed to resolution. Grade 3 adverse events deemed definitely or probably related to vaccination will be reported by email or fax within 15 working days of the Principal Investigator becoming aware of the event, to the Sponsor and the Safety Monitoring Committee.

8.4 Adverse Event Monitoring

8.4.1 Medical Monitor

An independent medical monitor will be appointed for oversight of participant safety in this trial. The Medical Monitor will be available to advise the investigators on trial-related medical questions or problems. Should the Monitor not be available, they will recommend an alternative to serve as a substitute independent Medical Monitor.

The Medical Monitor's primary responsibility will be to monitor participant safety. The Principal Investigator is responsible for ensuring that the Medical Monitor is aware of any new safety information that becomes available during the course of the trial.

8.4.2 Safety Monitoring Committee (SMC)

SVI will select at least three individuals (one of whom will be the independent Medical Monitor) to advise SVI and the study investigators on the trial. SVI, GWU, and CNMC staff members that are directly involved in this study or that are part of the Human Hookworm Vaccine Initiative partnership are ineligible to serve on the SMC. The SMC's primary responsibility will be to monitor participant safety. The Principal Investigator is responsible for ensuring that the SMC is aware of all new safety information. The SMC will periodically review data on safety and enrollment, and will review cumulative safety data for evidence of study related AEs, adherence to the protocol, and factors that may affect outcome or study data such as protocol violations and losses to follow up.

All cumulative safety data reports from the trial will be submitted to the SMC before GLA-AF dose escalation as described in **Section 4.1** (i.e., between the first dose in Cohort 2 and the first dose in Cohort 3). Safety data reports will include data from at least the first 7 days after vaccination. After the third and final vaccination has been administered to all dose cohorts, additional safety and immunology results and reports will be submitted to the SMC as they become available. A final report will be submitted to the SMC following completion of the study.

Conference calls between the investigators and the SMC will be scheduled within the week prior to beginning vaccinations in the study and prior to each dose escalation as described above. If no criteria for suspending the study are met (see **Section 8.5**), vaccinations will proceed with approval from the SMC. Should the SMC not be able to meet, the SMC may reserve the right to defer to the independent Medical Monitor to decide whether or not vaccinations may proceed.

Written approval (via fax or email) to proceed to the next dose concentration of GLA-AF must be obtained from the SMC (or Medical Monitor in the event that the SMC cannot review the safety data in a timely manner, as described above) prior to administration. Both the SMC and Medical Monitor will have access to the randomization code of the study, as they may wish to review the data in an unblinded fashion should significant safety questions arise prior to the final unblinding.

It is the Principal Investigator's (or designated agent's) responsibility to ensure that the SMC reviews the current safety data (grouped by dose cohort), study protocol, and any other requested documents at its meetings. Occurrence of an SAE will be reported to the SMC at the same time that it is reported to the IRBs. Additionally, any new information that may adversely affect the safety of the subjects or the conduct of the study will be submitted to the SMC as it becomes available.

8.5 Criteria for Suspension of Further Injections

If a dose of vaccine is considered significantly reactogenic (see below), dose escalation and/or additional vaccinations will be suspended until reviewed by the Medical Monitor, SMC and study Sponsor (SVI). Any recommendation of the Medical Monitor and SMC to resume or suspend further injections (either for an individual participant, an entire dose cohort, or all dose cohorts) will be communicated in writing to the sponsor and Principal Investigator. All communications from the SMC will subsequently be forwarded by the investigators to the CNMC and GWUMC IRBs.

The following criteria will be used to define significant reactogenicity (note that these criteria will be applied in advance of any consideration of unblinding):

a. One or more participants experience an SAE (as defined in **Section 8.1.2** in this protocol) that is determined to be possibly, probably, or definitely related to the vaccine (as defined in **Section 8.2.3** in this protocol), **OR**
b. One or more participants experience a hypersensitivity reaction (a Grade 3 allergic reaction, as defined in **Appendix C**) that is possibly, probably or definitely related to the vaccine, **OR**
c. One or more participants in a single dose cohort experience an objective physical finding or laboratory abnormality of Grade 3 or higher (with the exception of isolated Grade 3 erythema or swelling), as defined in **Section 8.2.2** in this protocol, that is determined to be probably or definitely related to the vaccine. **OR**
d. Two or more participants in a single dose cohort experience a Grade 2 or higher safety laboratory abnormality or Grade 3 clinical AE that is possibly, probably or definitely related to the vaccine.

8.6 Treatments That Could Potentially Interfere with Vaccine-Induced Immunity

The following criteria will be checked at each visit. If any are reported during the study, the participant will be excluded from receiving further doses of the study vaccine and will not be included in the immunogenicity evaluations after the time of exclusion. The participant will, however, be encouraged to remain in the safety evaluation for doses already received.

1. Use of any investigational drug or investigational vaccine other than the study vaccine during the study period.
2. Administration of chronic (defined as more than 14 days) immunosuppressants or other immune-modifying drugs up to 30 days after the last dose of vaccine. (Topical and nasal steroids are allowed.)
3. Administration of immunoglobulins and/or any blood products up to 30 days after the last dose of vaccine.

8.7 Criteria for Suspension of Injections

The following criteria will be checked prior to each immunization and are contraindications to further immunization. However, the participant will be encouraged to remain in the safety evaluation for doses already received.

1. Hypersensitivity reaction following administration of a study vaccine.
2. Pregnancy, as determined by a positive urine hCG.
3. Occurrence of any other condition, which in the view of the investigators or Medical Monitor could jeopardize the safety of the study participant or may complicate interpretation of the safety or immunogenicity data.

8.8 Criteria for Deferral of Injections

The following adverse events constitute grounds for deferral of vaccine administration at that point in time; if any one of these occurs at the time scheduled for vaccination, the participant may be vaccinated at a later date, within the allowable time interval specified in **Section 7.55** of this protocol, or withdrawn at the discretion of the investigator. The participant must be followed until resolution or stabilization of the event as with any AE. If the participant is withdrawn from the study, he/she will be encouraged to remain in the safety evaluation for the duration of the study.

1. Oral temperature > 38.0°C at the time of vaccination will warrant deferral of immunization until fever and symptoms resolve.
2. Any other acute condition that in the opinion of the investigator poses a threat to the individual if immunized or that may complicate interpretation of the safety of the vaccine following immunization.

Such individual(s) will be followed until the symptoms resolve or the window for immunization expires. No further vaccination will be performed if the participant does not recover (oral temperature ≤ 38.0°C and/or lack of symptoms) within the originally scheduled vaccination time interval. The participant, however, will be followed for safety and immunogenicity evaluations. If the individual meets any of the above criteria for deferral on the day of <u>first</u> immunization, as

an alternative to deferral of vaccination, the investigator may instead elect to exclude the participant from enrollment in the study. Eligible alternates will then be vaccinated instead.

8.9 Criteria for Withdrawal of an Individual Participant from the Study

A participant will not be considered to have completed the trial if any of the following reasons apply. However, any participant who has received at least one dose of vaccine will be encouraged to remain in the safety evaluation for the duration of the study. Should a female participant become pregnant during the course of the study, she will not receive further doses of vaccine, but will be followed for the duration of the pregnancy.

1. *Research terminated by sponsor or investigator* – applies to the situation where the entire study is terminated by the sponsor or investigator for any reason.
2. *Withdrawal of consent* – applies to a participant who withdraws consent to participate in the study for any reason.
3. *Noncompliant with protocol* – applies to a participant who does not comply with protocol-specific visits or evaluations, on a consistent basis, such that adequate follow-up is not possible and the participant's safety would be compromised by continuing in the trial. Additionally, this applies to a participant who is lost to follow-up and cannot be located.
4. *Develops an adverse event* - applies to a participant who is withdrawn from the study due to an adverse event, serious or otherwise.
5. *Other* – is a category used when previous categories do not apply, and requires an explanation.

8.10 Breaking the Study Blind for Cohorts 1 and 2

As outlined in **Section 4.4**, the study for Cohorts 1 and 2 will be double-blinded until all participants in these two cohorts have completed their Day 140 visit, after which the investigators will be unblinded. During the double-blinded part of the study, a study participant's randomization code may be unblinded only for safety purposes. This is very unlikely to occur, as once a vaccine is administered, knowing which vaccine was given is unlikely to influence the medical management of an AE. This procedure is therefore exceptional and any decision to unblind will be discussed with the sponsor, the Principal Investigator, the Medical Monitor, and the SMC. If deemed necessary for urgent safety reasons, the Medical Monitor, in consultation with the SMC (if possible in a timely manner), may unblind a specific participant without revealing the study blind to the investigators or the Sponsor. Any unblinding will be thoroughly documented. It is to be emphasized that the Medical Monitor may put the study on hold at any time and discuss with the SMC. The decision to completely unblind or permanently stop the study prior to Day 140 will take the form of a formal recommendation by the SMC to the study Sponsor. The Principal Investigator must then notify the IRBs of this decision.

In the event that the investigators come to know the study code of a participant in Cohorts 1 or 2 prior to final unblinding, the Principal Investigator must notify the Sponsor immediately. The reasons will be documented by the Principal Investigator and added to the study file.

Unblinding of the investigators for Cohorts 1 and 2 will be done after all study participants in these two cohorts have completed the Day 140 visit and upon authorization of the Sponsor. This

will take place only after monitoring and verification of cGCP compliance by SVI, and after all safety and primary immunological results (i.e., antigen-specific IgG antibody levels as determined by ELISA) have been entered and the databases locked.

9 DATA COLLECTION AND MONITORING

9.1 Source Documentation and Case Report Forms

Complete source documentation (clinical evaluations and test results) will be collected for every study participant for the duration of the study. In addition, supplementary documents (laboratory test reports, supplementary hospital or medical records, etc.) may form part of the source documentation for a study participant. CRFs will be used to record study-specific data for enrolled participants. The Principal Investigator will be responsible for the accuracy and completeness of the data reported in the CRFs and the source documents. Data reported in the CRFs that is derived from source documents should be consistent with source documents and the discrepancies should be explained.

9.2 Study Documentation

Study-related documentation will be completed as required by the IRB, the Sponsor, and the FDA. Continuing review documentation will be submitted by the Principal Investigator to the IRB on the anniversary date of initial review. An annual report will be submitted by the Sponsor to the FDA within 60 days of the anniversary date that the IND for *Na*-GST-1/Alhydrogel® was submitted.

9.2.1 Study Reports

In addition to the study-related documentation required by the regulatory authorities, two reports will be generated. The first report will be completed after the safety and primary immunogenicity data from the Day 140 visit of all participants have been compiled and the study investigators have been unblinded to the vaccine allocation of Cohorts 1 and 2. This report will serve to inform future studies of *Na*-GST-1, especially with regard to which concentration of GLA-AF might be most immunogenic.

A final report containing all safety and immunology data will be prepared after trial completion and all study-related data have been monitored.

9.3 Retention of Records

Trial-related documents will be maintained by the Investigator for a period of 2 years after final marketing approval of the vaccine, or if 2 years have elapsed since the formal discontinuation of clinical development of the product. The Sponsor is required to inform the Principal Investigator as to when such documents need no longer be retained. Storage of all trial-related documents will be such that confidentiality will be strictly maintained.

9.4 Protocol Revisions

No revisions to this protocol will be permitted without documented approval from both the Sponsor and the CNMC and GWU IRBs. This does not apply to changes made to reduce

discomfort or avert risk to study participants. Furthermore, in the event of a medical emergency, the investigators shall perform any medical procedures that are deemed medically appropriate. The Principal Investigator must notify the Sponsor of all such occurrences. Any change to the protocol will be submitted to the CNMC and GWU IRBs as a protocol amendment, and changes not affecting risk to participants may be expedited, as appropriate.

9.5 Clinical Investigator's Brochure

Investigators will receive the current version of the Clinical Investigator's Brochure, which comprehensively describes all the available preclinical and human experience with the experimental vaccine. If relevant new information becomes available during the course of the trial, the investigators will receive the revised Investigator's Brochure.

9.6 Study Monitoring

The Sponsor (SVI or its designee) will monitor all aspects of the study, with respect to current Good Clinical Practices, and for compliance with applicable government regulations. Prior to the start of the study, the Principal Investigator will be informed of the frequency of monitoring visits and will be given reasonable notification prior to each visit. The objectives of a monitoring visit will be to verify the prompt reporting of SAEs, to check the availability of the signed Informed Consent for enrolled study participants, to compare CRFs and spreadsheets with source data for completeness and accuracy, to verify compliance with the clinical protocol, and to check investigational product accountability. During the monitoring visit, the Principal Investigator (and/or designee) and other study personnel should be available to discuss the study. Study documents must be available for review throughout the course of the study.

10 STATISTICAL CONSIDERATIONS

10.1 General Design

The goal of this Phase 1 vaccine trial is to assess the safety, reactogenicity, and immunogenicity of *Na*-GST-1/Alhydrogel® and *Na*-GST-1/Alhydrogel® co-administered with two different dose concentrations of GLA-AF in healthy adults. These parameters will be assessed in adults without any history of infection with or exposure to hookworm.

10.1.1 Description of the Statistical Methods to Be Employed

The purpose of this trial is to estimate AE rates and patterns of immune response as well as to compare these rates and patterns between the investigational and comparator vaccines, in different doses of the *Na*-GST-1 formulations.

This section briefly describes the statistical methods to be used; a detailed analytical plan will fully describe the methods. The analytical plan will discuss the planned approaches to missing data. Deviations from the original analytical plan will be thoroughly documented and reported to the Sponsor.

Descriptive and hypothesis-testing approaches will be used to meet the protocol objectives as stated in **Section 2.0**. Estimates will be presented with their 95% confidence intervals. Formal

statistical tests, as outlined below, will be used to compare doses. No formal adjustments for multiple comparisons will be made. Statistical tests will use a two-sided significance level of 5%.

Primary Objective: To estimate the frequency of vaccine-related AEs, graded by severity, for each dose and formulation of *Na*-GST-1.

AEs will be coded according to Medical Dictionary of Regulatory Activities (MedDRA™) preferred terms. The frequency, severity, and relationship of AEs for each vaccine formulation and dose cohort will be presented in tabular form using the MedDRA™ coded term and organized by MedDRA™ System, Organ, and Class (SOC) designations.

- The frequency of immediate, systemic, and local injection site AEs will be summarized by SOC and preferred term.
- A line listing of each clinical and laboratory AE classified as immediate (within the first 2 hours), systemic, and local will be displayed in tables stratified by dose cohort and formulation (i.e., no GLA-AF, 1 µg GLA-AF, or 5 µg GLA-AF).
- AEs will be summarized by severity and relationship to vaccine formulation by individuals and dose of *Na*-GST-1.
- The frequency of vaccine-related SAEs will be tallied as well as summarized by body system, by vaccine formulation and dose cohort.

The proportion of participants with at least one injection site AE will be compared by vaccine formulation and by dose cohort.

Laboratory results (hematological and clinical chemistry) will be examined for trends over time and any clinically significant values for individuals will be reported.

Secondary Objective 1: To determine the dose and formulation that generates the highest anti-*Na*-GST-1 antibody response at Day 126.

a. The proportion of participants with detectable anti-*Na*-GST-1 responses will be summarized as a descriptive measure.
b. Geometric mean antibody responses will be compared between vaccine formulation and dose groups. Comparisons between groups will be made by a one-way analysis of variance (ANOVA) with pair-wise comparisons between dose cohorts made by contrasts.
c. For antigen-specific IgG, IgG subclasses (IgG1-IgG4) and IgE levels, a two-way ANOVA will be conducted, with *Na*-GST-1 doses and GLA-AF doses as factors.
d. Pair-wise comparisons across all combinations of *Na*-GST-1 and GLA-AF doses will be assessed using Tukey's HSD.

e. A longitudinal model will be built to describe each of the antibody isotype levels over time for each of IgG, IgG1-IgG4 and IgE. Using a longitudinal panel model, differences in antibody isotype levels by dose and formulation of *Na*-GST-1will be explored. This will be accomplished using Proc Mixed in SAS 9.2 so the analysis will take account of the correlation between measurements on the same participant and provide a robust determination of the covariance structure.

Secondary Objective 2: To determine the dose, formulation, and number of injections of *Na*-GST-1 that generates the antibody response of greatest affinity.

a. A two-way ANOVA with the doses *Na*-GST-1 and GLA-AF as factors and overall avidity as the outcome will be estimated.
b. Pair-wise comparisons across all combinations of *Na*-GST-1 and GLA-AF doses will be assessed using Tukey's HSD.
c. A longitudinal model will be built to describe increasing antibody affinity (affinity maturation) over time. Using a longitudinal panel model, differences in affinity maturation by dose and formulation of *Na*-GST-1 will be explored. This will be accomplished using Proc Mixed in SAS 9.2, so the analysis will take account of the correlation between measurements on the same participant and provide a robust determination of the covariance structure.

Secondary Objective 3: To determine the dose and formulation of the *Na*-GST-1 vaccine that results in the most robust production of *Na*-GST-1 specific B cells and subtypes (memory or plasma).

a. The amount of *Na*-GST-1 specific B cells will be expressed as a percentage of total lymphocytes present in the blood.
b. Two separate longitudinal panel analyses will test the null hypothesis that average percentages for each of memory B and long lived plasma cells are the same in groups with and without co-administration of GLA-AF over time. This will be accomplished using Proc Mixed in SAS 9.2 so the analysis will take account of the correlation between measurements on the same participant and provide a robust determination of the covariance structure.
c. A Mann-Whitney test will assess the null hypothesis that cytokine levels are the same in the two groups (*Na*-GST-1/Alhydrogel® with and without GLA-AF) at the primary time point of Day 126, two weeks after final vaccination.

Secondary Objective 4: To perform exploratory studies of the cellular immune responses to the *Na*-GST-1 antigen both before and after immunization.

The following assays will be performed to assess the cellular immune responses to vaccination with *Na*-GST-1:

a. Lymphocyte proliferative responses to *in vitro* stimulation with *Na*-GST-1.
b. Cytokine and chemokine production *in vitro* in response to stimulation with *Na*-GST-1.
c. Changes in *in vivo* PBMC subpopulations as determined by flow cytometry.
d. A longitudinal panel analysis will test the null hypothesis that cytokine and chemokine levels are the same when *Na*-GST-1 is administered with and without GLA-AF and examine for trends over time.

Should the study be terminated early (see **Section 8.5**), the investigative team will discuss with the SMC the reason for termination and determine which study questions can be addressed in an unbiased manner with the available data. The available data will be analyzed and interpreted in light of early termination.

10.2 Sample Size

This Phase 1 trial is not powered to detect statistically significant differences between groups. Even though comparative statistics for the safety variables will be computed, the study will have low power to detect anything other than very large differences in the incidence of local injection site and systemic side effects between the vaccination groups. This is done weighing the need to detect any possible untoward reactions against the need to limit the number of volunteers involved for safety purposes. The sample size of 40 for this study is within the range commonly used in Phase 1 trials for the initial assessment of the safety, tolerance and immunogenicity of an investigational vaccine.

11 Protection of Human Subjects

11.1 Institutional Review Board Review

The investigators will be responsible for obtaining IRB approval for the study. Before the start of the study, the appropriate documents (including the protocol, Investigator's Brochure, and informed consent form) will be submitted to the IRBs. Full approval for the study will be obtained from the CNMC and GWUMC IRBs.

The study will be conducted according to the Declaration of Helsinki, the US Code of Federal Regulations (Protection of Human Subjects [21 CFR 50], Institutional Review Boards [21 CFR 56], and Obligations of Clinical Investigators [21 CFR 312]).

Modifications to the protocol will not be implemented without prior written IRB approval except when necessary to eliminate immediate hazards to the participants. The IRBs will be informed by the Investigator of any new information that may adversely affect the safety of the subjects or the conduct of the study, an annual update and/or request for re-approval, and when the study has been completed.

11.2 Informed Consent

The principles of informed consent in the current edition of the Declaration of Helsinki will be implemented prior to any protocol-specified procedures being conducted. Informed consent will be obtained in accordance with US 21 CFR 50.25.

Informed consent will be documented by the use of a written consent form approved by the IRBs. All relevant information will be provided in both oral and written form in a way that is understandable to the subject. Ample time and opportunity will be given for the participant to inquire about details of the study.

The Principal Investigator (or the Investigator's designee) will explain the nature of the study and will inform the subject that participation is voluntary and that they can withdraw at any time. The volunteer will be informed about the study's purpose, goals, expected benefits and risks, and potential risks that are currently unforeseeable. They will be provided with a description of the procedures and an estimated duration of time that will be required to participate in the study, and they will be informed of alternatives to participation in the study. The volunteers will receive an explanation as to what medical treatments are available if injury occurs as a result of participation in the study and whom to contact in the event of a study-related injury. They will also be informed whom they should contact for answers to any questions relating to the study. The volunteer will be informed that his/her signature on the informed consent form indicates that he/she has decided to participate in the study having discussed the information presented.

The original signed informed consent form for each volunteer will be maintained as part of the volunteer's study records by the Principal Investigator. A copy of the informed consent form will be provided to every volunteer.

11.3 Risks

Risks to the participants are those associated with venipuncture and with immunization. These risks are outlined below.

Female participants will be cautioned of the unknown risk of the *Na*-GST-1/Alhydrogel® or *Na*-GST-1/Alhydrogel®/GLA-AF vaccines to the fetus. Females of childbearing potential, unless surgically sterile (i.e., bilateral tubal ligation, bilateral oophorectomy, or complete hysterectomy), is at least 2 years postmenopausal, or practices abstinence, must use two effective methods of avoiding pregnancy (including oral, transdermal, or implanted contraceptives, intrauterine device, female condom with spermicide, diaphragm with spermicide, cervical cap, or use of a condom with spermicide by the sexual partner) until at least one month following the last immunization. Female participants will be counseled by a study team member, or referred to the health provider of their choice, for evaluation and institution of an appropriate contraceptive method.

11.3.1 Venipuncture

Risks occasionally associated with venipuncture include pain, bruising, and infection at the site of venipuncture, lightheadedness, and rarely, syncope.

11.3.2 Immunization with Na-*GST-1/Alhydrogel®*

Possible local vaccine reactions include pain, swelling, erythema, induration, transient limitation of limb movement, lymphadenopathy, or pruritus at the injection site. Local subcutaneous nodules, believed to be granulomatous reactions to aluminum hydroxide, have been observed with use of aluminum hydroxide-based adjuvants. Thus, most aluminum hydroxide-adsorbed vaccines are injected intramuscularly rather than subcutaneously. Systemic reactions such as fever, headache, malaise, myalgia, and joint pain, may also possibly occur. Immediate hypersensitivity reactions including urticaria, anaphylaxis, or other IgE-mediated responses are possible as with any vaccine. As with any investigational vaccine, there is a theoretical possibility of risks about which we have no present knowledge. Participants will be informed of any such risks should further data become available.

11.3.3 Immunization with Na-*GST-1/Alhydrogel® plus GLA-AF*

The possible local vaccine reactions to be expected are similar to those of *Na*-GST-1/Alhydrogel® administered without GLA-AF. However, in a study of another vaccine administered with GLA-SE (a formulation similar to GLA-AF), the frequency and intensity of local reactions were somewhat greater than when the vaccine was administered without GLA-SE.

11.4 Precautions taken to Minimize Risks

As outlined above, the participants will be monitored closely during their participation in this study. The study vaccines have been produced according to current Good Manufacturing Practices (cGMP). The vaccines will be administered by experienced investigators with drugs and equipment available for the treatment of anaphylaxis and other potential adverse reactions. All vaccine doses will be given by intramuscular injection to minimize injection site reactions such as pain.

11.5 Benefits

Participants may not receive any direct benefit from participation in this study. It is hoped that information gained in this study will contribute to the development of a safe and effective hookworm vaccine.

11.6 Confidentiality

All study-related information will be stored securely at the study site. All participant information will be stored in locked file cabinets in areas with access limited to study staff. All laboratory specimens, reports, study data collection, and administrative forms will be identified by coded number only, to maintain participant confidentiality. All computer entry will be done by coded number only, and all local databases will be secured with password-protected access systems. Forms, lists, and any other listings that link participant identification numbers to other identifying information will be stored in a separate, locked file in an area with limited access.

Participants' study information will not be released without the written permission of the participant, except as necessary for monitoring by SVI and/or its designee, and the FDA.

11.7 Compensation

Participants will receive total compensation of $590 ($25 per visit for 20 study visits, plus a $90 bonus for completion of all study visits) for their time and inconvenience. Participants will receive one payment at the end of each completed visit, with the bonus dispensed upon completion of the trial.

11.8 Publication of Study Results

It is anticipated that results from this study will be published in peer-reviewed journals. If publication is sought, the identity of study participants or any easily traceable identifiers will not be revealed. Authorship issues will be discussed and agreed upon between the Sponsor and collaborating partners prior to submission for publication. Additionally, the results of the study will be communicated to study participants.

12 REFERENCES

1. Ashford RW, Craig PS, Oppenheimer SJ. Polyparasitism on the Kenya coast. 1. Prevalence, and association between parasitic infections. Ann Trop Med Parasitol. 1992; **86**(6): 671-9.
2. de Silva NR, Brooker S, Hotez PJ, Montresor A, Engels D, Savioli L. Soil-transmitted helminth infections: updating the global picture. Trends Parasitol. 2003; **19**(12): 547-51.
3. Hotez PJ, Brooker S, Bethony JM, Bottazzi ME, Loukas A, Xiao S. Hookworm infection. N Engl J Med. 2004; **351**(8): 799-807.
4. Stoltzfus RJ, Chwaya HM, Montresor A, Albonico M, Savioli L, Tielsch JM. Malaria, hookworms and recent fever are related to anemia and iron status indicators in 0- to 5-y old Zanzibari children and these relationships change with age. J Nutr. 2000; **130**(7): 1724-33.
5. Stoltzfus RJ, Dreyfuss ML, Chwaya HM, Albonico M. Hookworm control as a strategy to prevent iron deficiency. Nutr Rev. 1997; **55**(6): 223-32.
6. Guyatt HL, Brooker S, Peshu N, Shulman CE. Hookworm and anaemia prevalence. Lancet. 2000; **356**(9247): 2101.
7. Brooker S, Bethony J, Hotez PJ. Human hookworm infection in the 21st century. Adv Parasitol. 2004; **58**: 197-288.
8. Brooker S, Peshu N, Warn PA, Mosobo M, Guyatt HL, Marsh K, et al. The epidemiology of hookworm infection and its contribution to anaemia among pre-school children on the Kenyan coast. Trans R Soc Trop Med Hyg. 1999; **93**(3): 240-6.
9. Bundy DA, Chan MS, Savioli L. Hookworm infection in pregnancy. Trans R Soc Trop Med Hyg. 1995; **89**(5): 521-2.
10. Stoltzfus RJ, Chwaya HM, Tielsch JM, Schulze KJ, Albonico M, Savioli L. Epidemiology of iron deficiency anemia in Zanzibari schoolchildren: the importance of hookworms. Am J Clin Nutr. 1997; **65**(1): 153-9.
11. Guyatt HL, Brooker S, Kihamia CM, Hall A, Bundy DA. Evaluation of efficacy of school-based anthelmintic treatments against anaemia in children in the United Republic of Tanzania. Bull World Health Organ. 2001; **79**(8): 695-703.
12. Sakti H, Nokes C, Hertanto WS, Hendratno S, Hall A, Bundy DA, et al. Evidence for an association between hookworm infection and cognitive function in Indonesian school children. Trop Med Int Health. 1999; **4**(5): 322-34.

13. Jardim-Botelho A, Raff S, Rodrigues Rde A, Hoffman HJ, Diemert DJ, Correa-Oliveira R, et al. Hookworm, Ascaris lumbricoides infection and polyparasitism associated with poor cognitive performance in Brazilian schoolchildren. Trop Med Int Health. 2008; **13**(8): 994-1004.
14. Bundy DA, Medley GF. Immuno-epidemiology of human geohelminthiasis: ecological and immunological determinants of worm burden. Parasitology. 1992; **104 Suppl**: S105-19.
15. Bethony J, Chen J, Lin S, Xiao S, Zhan B, Li S, et al. Emerging patterns of hookworm infection: influence of aging on the intensity of Necator infection in Hainan Province, People's Republic of China. Clin Infect Dis. 2002; **35**(11): 1336-44.
16. Sampathkumar V, Rajaratnam A. Prevalence of anaemia and hookworm infestation among adolescent girls in one rural block of TamilNadu. Indian J Matern Child Health. 1997; **8**(3-4): 73-5.
17. Utzinger J, Keiser J. Schistosomiasis and soil-transmitted helminthiasis: common drugs for treatment and control. Expert Opin Pharmacother. 2004; **5**(2): 263-85.
18. Bennett A, Guyatt H. Reducing intestinal nematode infection: efficacy of albendazole and mebendazole. Parasitol Today. 2000; **16**(2): 71-4.
19. Horton J. Albendazole: a review of anthelmintic efficacy and safety in humans. Parasitology. 2000; **121 Suppl**: S113-32.
20. Keiser J, Utzinger J. Efficacy of current drugs against soil-transmitted helminth infections: systematic review and meta-analysis. JAMA. 2008; **299**(16): 1937-48.
21. Hotez PJ, Zhan B, Bethony JM, Loukas A, Williamson A, Goud GN, et al. Progress in the development of a recombinant vaccine for human hookworm disease: the Human Hookworm Vaccine Initiative. Int J Parasitol. 2003; **33**(11): 1245-58.
22. Diemert DJ, Bethony JM, Hotez PJ. Hookworm vaccines. Clin Infect Dis. 2008; **46**(2): 282-8.
23. Bethony JM, Simon G, Diemert DJ, Parenti D, Desrosiers A, Schuck S, et al. Randomized, placebo-controlled, double-blind trial of the Na-ASP-2 hookworm vaccine in unexposed adults. Vaccine. 2008; **26**(19): 2408-17.
24. Ranjit N, Zhan B, Hamilton B, Stenzel D, Lowther J, Pearson M, et al. Proteolytic degradation of hemoglobin in the intestine of the human hookworm Necator americanus. J Infect Dis. 2009; **199**(6): 904-12.
25. Williamson AL, Brindley PJ, Abbenante G, Prociv P, Berry C, Girdwood K, et al. Cleavage of hemoglobin by hookworm cathepsin D aspartic proteases and its potential contribution to host specificity. FASEB J. 2002; **16**(11): 1458-60.
26. Williamson AL, Brindley PJ, Abbenante G, Datu BJ, Prociv P, Berry C, et al. Hookworm aspartic protease, Na-APR-2, cleaves human hemoglobin and serum proteins in a host-specific fashion. J Infect Dis. 2003; **187**(3): 484-94.
27. Williamson AL, Lecchi P, Turk BE, Choe Y, Hotez PJ, McKerrow JH, et al. A multi-enzyme cascade of hemoglobin proteolysis in the intestine of blood-feeding hookworms. J Biol Chem. 2004; **279**(34): 35950-7.
28. Loukas A, Bethony JM, Mendez S, Fujiwara RT, Goud GN, Ranjit N, et al. Vaccination with recombinant aspartic hemoglobinase reduces parasite load and blood loss after hookworm infection in dogs. PLoS Med. 2005; **2**(10): e295.
29. Pearson MS, Bethony JM, Pickering DA, de Oliveira LM, Jariwala A, Santiago H, et al. An enzymatically inactivated hemoglobinase from Necator americanus induces neutralizing antibodies against multiple hookworm species and protects dogs against heterologous hookworm infection. Faseb J. 2009; **23**(9): 3007-19.

30. Zhan B, Liu S, Perally S, Xue J, Fujiwara R, Brophy P, et al. Biochemical characterization and vaccine potential of a heme-binding glutathione transferase from the adult hookworm Ancylostoma caninum. Infect Immun. 2005; **73**(10): 6903-11.
31. Zhan B, Perally S, Brophy PM, Xue J, Goud G, Liu S, et al. Molecular Cloning, Biochemical Characterization and Partial Protective Immunity of the Heme-binding Glutathione S-Transferases from the Human Hookworm Necator americanus. Infect Immun.
32. Asojo OA, Homma K, Sedlacek M, Ngamelue M, Goud GN, Zhan B, et al. X-ray structures of Na-GST-1 and Na-GST-2 two glutathione S-transferase from the human hookworm Necator americanus. BMC Struct Biol. 2007; **7**: 42.
33. van Rossum AJ, Jefferies JR, Rijsewijk FA, LaCourse EJ, Teesdale-Spittle P, Barrett J, et al. Binding of hematin by a new class of glutathione transferase from the blood-feeding parasitic nematode Haemonchus contortus. Infect Immun. 2004; **72**(5): 2780-90.
34. Schuller DJ, Liu Q, Kriksunov IA, Campbell AM, Barrett J, Brophy PM, et al. Crystal structure of a new class of glutathione transferase from the model human hookworm nematode Heligmosomoides polygyrus. Proteins. 2005; **61**(4): 1024-31.
35. Zhan B, Perally S, Brophy PM, Xue J, Goud G, Liu S, et al. Molecular cloning, biochemical characterization, and partial protective immunity of the heme-binding glutathione S-transferases from the human hookworm Necator americanus. Infect Immun. 2010; **78**(4): 1552-63.
36. Xiao S, Zhan B, Xue J, Goud GN, Loukas A, Liu Y, et al. The evaluation of recombinant hookworm antigens as vaccines in hamsters (Mesocricetus auratus) challenged with human hookworm, Necator americanus. Exp Parasitol. 2008; **118**(1): 32-40.
37. Cody CL, Baraff LJ, Cherry JD, Marcy SM, Manclark CR. Nature and rates of adverse reactions associated with DTP and DT immunizations in infants and children. Pediatrics. 1981; **68**(5): 650-60.
38. Merck and Co. I. Recombivax HB® Product Insert. Whitehouse Station, NJ: Merck & Co., Inc.; 2003.
39. Ogutu BR, Apollo OJ, McKinney D, Okoth W, Siangla J, Dubovsky F, et al. Blood stage malaria vaccine eliciting high antigen-specific antibody concentrations confers no protection to young children in Western Kenya. PLoS One. 2009; **4**(3): e4708.
40. Thera MA, Doumbo OK, Coulibaly D, Laurens MB, Kone AK, Guindo AB, et al. Safety and immunogenicity of an AMA1 malaria vaccine in Malian children: results of a phase 1 randomized controlled trial. PLoS One. 2010; **5**(2): e9041.
41. Sacarlal J, Aide P, Aponte JJ, Renom M, Leach A, Mandomando I, et al. Long-term safety and efficacy of the RTS,S/AS02A malaria vaccine in Mozambican children. J Infect Dis. 2009; **200**(3): 329-36.
42. Velez ID, Gilchrist K, Martinez S, Ramirez-Pineda JR, Ashman JA, Alves FP, et al. Safety and immunogenicity of a defined vaccine for the prevention of cutaneous leishmaniasis. Vaccine. 2009; **28**(2): 329-37.
43. Vandepapeliere P, Horsmans Y, Moris P, Van Mechelen M, Janssens M, Koutsoukos M, et al. Vaccine adjuvant systems containing monophosphoryl lipid A and QS21 induce strong and persistent humoral and T cell responses against hepatitis B surface antigen in healthy adult volunteers. Vaccine. 2008; **26**(10): 1375-86.
44. Paavonen J, Naud P, Salmeron J, Wheeler CM, Chow SN, Apter D, et al. Efficacy of human papillomavirus (HPV)-16/18 AS04-adjuvanted vaccine against cervical infection and precancer caused by oncogenic HPV types (PATRICIA): final analysis of a double-blind, randomised study in young women. Lancet. 2009; **374**(9686): 301-14.

45. Konno R, Dobbelaere KO, Godeaux OO, Tamura S, Yoshikawa H. Immunogenicity, reactogenicity, and safety of human papillomavirus 16/18 AS04-adjuvanted vaccine in Japanese women: interim analysis of a phase II, double-blind, randomized controlled trial at month 7. Int J Gynecol Cancer. 2009; **19**(5): 905-11.

Appendix A – Schedule of Visits

Procedures	Blood Volume	Study Day																			
		Pre[2]	0	3	7	14	28	56	59	63	70	84	112	115	119	126	140	170	230	290	470
Complete History/Physical		X																			
Obtain Informed Consent		X																			
Interim Clinical Evaluation			X	X	X	X	X	X	X	X	X	X	X	X	X	X	X	X	X	X	X
CBC[1]	2 mL	X	X			X		X			X		X			X					
PTT/PT INR	2 mL	X																			
ALT		X	X			X		X			X		X			X					
Creatinine	5 mL	X	X			X		X			X		X			X					
Glucose (random)		X																			
Urinalysis		X																			
Urine pregnancy test (females)		X	X					X					X								
HCV serology		X																			
HBsAg serology	10 mL	X																			
HIV serology		X																			
**VACCINATION**			**1**					**2**					**3**								
Anti-*Na*-GST-1 antibody assays	10 mL		X			X	X	X			X	X	X			X	X	X	X	X	
Cellular assays	30 mL		X			X	X	X			X	X	X			X	X			X	
**Blood Volume (mL)**		20	50			50	40	50			50	40	50			50	40	10	10	40	
**Total Blood Volume (mL)**		20	70			120	160	210			260	300	350			400	440	450	460	500	500

[1]CBC parameters to be assessed for safety: WBC, absolute neutrophil count, hemoglobin, and platelet count.
[2]To be completed within 100 days of enrolment.

Appendix B – Toxicity Table for Grading Laboratory Adverse Events

These tables are to be used to assess laboratory adverse events for those tests to be performed as part of the study.

ESTIMATING SEVERITY GRADE

GRADE 1 **Mild:** no effect on activities of daily living; no medical intervention/therapy required

GRADE 2 **Moderate:** partial limitation in activities of daily living (can complete ≥ 50% of baseline); no or minimal medical intervention/therapy required

GRADE 3 **Severe:** activities of daily living limited to < 50% of baseline; medical evaluation/therapy required

GRADE 4 **Potentially life threatening**

**HEMATOLOGY**	**Grade 1**	**Grade 2**	**Grade 3**	**Grade 4**
Hemoglobin<br>*Males*<br>*Females*	 11.2 – 11.9 g/dL<br>9.9 – 10.6 g/dL	 10.2 – 11.1 g/dL<br>8.9 – 9.8 g/dL	 8.5 – 10.1 g/dL<br>7.5 – 8.8 g/dL	 <8.5 g/dL<br><7.5 g/dL
Hemoglobin – (*change from Day 0)*	1.0 – 1.5 g/dL	1.6 – 2.0 g/dL	2.1 – 5.0 g/dL	>5.0 g/dL
Platelets	125,000 – 140,000/mm^3	100,000 – 124,999/mm^3	25,000 - 99,999/mm^3	<25,000/mm^3
WBCs *(increase)*	11,000 – 15,000/mm^3	15,001 – 20,000/mm^3	20,001- 25,000/mm^3	>25,000
WBCs *(decrease)*	2000 – 3000/mm^3	1500 – 1999/mm^3	1000 - 1499/mm^3	<1000/mm^3
ANC *(decrease)*	1000 – 1500/mm^3	750 – 999/mm^3	500 - 749/mm^3	<500/mm^3
**CHEMISTRIES**	**Grade 1**	**Grade 2**	**Grade 3**	**Grade 4**
Serum creatinine	1.5 – 1.7 mg/dL	1.8 – 2.0 mg/dL	2.1 – 2.5 mg/dL	>2.5 mg/dL or requires dialysis
ALT	1.1 – 2.5 x ULN*	2.6 – 5.0 x ULN	5.1 – 10 x ULN	>10 x ULN

*ULN = upper limit of normal (for testing laboratory)

Appendix C – Toxicity Table for Grading Unsolicited Systemic Adverse Events

These tables are to be used to grade unsolicited adverse events not described in **Table 3** of this protocol.

Vital Signs*	Mild (Grade 1)	Moderate (Grade 2)	Severe (Grade 3)	Potentially Life Threatening (Grade 4)
Tachycardia – beats per minute	110-115	116-130	>130	ER visit or hospitalization for arrhythmia
Bradycardia – beats per minute	50-54	45-49	<45	ER visit or hospitalization for arrhythmia
Hypertension** (systolic, mm Hg)	141-150	151-155	>155	ER visit or hospitalization for hypertension
Hypertension** (diastolic, mm Hg)	91-95	96-100	>100	ER visit or hospitalization for hypertension
Hypotension** (systolic, mm Hg)	85-89 (and symptomatic)	80-84 (and symptomatic)	<80	ER visit or hospitalization for hypotensive shock
Respiratory Rate – breaths per minute	18-20	21-25	>25	Intubation
** Participant should be at rest for measurement of vital signs*<br>*** with repeat testing at same visit*				

Systemic AE	Mild (Grade 1)	Moderate (Grade 2)	Severe (Grade 3)	Potentially Life Threatening (Grade 4)
Anorexia	Loss of appetite without decreased oral intake lasting greater than 48 hours	Loss of appetite associated with decreased oral intake without significant weight loss	Loss of appetite associated with significant weight loss	ER visit or hospitalization
Diarrhea	2-3 loose stools/24 hours	4-5 loose stools/24 hours	>6 loose stools or requires outpatient IV hydration	ER visit or hospitalization
Constipation	Not Applicable	Persistent constipation requiring regular use of dietary modifications, laxatives, or enemas	Obstipation with manual evacuation indicated	ER visit or hospitalization
Fatigue	No interference w/activity	Some interference w/activity	Significant, prevents daily activity	ER visit or hospitalization
Arthritis	Mild pain with inflammation, erythema or joint swelling – but not interfering with function	Moderate pain with inflammation, erythema or joint swelling – interfering with function, but not with activities of daily living	Severe pain with inflammation, erythema or joint swelling –and interfering with activities of daily living	ER visit or hospitalization
Vasovagal episode (associated with a procedure of any kind)	Present without loss of consciousness	Present with transient loss of consciousness	Not Applicable	Not Applicable

**Systemic AE**	**Mild (Grade 1)**	**Moderate (Grade 2)**	**Severe (Grade 3)**	**Potentially Life Threatening (Grade 4)**
Vertigo	Causes no or minimal interference with usual daily activities	Causes greater than minimal interference with usual daily activities	Inability to perform daily activities	ER visit or hospitalization
Cough	Transient- no treatment	Persistent cough; treatment responsive	Paroxysmal cough; uncontrolled with treatment	ER visit or hospitalization
Bronchospasm, Acute	Transient; no treatment; 70% - 80% FEV_1 of peak flow	Requires treatment; normalizes with bronchodilator; FEV_1 50% - 70% (of peak flow)	No normalization with bronchodilator; FEV_1 25% - 50% of peak flow; or retractions present	ER visit or hospitalization
Dyspnea	Dyspnea on exertion	Dyspnea with normal activity	Dyspnea at rest	ER visit or hospitalization
Hypersensitivity	Transient flushing or rash	Rash; flushing; dyspnea	Symptomatic bronchospasm, with or without urticaria; parenteral medications(s) administered; allergy-related edema/angioedema; hypotension	ER visit or hospitalization
Illness or clinical AE NOT identified on the toxicity table	No interference with daily activities	Partial limitation in activities of daily living (can complete ≥50% of baseline); no or minimal medical intervention/therapy required	Activities of daily living limited to < 50% of baseline; medical evaluation/therapy required	ER visit or hospitalization
